# Identifying WHO global priority endemic pathogens for vaccine research and development (R&D) using multi-criteria decision analysis (MCDA): an objective of the Immunization Agenda 2030

**DOI:** 10.1016/j.ebiom.2024.105424

**Published:** 2024-11-11

**Authors:** Mateusz Hasso-Agopsowicz, Angela Hwang, Maria-Graciela Hollm-Delgado, Isis Umbelino-Walker, Ruth A. Karron, Raman Rao, Kwaku Poku Asante, Meru Sheel, Erin Sparrow, Birgitte Giersing

**Affiliations:** a World Health Organization, Geneva, Switzerland; b Bridges to Development, Geneva, Switzerland; c Angela Hwang Consulting, Albany, CA, USA; d Department of International Health, Bloomberg School of Public Health, Johns Hopkins University, USA; e Hilleman Laboratories, Singapore; f Kintampo Health Research Centre, Ghana Health Service, Kintampo North Municipality, Ghana; g Sydney School of Public Health, Faculty of Medicine and Health, The University of Sydney, Australia

**Keywords:** Vaccines, Priorities, Research, Development, IA2030

## Abstract

**Background:**

To date, global priorities for new vaccine R&D have not been systematically identified for endemic pathogens. As part of Immunisation Agenda 2030 (IA2030), we have systematically identified priority endemic pathogens for new vaccine R&D based on country and regional stakeholder values to address this need.

**Methods:**

MCDA surveys targeting policy makers and immunisation stakeholders in each World Health Organization (WHO) region were used to weight eight criteria for prioritisation. Applying those weights to regional pathogen data yielded regional top ten pathogen lists, which are intended to inform regional deliberations on R&D priorities. The regional top ten lists were combined into an IA2030 global priority list. To inform R&D, use cases for new vaccines and monoclonal antibodies were identified, then categorized in terms of the activities needed to accelerate progress.

**Findings:**

In five out of six WHO regions, *Annual deaths in children under five* and *Contribution to antimicrobial resistance* were the most heavily weighted criteria. How participants weighted the criteria was not associated with their region, biographical characteristics, or areas of expertise. Five pathogens were common priorities across all regions: *M tuberculosis*, HIV-1, *K pneumoniae*, *S aureus*, and Extra-intestinal pathogenic *E coli*. Six pathogens were priorities in single regions. Combining regional top ten lists provided a global list of 17 priority pathogens for new vaccine R&D. Thirty-four distinct use cases were identified for new products targeting these pathogens. While most are in the “Advance product development” category, ten are in the “Research” category and seven are in the “Prepare to implement” category.

**Interpretation:**

These priorities for new vaccine R&D will help stakeholders better respond to regional and country needs. The use cases will inform R&D and enable monitoring of R&D under IA2030.

**Funding:**

The work was funded by a 10.13039/100000865Bill and Melinda Gates Foundation grant to 10.13039/100004423WHO (INV-005318).

**Research in context**

**Evidence before this study**

To date, global priorities for new vaccine R&D have not been systematically identified for endemic pathogens. Historically, priorities for endemic pathogens have been established by R&D investors, with varying degrees of stakeholder input, typically driven by opportunities for return on investment.

At a regional level, specific vaccine R&D priorities are not commonly stated, but some regional organisations and many countries and funding bodies have stated their vaccine R&D objectives. Vaccine R&D is often discussed more generally in the context of preparedness for emerging infectious diseases, in response to disease outbreaks, or to address antimicrobial resistance. In low- and middle-income countries, these objectives often focus on building clinical trial or manufacturing capacity.

Regional priorities for research and innovation, as stated in the Immunization Agenda 2030 or Global Vaccine Action Plan-related strategic plans, most commonly focus on implementation and operational research, and other research to support vaccine introduction decision-making and maximize the benefits of vaccines or efficiency of vaccination programs.

**Added value of this study**

While some regional and global vaccine priority lists have been developed, none are focused on vaccines in development for endemic pathogens; neither are they based on the systematic assessment of public health and socio-economic criteria that describe the potential value, or benefit, that a vaccine may bring.

This study used multi-criteria decision analysis to survey regional stakeholders on how they value (or “weight”) eight discrete criteria for prioritizing pathogens for vaccine R&D, and then combined the weighted criteria with pathogen data to identify top ten endemic pathogens for each region. These regional lists were combined to form a global priority list of 17 pathogens for new vaccine R&D. This exercise was conducted with regular and thorough consultation with regional experts, and the approach was discussed with WHO’s Strategic Advisory Group of Experts on Immunization. The final list of 17 global priorities and the associated 34 vaccine use cases were endorsed by WHO’s Product Development for Vaccines Advisory Committee.

**Implications of all the available evidence**

The regional top ten lists are intended to inform regional priority setting processes. The global priority list of endemic pathogens should inform regional and global agenda settings for new vaccine R&D and manufacturing, better aligning investments to the evidence base and perspectives of regional and country stakeholders. By aligning immunisation stakeholders, this priority list of endemic pathogens has the potential to advance vaccine R&D and accelerate the benefits of new vaccines. Alongside these R&D priorities, we must work together to strengthen and sustain research and manufacturing capacity in all regions, and ensure that existing and new vaccines reach all those who need them.

## Introduction

Immunization has had an unparalleled impact on global morbidity and mortality, but because vaccine development is technically and commercially challenging, we lack vaccines against many pathogens that continue to impose a substantial public health burden.^1^ Prioritization of pathogen targets for vaccine R&D is therefore crucial for the efficient use of limited resources, to ensure alignment with public health needs, and maximise health benefits. The WHO R&D Blueprint has identified priority pathogens with epidemic potential,^2^ but no such global prioritization has been undertaken for endemic pathogens (i.e., pathogens that cause disease regularly within a given population).

Diverse epidemiological and social contexts demand a nuanced approach to vaccine prioritization that takes into account regional differences. This is increasingly the case, given that few truly global pathogens remain without vaccines: many vaccines in development today will be used in and may be manufactured in specific regional settings. Existing priority lists are narrower in scope^3^^,^^4^ and are typically driven by the opinions of global rather than local disease experts.^4^^,^^5^ A systematic, transparent, and inclusive approach to prioritizing endemic pathogens for vaccine development is imperative, one that is informed by a broad range of perspectives and is responsive to both global and regional public health needs.

This study was conducted in support of Immunization Agenda 2030.^6^^,^^7^ Its aim was to prioritise global endemic pathogens for new vaccine R&D using an evidence-based approach that recognises the complex interplay of epidemiological, economic, and social factors influencing vaccine R&D priorities at a regional level. In that way, this work is intended to contribute to a more equitable and effective global health agenda by informing policy decisions, guiding R&D investments, and fostering international collaboration in vaccine development.

## Methods

**Fig. 1:**
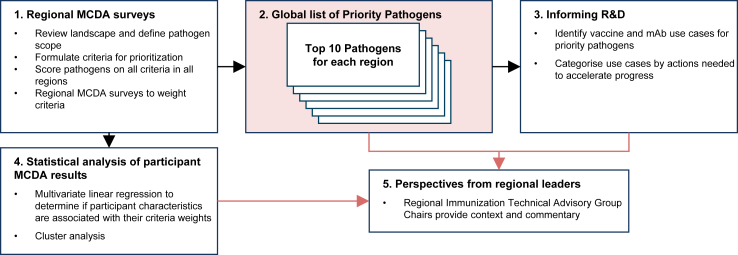
Overview of the process to identify WHO global priority endemic pathogens for vaccine R&D. Abbreviations: MCDA, multi-criteria decision analysis; R&D, research and development; mAb, monoclonal antibody.

Fig. 1 gives an overview of the study, which was conducted in five stages.

### Stage 1: regional MCDA surveys

MCDA was used to prioritize endemic pathogens for vaccine R&D. MCDA was selected for its demonstrated ability to support health-related decision-making in the context of multiple trade-offs and diverse perspectives.^3^^,^^8^

To implement MCDA, we first determined which pathogens to include in the exercise. A landscape review (Box 1) identified over 150 pathogens that could be considered for vaccine R&D (Appendix, page 6). From these, pathogens were included in the MCDA if they caused disease in humans; had candidate vaccines or monoclonal antibodies (mAbs) in clinical development (to focus on products with a higher potential for success); and where vaccine R&D had been called-for by disease-specific strategies. Pathogens suggested by regional advisors to WHO were also included. Pathogens were excluded if they had a licensed vaccine unless those vaccines did not meet identified public health needs. Emerging infectious disease pathogens, which require different prioritization criteria, were also excluded. These pathogens are addressed by other prioritization exercises, such as the WHO R&D Blueprint.^2^ Overall, 26 pathogens met these criteria and were included in the MCDA (Table 1). This short-list was reviewed by members of the WHO Product Development Vaccine Advisory Committee (PDVAC) and the WHO Strategic Advisory Group of Experts on Immunisation (SAGE).

**Box 1** **Search strategies.**

**Existing R&D priorities**

The review of existing priorities was conducted through searches of PubMed (pubmed.ncbi.nlm.nih.gov), the WHO Institutional Repository for Information Sharing, the National Immunization Technical Advisory Groups Resource Center, and websites for selected national agencies for health research. Search terms included “national health research strategy.” Searches were conducted in English from March to June 2022. This review was supplemented with national immunisation strategies submitted in the 2022 annual electronic Joint Reporting Form process.

**Criteria for prioritisation**

Precedents for criteria for prioritization were compiled from multiple sources. First, during the review of existing priorities, any stated criteria for prioritization were documented. These were combined with criteria used to set vaccine-related research priorities, vaccine R&D priorities, and vaccine introduction priorities. Such priorities were identified through internet searches, particularly of PubMed and the WHO website, conducted from March to June 2022. Search terms included, “vaccine”, priorities”, and “criteria”. Finally, attributes documented in Vaccine Value Profiles were included, since they have been identified by PDVAC as relevant to vaccine R&D decision making.

**Pathogens**

Pathogens were identified from the review of existing R&D priorities, and included pathogens prioritized for vaccine R&D, for research, or for surveillance. Additional pathogens were identified by searching for vaccine trials on ClinicalTrials.gov and the International Clinical Trials Registry Platform (ICTRP), and from Health Topics on the WHO website, an analysis of investments in global health research, and Wikipedia.

**Pathogen scoring**

Data to inform the scoring were compiled from PubMed queries and targeted internet searches in English. To give a more balanced picture, PubMed queries were focused on systematic reviews and on Vaccine Value Profiles. Detailed sources for pathogen scores are given in the Appendix, along with coding of scores based on data availability.

**Use Cases**

The WHO web site was reviewed to identify vaccine position papers, preferred product characteristics, and other guidance relating to pathogens on the global list. Additional potential use cases were identified through targeted PubMed searches, review of clinical trial designs and developers’ public statements relating to candidates in development, and review of WHO and partner documents. Sources for use cases are given in the Supplemental materials.

**Progress in clinical development**

Candidates in clinical development were identified through searches of the ICTRP conducted in October and November 2023. Broad search criteria were used (“vaccine” or “monoclonal antibody” and phase 1, 2, or 3 trials) and relevant trials were identified through review of study records, supported by review of primary registries as needed.

We assumed that the candidates were no longer active if trial results remained unpublished for more than 3 years after the study concluded, no follow-on studies have been registered, and regulatory filings had not been announced.

**Table 1:** Key elements of MCDA.

Pathogens evaluated	
Chikungunya virus *Chlamydia trachomatis*^a^ Cytomegalovirus Dengue virus Extra-intestinal pathogenic *E coli* (ExPEC) Group A streptococcus (*Streptococcus pyogenes*) Group B streptococcus (*Streptococcus agalactiae*) Hepatitis C virus^a^ Herpes simplex virus types 1 and 2 Hookworm Human immunodeficiency virus 1 (HIV-1) Influenza virus Intestinal pathogenic *E coli* (InPEC)	*Klebsiella pneumoniae* *Leishmania* species *Mycobacterium leprae* (leprosy) *Mycobacterium tuberculosis* (TB) *Neisseria gonorrhoeae* Non-typhoidal *Salmonella* Norovirus *Plasmodium falciparum* (malaria) Respiratory syncytial virus *Salmonella* Paratyphi Schistosomes *Shigella* species *Staphylococcus aureus*
**Criteria for prioritisation**	

Scored quantitatively	Scored qualitatively

*Annual deaths in children under 5:* Deaths attributable to the pathogen in both sexes, <5 years old *Annual deaths in people older than 5:* Deaths attributable to the pathogen in both sexes, ≥5 years old *Yearslived withdisability (all ages):* Years lived with any short-term or long-term health loss caused by the pathogen, weighted for severity	*Social and economic burden* per *case*: Reflects individual social and economic impact such as stigma and the costs of prevention, health care, and lost productivity *Disruption due to outbreaks:* Reflects societal impact due to outbreaks and epidemics, including social disruption; impact on healthcare systems, trade or tourism; and the cost of containment measures *Contribution to inequity:* Reflects disproportionate impact on socially and economically disadvantaged groups, including women *Contribution to antimicrobial resistance (AMR):* Reflects the threat of resistance, based on current levels of resistance, contribution to antibiotic use, and designation as an AMR priority *Unmet needs for prevention and treatment:* Reflects the effectiveness and suitability of alternative measures

Pathogen inclusion criteria are given in Appendix page 6. Detailed information on the criteria for prioritisation are given in Appendix page 8.

a Added at the request of regional stakeholders.

Second, we formulated eight criteria for prioritization (Table 1) based on relevant precedents in priority setting for research, funding, and vaccine introduction (Box 1).^9^^,^^10^ Criteria aimed to be comprehensive while adhering to MCDA good practice guidelines.^11^ Five levels for each criterion, ranging from Very low to Very high, were defined with feedback from MCDA experts and PDVAC members (Appendix, page 5).

Third, pathogens were scored on each criterion. For each region, each pathogen was scored in comparison to the other pathogens included in this exercise. (Because of this, scores should not be compared across regions.) For the quantitative criteria, *Annual deaths in children under 5 years*, *Annual deaths in people older than 5 years*, and *Years lived with disability (all ages)*, pathogens were scored using data from the 2019 Global Burden of Disease study (GBD 2019).^12^^,^^13^ For AMR pathogens, values used were the total burden of antibiotic resistant and susceptible forms.^13^ Where GBD 2019 did not include required data, scores were assigned under expert guidance based on estimates from other sources.^14^^,^^15^ Systematic burden estimates were not found for cytomegalovirus, so quantitative scores for cytomegalovirus must be considered preliminary.

The remaining criteria were scored qualitatively using a pathogen scoring guide (Appendix, page 8). Evidence for scoring was extracted for each WHO region (Box 1). Where region-specific data were not found for a particular criterion, scores were inferred based on data obtained in other regions. For transparency, scores were coded to indicate whether they were based on regional data or inferred (Appendix, page 10). Data extraction was conducted by two analysts (AH and MVD) and qualitative scoring was conducted independently by three analysts (AH, AP, MVD). Their consensus scores were reviewed by at least one pathogen expert and two experts per region.^14^^,^^15^ Resulting scores are shown in the Appendix, pages 11–16.

Finally, cross-sectional MCDA surveys were conducted to rank pathogens in each region. Surveys were created with 1000minds software (1000minds Ltd., Dunedin, New Zealand), which uses the PAPRIKA (Potentially All Pairwise RanKings of all possible Alternatives) method.^16^ Surveys were prepared in English and translated into Arabic, Chinese, French, Portuguese, Russian, and Spanish. Translations were reviewed by native speakers with immunisation expertise.

Survey participants were identified through referral sampling. Invitations and reminders to participate in the surveys were emailed by WHO to immunisation stakeholders at regional and country levels (Appendix, pages 17–22). Stakeholders included policy makers, health practitioners, and other experts in public health and immunisation. Invitations encouraged recipients to share the survey with colleagues: this helped to increase the number of responses but precludes calculation of response rates. Emails were sent in multiple waves starting in November 2022. The survey remained open until the 1st of May 2023.

Each survey began with biographical questions. Personal information was collected for identification purposes only, maintained in password-protected document stores, and not shared beyond the research team. Participants were then asked a series of discrete choice questions in which they selected which of two hypothetical pathogens they would prioritise for vaccine R&D (Appendix, pages 23–26). Pathogens were described using two criteria at a time. Successive questions gave additional pairwise combinations of criteria to choose between. Once choices were complete, the software computed the relative importance (or “weight”) of each criterion, displayed the participant’s individual pathogen ranking, and asked for feedback on the survey (Appendix pages 27–30). No reimbursement was provided for participation.

### Stage 2: global list of priority endemic pathogens for vaccine R&D

Participant names and email addresses were manually screened to identify duplicate responses. Incomplete and duplicate survey responses, and responses where the participant selected the same answer for every question, were excluded from analysis. PAPRIKA expresses criteria weights as percentages, such that a hypothetical pathogen which scored Very high for all criteria would have a total weight of 100%. Regional criteria weights were calculated by averaging individual criteria weights from each WHO region. Total weights for each pathogen were calculated by summing criteria weights corresponding to regional pathogen scores (Appendix, page 27).

Ten pathogens with the highest total weight from each WHO region were included in the global priority pathogen list. To assess robustness of the global list, it was recalculated omitting one criterion at a time.

### Stage 3: informing R&D

We then identified use cases to address each of the priority pathogens. Use cases consisted of the target population and conditions to be prevented or alleviated through use of a new vaccine. We also identified use cases for mAbs targeting these pathogens if they had similar properties to the vaccines (e.g., same conditions to be prevented) because of their potential for synergies in investment and their potential to address the same burden as vaccines. Use cases were identified by reviewing WHO documents, from published literature, and from product developer strategies (Box 1). To focus on key public health needs, use cases that were considered to be met by existing vaccines as of November 2023 (such as seasonal influenza vaccines and vaccines to reduce malaria morbidity and mortality) as well as those that are highly personalized (such as cytomegalovirus vaccines for transplant recipients) were excluded. Therapeutic products were included if called-for by WHO guidance or if they have the potential for widespread use. Use cases were consolidated where clinical development is in early stages and preferred product characteristics have not been defined.

The use cases were then stratified into three categories based on progress in clinical development and probability of technical and regulatory success: (1) “Research” for use cases that have few candidates in clinical development and are facing substantial technical challenges; (2) “Advance Product Development” for use cases with a strong clinical development pipeline and vaccine candidates with medium to high probability of technical and regulatory success; and (3) “Prepare to Implement” for use cases with candidates in phase 3 clinical trials and high potential for licensure in the near future. Category definitions and recommended actions were developed by the research team. Category assignments were reviewed and endorsed by PDVAC in December 2023.

### Stage 4: statistical analysis of participant MCDA results

Categorical data were presented as numbers and percentages. Continuous data were summarized as means with standard deviation or median with interquartile range. Differences in proportions and means were assessed using Chi-square/Fisher’s Exact (FE) Test and t-test, respectively.

Adjusted mean differences in criteria weights by participant and survey characteristics were estimated using multivariate linear regression with the R package ‘stats’ (version 4.3.1). Characteristics of interest included participant’s years of experience (reference: up to 10 years); expertise in each of six areas (reference: not in the listed area); employment in each of nine types of organisations (reference: does not work for specified type of organisation); 2023 eligibility for Gavi support (reference: not eligible) and World Bank group classification (reference: high income) for the participant’s country of work; language of survey (reference: English); WHO region of survey (reference: Africa); whether the survey was easy or difficult to understand (reference: neutral); if order of criteria weights were correct (reference: yes); and, if pathogen rankings were reasonable (reference: yes).

Group patterns in criteria weights, or “clusters”, were identified using K-means algorithm with the R packages “factoextra” (version 1.0.7) and “NbClust” (version 3.0.1). To avoid issues of collinearity, a composite indicator of weights (based on Medium and Very High levels for each of the eight criteria) was first generated using Principal Components Analysis (PCA) using the R packages ‘FactoMineR’ (version 2.11) and “factoextra” (version 1.0.7). The composite indicator included the first set of eigenvectors accounting for at least 80% of the explained variance in the PCA model. Optimal number of clusters were then determined based on consensus of cut-off values using the elbow method, silhouette coefficient, Gap statistic and NbClust with centroid values ranging from 0 to 25.^17^, ^18^, ^19^, ^20^

Based on a two-cluster model (optimal number determined in k-means analysis), a generalized linear model (GLM) with a binomial distribution and logit link function was used to compare odds of membership in a specific cluster by years of experience, language and WHO region of survey, field of expertise, eligibility for Gavi support and World Bank income group classifications for country of work, type of work organisation, and face-validity assessment. GLM was performed using the R package ‘stats’ (version 4.3.1). Statistical power calculations suggest that the minimum detectable odds ratio for membership in a specific cluster (based on two-cluster model) is between 0.213 and 2.475. The estimate was based on a sample size of 95 subjects in one cluster, 178 subjects in a reference cluster, a 15% likelihood of presenting a specific background characteristic, 80% statistical power and a two-sided significance level of 0.05.

Our primary analysis was limited to the first survey submission (per survey type) by a participant in which all trade-off questions and the post-survey had been completed. Page 31 of the Appendix gives an overview of survey responses at each stage of the analysis. To assess for potential selection bias due to incomplete surveys, generalized estimating equation (GEE) with a binomial distribution and logit function was used to measure associations between participant characteristics and completion of trade-off questions, accounting for multiple responses per person. GEE was performed using the R package ‘gee’ (version 4.13-25).

Statistical analyses were completed using R version 4.2.2 (R Foundation for Statistical Computing, Vienna, Austria).

### Stage 5: perspectives from regional leaders

Finally, we asked regional leaders to contextualise and comment on these results. Regional leaders consisted of Chairs for the six regional immunisation technical advisory groups (RITAGS).

### Ethics

Under WHO procedures, this study was exempt from ethical review because (1) research activities pose minimal risk to survey participants and (2) survey participants were asked to provide only objective information or professional opinion on a topic under their direct responsibility or expertise, with their expressed knowledge and consent, and without providing any private individual opinions or opinions on matters outside their direct expertise.

### Role of funders

The funder of the study had no role in study design, data collection, data analysis, data interpretation, or writing of the report. The corresponding author had full access to all the data in the study and had final responsibility for the decision to submit for publication.

## Results

### MCDA survey responses

In total, 577 survey responses were received, of which 49% (284/577) were complete (Appendix, page 30). Of complete responses, 11 invalid responses were excluded, leaving 273/577 (47%) responses for analysis. Participants reported working in 89 countries, or 46% (89/194) of WHO member states (Appendix, page 31). Out of included responses, 25% of participants (68/273) worked in countries eligible for support from Gavi, the Vaccine Alliance. Among included responses, participants’ most common areas of expertise were disease epidemiology (52% or 143/273) and vaccine R&D (45% or 123/273). Expertise in health policy was reported by 39% (80/273) of included participants. The most common organisation types were academic (39% or 107/273) and government (33% or 90/273). There were some differences in participants’ characteristics across the regions. Most notably, compared to participants of African surveys (0/55 or 0%), a greater proportion of survey participants worked for the pharmaceutical industry in the American (9% or 4/45, FE p = 0.04), European (12% or 3/26, FE p = 0.03), and Western Pacific (20% or 13/65, FE p < 0.01) regions (Appendix, page 32).

### Criteria weights and pathogen priorities

**Fig. 2:**
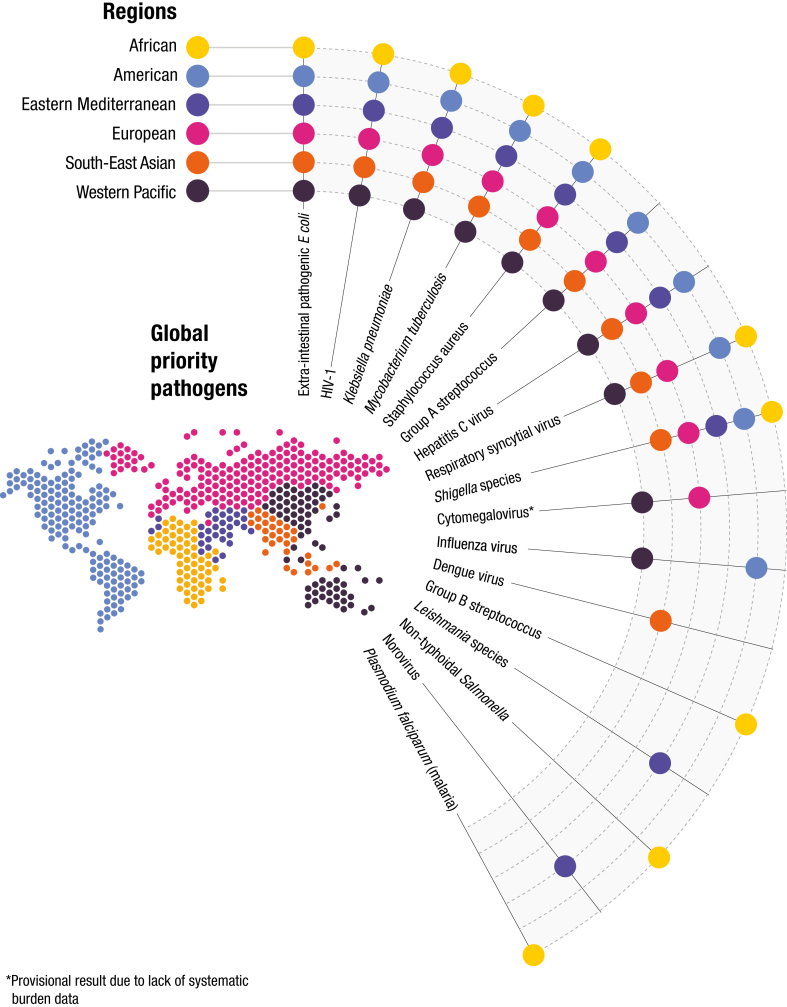
Global Priority Pathogens for Vaccine R&D. Dots indicate where each pathogen appears on regional top 10 lists. Regional results are intended to inform deliberations and should not be read as regional priorities in themselves. ∗Cytomegalovirus results are provisional due to lack of systematic burden estimates. (See Appendix, page 35, for ranking of individual pathogens in each region.)

Fig. 2 shows the global list of priority endemic pathogens for new vaccine R&D. This list is comprised of the ten pathogens with highest weights for each region. Pages 34–35 of the Appendix shows all pathogens with their total weights in each WHO region. Of the 17 pathogens on the global priority list, five (*M tuberculosis*, HIV-1, *K pneumoniae*, *S aureus*, and ExPEC) appear on the top ten list for every region. Remaining pathogens illustrate the diversity across regions.

Robustness of the global list was assessed by omitting single criteria from the calculation (Appendix, page 36). Of the 17 pathogens on the global list, 13 were not affected by the omission of any one of the eight criteria for prioritization. Omitting single criteria removed dengue virus, GBS, norovirus, or RSV from the global list in some instances and caused the inclusion of InPEC, *M leprae*, or *N gonorrhoeae* in some instances. Pathogens dropped from the global list were those that were on fewer regional top ten lists and ranked lower on those lists.

Appendix, page 34 shows mean weights for all criteria. In five out of six regions, *Annual deaths in children under 5 years* was the most heavily weighted criterion and *Contribution to AMR* was the second most heavily weighted criterion, with mean weights ranging from 14.0 (Standard Deviation (SD): 8.2) percent to 18.6 (SD: 7.8) percent and from 13.6 (SD: 6.5) percent to 15.5 (SD: 7.8) percent, respectively. In the remaining region, the Western Pacific, the two highest rated criteria were *Annual deaths in people 5 years and older* and *Annual deaths in children under 5 years*, with mean weights of 14.9 (SD: 8.4) percent and 14.3 (SD: 8.0) percent respectively. Appendix, pages 43–44 shows that regional differences in criteria weights were minimal. Two regions (European and Western Pacific) differed from the African region in the criterion for *Disruption due to Outbreaks* (European: adjusted Mean Difference (aMD): −6.95, 95% Confidence Interval (CI): −12.02 to −1.87; and Western Pacific: aMD: −4.8, 95% CI: −8.85 to −0.32). However, all remaining regional differences were not significantly different when compared to the reference region. Criteria weights were not associated with background traits of participants in most cases (Appendix, pages 45–65).

### Use cases and action categories

Across the 17 priority pathogens, 34 unmet use cases were identified for new vaccines and mAbs of public health importance (Appendix, pages 38–40). Of these, 38% (13/34) currently have relevant candidates in phase 3 trials.^21^ While no currently licensed vaccines have policy recommendations corresponding to these use cases, as of April 2024 recommendations are under consideration for 2/34 (6%) use cases with recently licensed products.

Of these use cases, ten are in the “Research” category, 17 are in the “Advance Product Development” category, and seven in the “Prepare to Implement” category (Appendix, page 38). Table 2 shows the distribution of priority pathogens across the three categories based on the most advanced use case for each pathogen.

**Table 2:** Action categories for global priority pathogens for vaccine R&D, based on the most advanced unmet use case for each pathogen as of December 2023.

Categories are a continuum and vaccine use cases often span categories. Research and product development continue throughout the product lifecycle. Category definitions and recommended actions were developed by the research team. Category assignments were reviewed and endorsed by PDVAC in December 2023.

### Cluster patterns detected among criteria weights

Cluster analysis showed regional survey responses fell into one of two clusters (Appendix, page 66). Cluster 1, representing 65% (178/273) of responses, gave greatest weight to *Contribution to AMR* and to *Disruption due to outbreaks*. Cluster 2, representing 35% (95/273) of responses, gave greatest weight to *Annual deaths in children under 5* and *Annual deaths in people 5 years and older*. Appendix, pages 67–68 show that cluster membership could not be predicted based on participant characteristics such as areas of expertise, type of organisation, or whether the participant works in a Gavi-eligible or high-, middle-, or low-income country.

Both clusters gave very similar priority lists (Appendix, page 37). When only Cluster 1 responses were used to calculate priorities, GBS no longer appeared on the global list. When only Cluster 2 responses were used, dengue virus and norovirus no longer appeared on the global list. The other 14 pathogens on the global list were not affected.

### Perspectives from regional leaders

A consensus statement from the six RITAG Chairs is given in Box 2.

**Box 2** **Key messages from regional leaders about WHO global priority endemic pathogens for vaccine R&D.**

• This is the first global prioritization exercise for endemic pathogens, and it complements other WHO pathogen prioritization exercises, such as that recently conducted by the WHO R&D Blueprint for pathogens with epidemic potential.^2^
• The outcome is a list of 17 priority global endemic pathogens. The methodology applied ensures that these priorities reflect not only burden in terms of cases and deaths, but also the values of stakeholders in different settings with respect to impact of long-term sequelae on quality of life, contribution to inequity and the broader socio-economic toll of a pathogen. The approach is a departure from the conventional “top-down” prioritization largely driven by the interests of people from high-income countries.
• This prioritization exercise presents an opportunity to leverage, strengthen, and sustain the broader R&D ecosystem that is needed to advance vaccine development across the national, regional and global levels. We must build and sustain capacity in all regions to conduct basic research; to develop and evaluate new vaccines; and to manufacture, regulate, introduce, and sustain coverage with new and existing vaccines.^22^ Many efforts are underway to address the inequities highlighted by COVID-19 pandemic. By harnessing these efforts for regionally defined priorities we can help address endemic diseases and build the resilience needed to address future threats.
• Critically, we must reach and immunise the 14.3 million zero-dose children and the many more under-vaccinated children worldwide.^23^ Countries struggle to strengthen and sustain routine immunisation, particularly in the communities that are most poorly served. They also face difficult choices with infant vaccination schedules that are already crowded, existing vaccines that are under-utilized, and new vaccines on the horizon for additional age groups and sub-populations. In this context, combination vaccines and innovations that improve acceptability, ease administration, or facilitate delivery to remote areas or during outbreaks are increasingly important and urgently needed.
• As a community, we can and must tackle these challenges together, and quickly, to fully realise the benefits and enable sustainable impact of existing and future vaccines. By heeding these calls to action, we can steer towards the IA2030 vision of “A world where everyone, everywhere, at every age, fully benefits from immunisation to improve health and well-being.”^6^

*By:* Rakesh Aggarwal, Chair, South-East Asia Regional Immunization Technical Advisory Group; Peter Figueroa, Chair, Pan-American Health Organization (PAHO) Vaccine-preventable Diseases Technical Advisory Group (TAG); Christopher Morgan, Chair of the Technical Advisory Group on Immunization and Vaccine-Preventable Diseases in the Western Pacific Region; Ezzeddine Mohsni, Chair, Eastern Mediterranean Regional Technical Advisory Group on Immunization; Helen Rees, Chair, African Regional Immunization Technical Advisory Group; and Ole Wichmann, Chair, European Technical Advisory Group of Experts on Immunization.

## Discussion

Using a systematic, transparent, inclusive, and regionally focused approach, we have identified global endemic pathogen priorities for new vaccine R&D as part of the IA2030 objectives. The list of 17 priority pathogens includes pathogens that affect people of all ages and income levels. It affirms some longstanding priorities for vaccine R&D, such as HIV-1 and tuberculosis, and has strengthened the case for less recognized priorities for vaccine R&D, such as *Shigella* and *K pneumoniae.* This list is robust, changing minimally as criteria are omitted or different clusters of responses are used to generate the list.

We have also identified 34 use cases for new vaccines and mAbs targeting these pathogens. These use cases are diverse, with candidates at varying stages of development and differing in probability of technical and regulatory success, as well as in feasibility of policy and uptake. Our aim in grouping them into action categories was to bring order to this diversity, helping to encourage investment, inform collaborations, and focus resources on R&D for new vaccines and mAbs that better respond to regional needs. Because the use cases and action category assignments will evolve as R&D progresses, they will be the basis for monitoring R&D progress under IA2030.

This approach has highlighted the epidemiological and socio-economic differences between regions and the importance of considering each pathogen in the context of each region. Of the 17 pathogens on the global priority list, one-third are single-region priorities. Assembling the global priority list from regional top ten lists captures this diversity and ensures that pathogens that significantly affect regional health but do not pose a global threat are not overlooked.

While priority pathogens differ between regions due to differences in epidemiology, survey participants have similar values when setting pathogen priorities, regardless of WHO region, areas of expertise, country eligibility for Gavi support, and many other attributes. Annual deaths are widely seen as important criteria, consistent with the expectation that deaths are a major driver of vaccine value. Participants also identified contribution to AMR as an important criterion. This underscores the growing recognition of the role of vaccines in reducing the AMR burden.

These priority endemic pathogens and use cases are intended to inform the strategies of global and regional immunisation stakeholders, including funders, researchers, product developers, and policy makers. By giving a cross-cutting perspective, the global list of endemic pathogen priorities for vaccine R&D complements existing WHO vaccine priorities, or global strategies that focus on specific issues,^24^, ^25^, ^26^, ^27^ such as the prioritization of emerging (rather than endemic) pathogens by the WHO R&D Blueprint for Epidemics.^2^ WHO will continue to support advancing development, introduction, and update of vaccines with high public health value in low- and middle-income countries. The results are already informing the strategies of global immunisation partners such as the Gavi Alliance and regional consultations on research priorities.

In considering these lists, some limitations must be recalled. The regional top ten lists must be viewed as evidence-based research outputs intended to inform regional priority-setting processes. While the list is meant to provide official WHO global recommendations for endemic vaccine R&D, the regional stakeholders will need to set regional priorities based on considerations such as funding and partnership opportunities, infrastructure of the immunization programmes, the costs of R&D, and potential value propositions that were beyond the scope of our approach.

Importantly, the global priority list is strongly driven by the pathogen scores. Because we used GBD 2019 data, scoring of criteria 1–3 does not reflect effects of COVID-19 or changing circumstances such as increased rates of dengue. GBD also has important limitations, including scant data from many lower-income settings.^28^ Data for scoring pathogens on the remaining criteria also varied by region and by pathogen and, due to time and resource constraints, systematic reviews were not feasible and literature searches were conducted only in English. Addressing inequities in pathogen data and updating systematic estimates for pathogen burden are essential for improving priority setting.

Lastly, because the target audience of policy makers and other immunisation stakeholders is a broad population in public health decision making, we were unable to thoroughly assess the representativeness of survey participants. Notwithstanding, our cluster analysis of MCDA results showed that patterns in survey responses were not linked to participant characteristics, suggesting that sampling bias and generalizability of results may not be as problematic with MCDA as other more conventional survey approaches.

The approach also has important strengths. This prioritisation has been guided by the IA2030 core principles of “Data-guided, People-centred, Partnership-based, and Country-owned.”^29^ It has strived for inclusiveness, including through use of MCDA, by making surveys available in the major languages for each region, and by disseminating survey invitations through diverse regional networks. The MCDA method is highly structured, lending itself well to systematic criteria definitions and pathogen scoring. The surveys enabled inclusion of a wide range of stakeholders while minimizing pathogen-related biases. MCDA also allows priorities to be updated as the pathogen landscape evolves, by applying criteria weights from these surveys to updated pathogen data.

The experience and insights gained through this work can be applied to future prioritisations, such as prioritising pathogens for preclinical research, setting priorities for improving existing vaccines and creating combination vaccines, and setting implementation and operational research priorities to more efficiently reach all those who need vaccines.

By acting on these priorities, immunisation stakeholders everywhere can align their efforts to address the most significant infectious disease challenges and maximise the benefits of vaccines.

## Contributors

Research team: Mateusz Hasso-Agopsowicz: conceptualisation, methodology, writing—original draft, writing—review & editing. Angela Hwang: conceptualisation, data curation, formal analysis, investigation, methodology, visualisation, writing—original draft, writing—review & editing. Maria-Graciela Hollm-Delgado: formal analysis, writing—original draft, writing—review & editing. Isis Umbelino-Walker: investigation, writing—review & editing. Erin Sparrow: conceptualisation, methodology, writing—review & editing. Birgitte Giersing: conceptualisation, funding acquisition, methodology, supervision, writing—review & editing.

Project guidance: Ruth Karron, Raman Rao, Kwaku Poku Asante, and Meru Sheel: writing—review & editing.

Mateusz Hasso-Agopsowicz and Angela Hwang have accessed and verified the underlying data.

All authors read and approved the final version of the manuscript.

## Data sharing statement

Anonymized survey responses are included in the Supplementary materials. Data used to score pathogens, pathogen scores, averaged criteria weights, demographic data of participants, all stratified by regions, are available in the manuscript or Appendix. Beyond the manuscript and the Appendix, additional related documents are posted on the Technet-21 platform at: https://www.technet-21.org/en/topics/global-initiatives/regional-and-country-priorities-for-vaccine-research.

## Declaration of interests

The paper declares no conflict of interest, but Angela Hwang, MS, and Kwaku Poku Asante, MD declare on their ICMJE forms consulting fees and support for attending meetings related to this research.

## Appendix ASupplementary data

Appendix

Survey data
