# Supplementary material for: Identifying WHO global priority endemic pathogens for vaccine research and development (R&D) using multi-criteria decision analysis (MCDA): an objective of the Immunization Agenda 2030
Source: eBioMedicine. 2024 Nov 11;110:105424. doi: 10.1016/j.ebiom.2024.105424 (PMC11663754; doi:10.1016/j.ebiom.2024.105424)
Supplement: Appendix [file mmc1.pdf]

Title

Identifying WHO global priority endemic pathogens for vaccine research and development (R&D) using multi-criteria decision analysis (MCDA): an objective of the Immunization Agenda 2030

Appendix

Contents

Interim reports, including detailed methods 5

Pathogens identified through literature review 6

Survey preparation 7

Pilot testing 7

Pathogen scoring 8

Pathogen scores by region 10

Survey dissemination 17

Dissemination channels 17

Survey Invitation 18

Survey components 23

Survey introduction 23

PAPRIKA survey questions 26

Post-survey information 28

Survey participants 31

Survey results 34

Criteria weights 34

Pathogen ranks by region 35

Use Cases and Action Categories 38

Detailed methods for assessing predictors and cluster patterns in MCDA-generated criteria weights 41

Detailed results from assessing predictors and cluster patterns in MCDA-generated criteria weights 43

References 69

Tables

sTable 1 Pathogens considered for vaccine R&D prioritization 6
sTable 2 Pathogen scoring guide 8
sTable 3 Coding for data availability 10
sTable 4 Pathogen scores, African Region 11
sTable 5 Pathogen scores, Region of the Americas 12
sTable 6 Pathogen scores, Eastern Mediterranean Region 13
sTable 7 Pathogen scores, European Region 14
sTable 8 Pathogen scores, South-East Asian Region 15
sTable 9 Pathogen scores, Western Pacific Region 16
sTable 10 Regional and country representation in included survey responses 31
sTable 11 Characteristics of survey participants 32
sTable 12 Raw mean criteria weights 34
sTable 13 Pathogen total weights and ranks by region 35
sTable 14 Robustness testing results 37
sTable 15 Use cases and Action Categories for pathogens on the Global list 38

Figures

sFigure 1 Pilot testing results 7

sFigure 2 Survey dissemination 17

sFigure 3 Survey invitation: Example email to immunization stakeholders 18

sFigure 4 Survey invitation: Example email attachment 20

sFigure 5 Survey introduction 23

sFigure 6 Example Survey Question 26

sFigure 7 Calculating total weight for a pathogen 27

sFigure 8 Post-survey information (demo survey) 28

sFigure 9 Survey responses 31

sFigure 10 Adjusted mean difference in criteria weights by region, Criteria 1-4 (n=273) 43

sFigure 11 Adjusted mean difference in criteria weights by region, Criteria 5-8 (n=273) 44

sFigure 12 Adjusted mean difference in criteria weights by language of survey, Criteria 1-4 (n=273) 45

sFigure 13 Adjusted mean difference in criteria weights by language of survey, Criteria 5-8 (n=273) 46

sFigure 14 Adjusted mean difference in criteria weights by participant characteristics: Years of experience (n=273) 47

sFigure 15 Adjusted mean difference in criteria weights by participant characteristics: Areas of expertise and Annual deaths in children under 5 years (n=273) 48

sFigure 16 Adjusted mean difference in criteria weights by participant characteristics: Areas of expertise and Annual deaths in people 5 years and older (n=273) 49

sFigure 17 Adjusted mean difference in criteria weights by participant characteristics: Areas of expertise and Years lived with disability (n=273) 50

sFigure 18 Adjusted mean difference in criteria weights by participant characteristics: Areas of expertise and Social and economic burden per case (n=273) 51

sFigure 19 Adjusted mean difference in criteria weights by participant characteristics: Areas of expertise and Disruption due to outbreaks (n=273) 52

sFigure 20 Adjusted mean difference in criteria weights by participant characteristics: Areas of expertise and Contribution to inequity (n=273) 53

sFigure 21 Adjusted mean difference in criteria weights by participant characteristics: Areas of expertise and Contribution to antimicrobial resistance (n=273) ... 54

sFigure 22 Adjusted mean difference in criteria weights by participant characteristics: Areas of expertise and Unmet needs for prevention and treatment (n=273) ... 55

sFigure 23 Adjusted mean difference in criteria weights by participant characteristics: Type of work organisation and Annual deaths in children under 5 years (n=273) ... 56

sFigure 24 Adjusted mean difference in criteria weights by participant characteristics: Type of work organisation and Annual deaths in people 5 years and older (n=273) ... 57

sFigure 25 Adjusted mean difference in criteria weights by participant characteristics: Type of work organisation and Years lived with disability (n=273) ... 58

sFigure 26 Adjusted mean difference in criteria weights by participant characteristics: Type of work organisation and Social and economic burden per case (n=273) ... 59

sFigure 27 Adjusted mean difference in criteria weights by participant characteristics: Type of work organisation and Disruption due to outbreaks (n=273) ... 60

sFigure 28 Adjusted mean difference in criteria weights by participant characteristics: Type of work organisation and Contribution to inequity (n=273) ... 61

sFigure 29 Adjusted mean difference in criteria weights by participant characteristics: Type of work organisation and Contribution to antimicrobial resistance (n=273) ... 62

sFigure 30 Adjusted mean difference in criteria weights by participant characteristics: Type of work organisation and Unmet needs for prevention and treatment (n=273) ... 63

sFigure 31 Adjusted mean difference in criteria weights by participant characteristics: Gavi eligibility of participants' country of work (n=273) ... 64

sFigure 32 Adjusted mean difference in criteria weights by participant characteristics: World Bank classification of participants' country of work (n=273) ... 65

sFigure 33 Criteria weights by cluster (2-cluster model) (n=273) ... 66

sFigure 34 Participant Factors associated with cluster membership, Years of experience and Areas of expertise ... 67

sFigure 35 Participant Factors associated with cluster membership, Type of work organization and Country of work ... 68

Interim reports, including detailed methods

Full descriptions of the method, including scoring process, pathogen scores, and data sources are given in the following reports:

- *Vaccine R&D Priorities: Initial Landscaping and Proposed Methods, for PDVAC input*. July 2022. Available at: https://www.technet-21.org/en/resources/report/vaccine-r-d-priorities-initial-landscaping-and-proposed-methods-for-pdvac-input
- *Vaccine R&D Priorities: Survey Preparation and Launch, for PDVAC input*. November 2022. Available at: https://www.technet-21.org/en/resources/report/vaccine-r-d-priorities-survey-preparation-and-launch-for-pdvac-input-includes-pathogen-scoring
- *Vaccine R&D Priorities: Survey Progress and Preliminary Results, for SAGE input*. February 2023. Available at: https://www.technet-21.org/en/resources/report/vaccine-r-d-priorities-survey-progress-and-preliminary-results-for-sage-input-2
- *Vaccine R&D Priorities: Update on Pathogen Scope and Scoring*. March 2024. Available at: https://www.technet-21.org/en/resources/report/vaccine-r-d-priorities-update-on-pathogen-scope-and-scoring-march-2024
- *Vaccine R&D Priorities: Vaccine Use Cases and Action Categories, for PDVAC input.* December 2023. Available at: https://www.technet-21.org/en/resources/report/vaccine-r-d-priorities-vaccine-use-cases-and-action-categories-for-pdvac-input-december-2024

Pathogens identified through literature review

Potential pathogens for vaccine R&D are shown in sTable 1. In this list, antibiotic resistance was not considered separately: for example, *Neisseria gonorrhoeae* would include cephalosporin and fluoroquinolone-resistant strains as well as susceptible strains. When divergent product profiles apply to a single pathogen, such as seasonal and broadly protective influenza vaccines, or tuberculosis vaccines for adults and adolescents, rather than infants, they were captured separately. Finally, some entries are not pathogens but diseases: these reflect the nomenclature used in the source materials. Several screens were applied to focus the MCDA exercise on the most relevant pathogens. These screens, and the pathogens eliminated by each one, are also shown in sTable 1.

sTable 1 Pathogens considered for vaccine R&D prioritization

Include in MCDA exercise	Exclude, not considered a human pathogen or disease	Exclude, emerging infectious disease addressed by the R&D Blueprint	Exclude, has licensed vaccines for main target product profile(s)	Exclude, no candidate vaccines identified in clinical development	Exclude, vaccine R&D not prioritized by global strategies or advisory mechanisms, not suggested by regional stakeholders
Chikungunya virus<br>*Chlamydia trachomatitis*<br>Cytomegalovirus<br>Dengue virus<br>Extra-intestinal pathogenic *E coli* (ExPEC)<br>Group A streptococcus (*Streptococcus pyogenes,* GAS)<br>Group B streptococcus (*Streptococcus agalactiae,* GBS)<br>Hepatitis C<br>Herpes simplex types 1 and 2<br>Human immunodeficiency virus 1 (HIV-1)<br>Hookworm<br>Influenza virus (broadly protective vaccines)<br>Intestinal pathogenic *E coli* (InPEC)<br>*Klebsiella pneumoniae*<br>*Leishmania* species<br>*Mycobacterium leprae*<br>*Mycobacterium tuberculosis* (beyond infancy)<br>*Neisseria gonorrhoeae*<br>Norovirus<br>*Plasmodium falciparum*<br>Respiratory syncytial virus (RSV)<br>*Salmonella* (non-typhoidal, NTS)<br>*Salmonella* Paratyphi<br>Schistosomes<br>*Shigella* species<br>*Staphylococcus aureus*	Bovine coronavirus<br>Bovine respiratory disease<br>Brucellosis<br>Chronic wasting disease<br>Coccidiosis<br>Contagious Bovine Pleuropneumonia (CBPP)<br>*E coli* (cattle infections)<br>Echinococcosis (type not specified)<br>Foot-and-mouth disease virus<br>Gallid alpha herpesvirus 2<br>Peste des petits ruminants<br>Porcine epidemic diarrhea virus<br>Porcine influenza A<br>*Staphylococcus aureus* (dairy cattle)<br>*Theileria parva*<br>Tick infestation (animals)	Crimean-Congo haemorrhagic fever<br>Lassa fever virus<br>Marburg virus<br>MERS-CoV<br>Nipah virus<br>Rift Valley Fever virus<br>SARS-CoV-1<br>SARS-CoV-2 (broadly protective)<br>Zika virus	Adenovirus<br>*Bacillus anthracis*<br>*Bordetella pertussis*<br>*Clostridium tetani*<br>*Corynebacterium diphtheriae*<br>*Coxiella burnetii* (Q fever)<br>Dengue virus (for dengue-immune individuals)<br>Ebola virus<br>Enterovirus 71<br>*Haemophilus influenzae* type B<br>Hepatitis A and B<br>Human papillomavirus<br>Influenza virus (avian, pandemic, and seasonal)<br>Japanese encephalitis<br>Junin virus<br>Measles virus<br>Mumps virus<br>*Mycobacterium tuberculosis* (BCG for infants)<br>*Neisseria meningitidis* serogroups A, B, C, W, X, Y<br>Polio virus<br>Rabies virus<br>Rotavirus<br>Rubella virus<br>*Salmonella* Typhi<br>SARS-CoV-2<br>Smallpox virus<br>*Streptococcus pneumoniae*<br>Tick-borne encephalitis virus<br>Varicella zoster virus<br>*Vibrio cholerae*<br>Yellow fever virus	*Acinetobacter baumannii*<br>*Ascaris lumbricoides*<br>Aspergilllus<br>*Burkholderia pseudomallei*<br>*Cryptococcus* species<br>Cryptosporidium<br>*Dracunculus medinensis*<br>*Echinococcus granulosus*<br>*Echinococcus multilocularis*<br>Ehrlichiosis<br>*Enterococcus faecium*<br>*Haemophilus influenzae* type A<br>*Helicobacter pylori*<br>Hepatitis D virus<br>Human T-lymphotropic virus type 1<br>Lymphatic filariasis<br>Mycetoma<br>*Mycobacterium ulcerans*<br>*Onchocerca volvulus*<br>*Pseudomonas aeruginosa*<br>*Sarcoptes scabiei*<br>*Streptococcus mutans*<br>*Strongyloides stercoralis*<br>*Toxoplasma gondii*<br>*Treponema pallidum*<br>*Treponema pallidum subspecies pertenue*<br>*Trichomonas vaginalis*<br>*Trichuris trichiura*<br>*Trypanosoma brucei*<br>*Trypanosoma cruzi*	*Borrelia burgdorferi*<br>Campylobacter<br>*Candida* species<br>*Clostridium botulinum*<br>*Clostridium difficile*<br>Coccidioides (Valley fever/coccidioidomycosis)<br>Coxsackievirus Group B<br>Epstein-Barr virus<br>Equine encephalitis virus (Eastern, Venezuelan, and Western)<br>*Francisella tularensis*<br>*Haemophilus influenzae* non-type B<br>Hanta viruses (including Hantaan and Puumala)<br>Henipavirus<br>Hepatitis E virus<br>Human metapneumovirus<br>Human parainfluenza virus types 1, 2 and 3<br>Leptospirosis<br>*Listeria monocytogenes*<br>Monkeypox virus<br>*Mycobacterium avium* subspecies paratuberculosis<br>Parvovirus<br>*Plasmodium vivax*<br>Ross River Virus<br>*Taenia solium*<br>West Nile Virus<br>Yersinia pestis

Survey preparation

Pilot testing

A pilot version of the survey was developed in July 2022 as described on page 32 of *Vaccine R&D Priorities: Initial Landscaping and Proposed Methods, for PDVAC input*. (Available at: https://www.technet-21.org/en/resources/report/vaccine-r-d-priorities-initial-landscaping-and-proposed-methods-for-pdvac-input)

The pilot survey was shared with PDVAC members in advance of the 18 July 2022 PDVAC meeting. Results of the pilot are shown in sFigure 1. Participant feedback shared during the meeting was incorporated in the final surveys.

sFigure 1 Pilot testing results

36 individuals were invited to participate in pilot and 28 of them (78%) opened the link to the pilot survey.

Number of responses

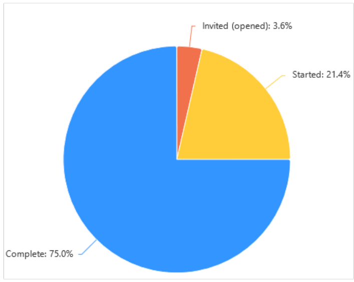

- **75% (21/28)** of responses were completed
- **21% (6/28)** of responses were started but not completed
- **3.6% (1/28)** of responses opened but did not start the survey

Time taken to complete survey

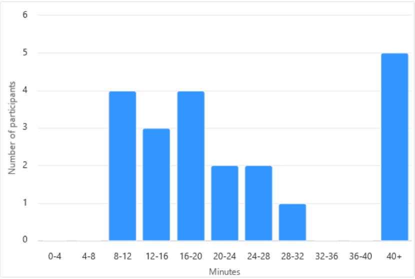

- Median time taken to complete the pilot survey was **19 minutes** (interquartile range: 13-32 minutes)

Number of trade-offs

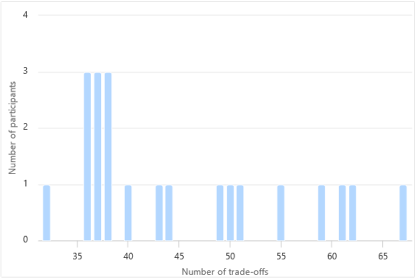

- Median number of trade-offs was **40** (interquartile range: 37-51)

Participant feedback

Explanation
Was the purpose of the exercise sufficiently explained?

Yes, well explained	14	77.8%
The explanations should be improved	4	22.2%
The exercise wasn't sufficiently explained at all	0	0.0%
Not answered	3	14.3%

Description of criteria
Were the descriptions of criteria and their values clearly described?

Yes, well described	14	77.8%
The descriptions should be improved	4	22.2%
The descriptions weren't clear at all	0	0.0%
Not answered	3	14.3%

Overall, how did you find the exercise (please tick all that apply)?

Interesting	13	68.4%
Challenging	10	52.6%
Confusing	4	21.1%
Fun	2	10.5%
Time-consuming	4	21.1%
Other positive	0	0.0%
Other negative	0	0.0%
Not answered	2	9.5%

Overall, do you think that this approach could be scaled-up with a goal to prioritise pathogens for vaccine development?

Absolutely	6	31.6%
Yes but with modifications	12	63.2%
No	1	5.3%
Not answered	2	9.5%

Pathogen scoring

Pathogens were scored as described in the Methods section using a scoring guide (sTable 2).

sTable 2 Pathogen scoring guide

Thresholds for quantitative criteria (1-3) were set based on the highest burden in each region caused by a pathogen in the scope of this exercise (As recommended by PDVAC, HIV-1, *M tuberculosis*, and *P falciparum* were excluded from threshold-setting to improve discrimination at the lower levels).

**Criteria /** ***Sub-criteria***	**Very low**	**Low**	**Medium**	**High**	**Very high**
1 Annual deaths in children under 5	Less than 20% of highest regional burden	20% - 40% of highest regional burden	40% - 60% of highest regional burden	60% - 80% of highest regional burden	Greater than 80% of highest regional burden
2 Annual deaths in people older than 5	Less than 20% of highest regional burden	20% - 40% of highest regional burden	40% - 60% of highest regional burden	60% - 80% of highest regional burden	Greater than 80% of highest regional burden
3 Years lived with disability (all ages)	Less than 20% of highest regional burden	20% - 40% of highest regional burden	40% - 60% of highest regional burden	60% - 80% of highest regional burden	Greater than 80% of highest regional burden
4 Social and economic burden per case	Very low burden on families and societies	Low burden on families and societies	Moderate burden on families and societies	High burden on families and societies	Very high burden on families and societies
4.1 Economic burden to families	*Rarely leads to hospitalization or very low cost of treatment*<br>*Little or no losses of productivity*	*Seldom requires hospitalization or low cost of treatment*<br>*Minor losses of productivity*	*Sometimes requires hospitalization or moderate cost of treatment*<br>*Some losses of productivity*	*Often requires hospitalization or high cost of treatment*<br>*Moderate losses of productivity*	*Typically requires hospitalization or very high cost of treatment*<br>*Serious losses of productivity*
4.2 Social burden to families	*Little or no impact on education or social well-being (e.g. due to stigma)*	*Minor impact on education or social well-being (e.g. due to stigma)*	*Some impact on education or social well-being (e.g. due to stigma)*	*Moderate impact on education or well-being (e.g. due to stigma)*	*Serious impact on education or social well-being (e.g. due to stigma)*
5 Disruption due to outbreaks and epidemics	Little or no social disruption or impact on healthcare, trade or tourism	Slight social disruption or impact on healthcare, trade or tourism	Moderate social disruption or impact on healthcare, trade or tourism	High social disruption or impact on healthcare, trade or tourism, including due to preventive measures	Very high social disruption or impact on healthcare, trade and tourism, including due to preventive measures
6 Contribution to inequity	Affects all communities equally	Burden falls on socially and economically disadvantaged groups, including women, slightly more than other groups	Burden falls on socially and economically disadvantaged groups, including women, moderately more than other groups	Burden falls on socially and economically disadvantaged groups, including women, much more than other groups	Burden falls on socially and economically disadvantaged groups, including women, all or most of the time

**Criteria / *Sub-criteria***	**Very low**	**Low**	**Medium**	**High**	**Very high**
7 Contribution to antimicrobial resistance (AMR)	Not resistant to first-line drugs and not associated with antimicrobial use	Little resistance to first-line drugs and little association with antimicrobial use	Some resistance to first-line drugs, associated with high antimicrobial use	Significant resistance to first-line drugs, associated with high antimicrobial use	A global resistance threat due to widespread resistance and association with high antimicrobial use
7.1 AMR Priority	*The pathogen has not been highlighted as a priority for AMR*	*The pathogen has not been highlighted as a priority for AMR*	*The pathogen has been highlighted as a country priority for AMR*	*The pathogen has been highlighted as a regional priority for AMR*	*The pathogen has been highlighted as a high or critical global priority for AMR*
7.2 Frequency of resistance	*Very few isolates are resistant to first-line antimicrobial drugs*	*A low proportion of isolates is resistant to first-line antimicrobial drugs*	*A moderate proportion of isolates is resistant to first-line antimicrobial drugs*	*A high proportion of isolates is resistant to first-line antimicrobial drugs*	*A high proportion of global isolates is resistant to first-line antimicrobial drugs*
7.3 Antibiotic use	*Low antimicrobial use is associated with infection by the pathogen*	*Moderate or low antimicrobial use is associated with infection by the pathogen*	*High antimicrobial use is associated with infection by the pathogen*	*High antimicrobial use is associated with infection by the pathogen*	*High antimicrobial use is associated with infection by the pathogen*
8 Unmet needs for prevention and treatment	The alternatives for prevention or treatment meet the needs of most people	The alternatives for prevention or treatment meet the needs of many people	The alternatives for prevention or treatment meet the needs of some people	The alternatives for prevention or treatment meet the needs of few people	There are no effective alternatives for prevention or treatment

Pathogen scores by region

Pathogens were scored in the context of each region as described in the Methods section. For transparency, scores were coded for data availability as shown in sTable 3. Scores as of April 2024 are shown in sTable 4 to sTable 9.

sTable 3 Coding for data availability

	Criteria	Coding
Quantitative scoring	*Annual deaths in children under 5*<br>*Annual deaths in people 5 and older*<br>*Annual years lived with disability (all ages)*	A. Scored using burden data from GBD 2019<br>B. Scored using burden data from other studies<br>C. Systematic data not found, scored as discussed in *Vaccine R&D Priorities: Survey Preparation and Launch, for PDVAC input*. November 2022. Available at: https://www.technet-21.org/en/resources/report/vaccine-r-d-priorities-survey-preparation-and-launch-for-pdvac-input-includes-pathogen-scoring
Qualitative scoring	*Social and economic burden per case*<br>*Disruption due to outbreaks*<br>*Contribution to inequity*<br>*Contribution to antimicrobial resistance*<br>*Unmet needs for prevention & treatment*	A. Scored based on data from regional sources<br>B. Score inferred based on data for other regions or other pathogens in the same region

sTable 4 Pathogen scores, African Region

A, B, and C codes refer to the level of data available for scoring, as shown in sTable 3.

	1 Annual deaths in children under 5	2 Annual deaths in people 5 and older	3 Annual years lived with disability (all ages)	4 Social and economic burden per case	5 Disruption due to outbreaks	6 Contribution to inequity	7 Contribution to antimicrobial resistance	8 Unmet needs for prevention & treatment
Chikungunya virus	Very low (B)	Very low (B)	Very low (B)	Medium (A)	Medium (A)	Medium (A)	Very Low (A)	Very high (A)
Chlamydia trachomatis	Very low (A)	Very low (A)	Very low (A)	Very high (A)	Very low (B)	High (A)	Low (A)	High (A)
Cytomegalovirus	Very low (C)	Low (C)	Medium (C)	High (B)	Very low (B)	Medium (A)	Very low (B)	Very high (B)
Dengue virus	Very low (A)	Very low (A)	Very low (A)	Medium (B)	Medium (A)	Medium (B)	Medium (B)	High (A)
Extra-intestinal pathogenic *E coli*	Medium (A)	High (A)	Very low (A)	Medium (B)	Low (B)	Medium (A)	Very high (A)	Medium (A)
Group A streptococcus	Very low (A)	Low (A)	Medium (A)	High (A)	Very low (B)	High (B)	High (B)	High (A)
Group B streptococcus	High (A)	Low (A)	Very low (A)	High (A)	Low (B)	Medium (A)	Low (A)	Very high (A)
Hepatitis C virus	Very low (A)	Medium (A)	Very low (A)	Very high (A)	Low (B)	Very high (A)	Low (B)	High (A)
Herpes simplex types 1 and 2	Very low (C)	Very low (C)	Very low (A)	Very high (A)	Very low (B)	Very high (A)	Low (A)	High (A)
Hookworm	Very low (C)	Very low (C)	Medium (A)	Low (A)	Very low (B)	Very high (A)	Low (A)	Low (A)
HIV-1	Low (A)	Very high (A)	Very high (A)	Very high (A)	High (A)	Very high (A)	Very high (A)	High (A)
Influenza	Very low (A)	Low (A)	Very low (A)	Low (B)	Very high (A)	Medium (B)	Medium (B)	High (A)
Intestinal pathogenic *E coli*	Very low (A)	Very low (A)	Very low (A)	Medium (A)	Medium (B)	Medium (A)	Very high (A)	Medium (A)
Klebsiella pneumoniae	Very high (A)	Very high (A)	Very low (A)	High (B)	Low (A)	Low (B)	Very high (A)	High (A)
Leishmania	Very low (A)	Very low (A)	Very low (A)	Very high (B)	High (A)	Very high (A)	Medium (A)	Medium (A)
Mycobacterium leprae (leprosy)	Very low (C)	Very low (C)	Very low (A)	Very high (A)	Very low (A)	Very high (B)	Low (A)	High (A)
Mycobacterium tuberculosis	Low (B)	Very high (B)	Very high (A)	Very high (A)	Very high (A)	Very high (A)	Very high (A)	High (A)
Neisseria gonorrhoeae	Very low (A)	Very low (A)	Very low (A)	Medium (B)	Low (B)	High (A)	Very high (A)	Medium (A)
Non-typhoidal *Salmonella*	High (A)	Low (A)	Very low (A)	High (A)	Medium (B)	Very high (A)	High (A)	High (A)
Norovirus	Low (A)	Low (A)	Very low (A)	Medium (A)	High (A)	Medium (A)	Low (B)	High (A)
Plasmodium falciparum (malaria)	Very high (C)	Very high (C)	Very high (C)	High (A)	High (A)	Very high (A)	High (A)	High (A)
Respiratory syncytial virus	High (A)	Low (A)	Very low (A)	Medium (B)	High (A)	Medium (B)	High (B)	Medium (A)
Salmonella Paratyphi	Very low (A)	Very low (A)	Very low (A)	Low (B)	Low (A)	High (B)	Low (B)	Medium (B)
Schistosomes	Very low (A)	Very low (A)	Very high (A)	Medium (A)	Low (A)	Very high (A)	Low (A)	High (A)
Shigella species	High (A)	Low (A)	Low (A)	High (A)	Medium (A)	High (B)	High (B)	High (A)
Staphylococcus aureus	High (A)	Very high (A)	Very low (A)	High (B)	Low (B)	Medium (B)	Very high (A)	High (A)

sTable 5 Pathogen scores, Region of the Americas

A, B, and C codes refer to the level of data available for scoring, as shown in sTable 3.

	1 Annual deaths in children under 5	2 Annual deaths in people 5 and older	3 Annual years lived with disability (all ages)	4 Social and economic burden per case	5 Disruption due to outbreaks	6 Contribution to inequity	7 Contribution to antimicrobial resistance	8 Unmet needs for prevention & treatment
Chikungunya virus	Very low (B)	Very low (B)	Low (B)	Medium (A)	High (A)	Medium (A)	Very low (B)	Very high (A)
Chlamydia trachomatis	Very low (A)	Very low (A)	Very low (A)	Very high (A)	Very low (A)	High (A)	Low (A)	High (A)
Cytomegalovirus	Low (C)	Low (C)	Medium (C)	High (A)	Very low (B)	Medium (A)	Very low (B)	Very high (B)
Dengue virus	Very low (A)	Very low (A)	Very low (A)	Medium (A)	Very high (A)	Medium (A)	Medium (B)	High (A)
Extra-intestinal pathogenic *E coli*	High (A)	High (A)	Very low (A)	Medium (A)	Low (A)	Medium (A)	Very high (A)	Medium (A)
Group A streptococcus	Low (A)	Low (A)	Very high (A)	Medium (A)	Very low (A)	Medium (A)	High (B)	Medium (A)
Group B streptococcus	Medium (A)	Very low (A)	Very low (A)	High (A)	Low (A)	Medium (A)	Low (A)	High (A)
Hepatitis C virus	Very low (A)	Medium (A)	Very low (A)	Very high (A)	Low (A)	Very high (A)	Low (A)	High (A)
Herpes simplex types 1 and 2	Low (C)	Very low (C)	Very low (A)	High (A)	Very low (B)	High (A)	Low (A)	High (A)
Hookworm	Very low (C)	Very low (C)	Very low (A)	Low (A)	Very low (B)	Very high (A)	Low (B)	Low (A)
HIV-1	Low (A)	Low (A)	Very high (A)	Very high (A)	High (A)	Very high (A)	Very high (A)	High (A)
Influenza	Low (A)	Low (A)	Very low (A)	Low (A)	Very high (A)	Medium (A)	Medium (A)	High (A)
Intestinal pathogenic *E coli*	Very low (A)	Very low (A)	Very low (A)	Medium (A)	Medium (B)	Medium (B)	Very high (A)	Medium (A)
Klebsiella pneumoniae	Very high (A)	Medium (A)	Very low (A)	High (A)	Low (A)	Low (B)	Very high (A)	High (A)
Leishmania	Very low (A)	Very low (A)	Very low (A)	Very high (A)	Medium (A)	Very high (A)	Medium (A)	Medium (A)
Mycobacterium leprae (leprosy)	Very low (C)	Very low (C)	Very low (A)	Very high (A)	Very low (A)	Very high (A)	Medium (A)	High (A)
Mycobacterium tuberculosis	Very low (B)	Very low (B)	Low (A)	Very high (A)	High (A)	Very high (A)	Very high (A)	High (A)
Neisseria gonorrhoeae	Very low (A)	Very low (A)	Very low (A)	Medium (A)	Low (A)	High (A)	Very high (A)	Medium (A)
Non-typhoidal *Salmonella*	Very low (A)	Very low (A)	Very low (A)	Low (B)	Low (A)	High (B)	High (A)	Medium (A)
Norovirus	Very low (A)	Very low (A)	Low (A)	Medium (A)	High (A)	Medium (A)	Low (B)	High (A)
Plasmodium falciparum (malaria)	Very low (C)	Very low (C)	Very low (C)	High (A)	Medium (A)	Very high (A)	High (A)	Medium (A)
Respiratory syncytial virus	Medium (A)	Low (A)	Very low (A)	Medium (A)	High (A)	Medium (A)	Medium (A)	Medium (A)
Salmonella Paratyphi	Very low (A)	Very low (A)	Very low (A)	Low (B)	Low (A)	High (B)	Low (B)	Low (B)
Schistosomes	Very low (A)	Very low (A)	Low (A)	Low (A)	Low (A)	High (A)	Low (B)	Medium (A)
Shigella species	Very low (A)	Very low (A)	Low (A)	High (A)	Medium (A)	High (A)	High (A)	High (A)
Staphylococcus aureus	Very high (A)	Very high (A)	Very low (A)	High (A)	Low (A)	Medium (B)	Very high (A)	High (A)

sTable 6 Pathogen scores, Eastern Mediterranean Region

A, B, and C codes refer to the level of data available for scoring, as shown in sTable 3.

	1 Annual deaths in children under 5	2 Annual deaths in people 5 and older	3 Annual years lived with disability (all ages)	4 Social and economic burden per case	5 Disruption due to outbreaks	6 Contribution to inequity	7 Contribution to antimicrobial resistance	8 Unmet needs for prevention & treatment
Chikungunya virus	Very low (B)	Very low (B)	Very low (B)	Medium (A)	Low (A)	Medium (A)	Very low (B)	Very high (A)
Chlamydia trachomatis	Very low (A)	Very low (A)	Very low (A)	Very high (A)	Very low (B)	High (A)	Low (A)	High (A)
Cytomegalovirus	Very low (C)	Low (C)	Medium (C)	High (B)	Very low (B)	Medium (A)	Very low (B)	Very high (B)
Dengue virus	Very low (A)	Very low (A)	Very low (A)	Medium (A)	Medium (A)	Medium (A)	Medium (B)	High (A)
Extra-intestinal pathogenic *E coli*	High (A)	High (A)	Very low (A)	Medium (A)	Low (B)	Medium (A)	Very high (A)	Medium (A)
Group A streptococcus	Very low (A)	Medium (A)	Very high (A)	Medium (A)	Very low (B)	Medium (B)	High (B)	Medium (A)
Group B streptococcus	High (A)	Very low (A)	Very low (A)	High (B)	Low (B)	Medium (B)	Very low (B)	High (A)
Hepatitis C virus	Very low (A)	Very high (A)	Very low (A)	Very high (A)	Low (B)	Very high (A)	Low (B)	High (A)
Herpes simplex types 1 and 2	Very low (C)	Very low (C)	Very low (A)	Medium (B)	Very low (B)	Medium (A)	Low (A)	High (A)
Hookworm	Very low (C)	Very low (C)	Low (A)	Low (B)	Very low (B)	Very high (A)	Low (B)	Low (A)
HIV-1	Very low (A)	Low (A)	Very low (A)	Very high (A)	High (A)	Very high (A)	Very high (A)	High (A)
Influenza	Very low (A)	Very low (A)	Very low (A)	Low (B)	Very high (A)	Medium (B)	Medium (B)	High (A)
Intestinal pathogenic *E coli*	Very low (A)	Very low (A)	Very low (A)	Medium (A)	Medium (B)	Medium (B)	Very high (A)	Medium (A)
Klebsiella pneumoniae	Very high (A)	Medium (A)	Very low (A)	High (B)	Low (A)	Low (B)	Very high (A)	High (A)
Leishmania	Very low (A)	Very low (A)	Very high (A)	Very high (A)	High (A)	Very high (A)	Medium (A)	Medium (A)
Mycobacterium leprae (leprosy)	Very low (C)	Very low (C)	Very low (A)	Very high (B)	Very low (A)	Very high (B)	Low (A)	High (A)
Mycobacterium tuberculosis	Low (B)	Very high (B)	Very high (A)	Very high (A)	High (A)	Very high (A)	Very high (A)	High (A)
Neisseria gonorrhoeae	Very low (A)	Very low (A)	Very low (A)	Medium (B)	Low (B)	High (B)	Very high (A)	Medium (A)
Non-typhoidal *Salmonella*	Very low (A)	Very low (A)	Very low (A)	Low (B)	Low (B)	High (B)	High (B)	Medium (A)
Norovirus	Low (A)	Very low (A)	Low (A)	Medium (A)	High (A)	Medium (A)	Low (B)	High (A)
Plasmodium falciparum (malaria)	Very low (C)	Very low (C)	Low (C)	High (A)	Low (A)	High (B)	Medium (A)	Medium (A)
Respiratory syncytial virus	Low (A)	Very low (A)	Very low (A)	Medium (A)	High (A)	Medium (B)	Low (B)	Medium (A)
Salmonella Paratyphi	Very low (A)	Very low (A)	Very low (A)	Low (A)	Low (A)	High (B)	High (A)	Medium (A)
Schistosomes	Very low (A)	Very low (A)	Low (A)	Low (A)	Low (B)	High (A)	Low (A)	Medium (A)
Shigella species	Low (A)	Very low (A)	Medium (A)	Medium (A)	Medium (B)	High (B)	High (B)	High (A)
Staphylococcus aureus	High (A)	High (A)	Very low (A)	High (B)	Low (A)	Medium (B)	Very high (A)	High (A)

sTable 7 Pathogen scores, European Region

A, B, and C codes refer to the level of data available for scoring, as shown in sTable 3.

	1 Annual deaths in children under 5	2 Annual deaths in people 5 and older	3 Annual years lived with disability (all ages)	4 Social and economic burden per case	5 Disruption due to outbreaks	6 Contribution to inequity	7 Contribution to antimicrobial resistance	8 Unmet needs for prevention & treatment
Chikungunya virus	Very low (B)	Very low (B)	Very low (B)	Medium (A)	Medium (A)	Medium (A)	Very low (B)	Very high (A)
Chlamydia trachomatis	Very low (A)	Very low (A)	Very low (A)	Very high (A)	Very low (A)	High (A)	Low (A)	High (A)
Cytomegalovirus	Medium (C)	Low (C)	Medium (C)	High (A)	Very low (B)	Medium (A)	Very low (B)	Very high (A)
Dengue virus	Very low (A)	Very low (A)	Very low (A)	Very low (B)	Low (A)	Medium (B)	Very low (B)	Medium (A)
Extra-intestinal pathogenic *E coli*	Medium (A)	Very high (A)	Low (A)	Medium (A)	Low (B)	Medium (A)	Very high (A)	Medium (A)
Group A streptococcus	Low (A)	Low (A)	Very high (A)	Medium (A)	Very low (A)	Medium (B)	High (B)	Medium (A)
Group B streptococcus	Low (A)	Very low (A)	Very low (A)	High (A)	Low (B)	Medium (A)	Low (A)	High (A)
Hepatitis C virus	Very low (A)	Low (A)	Low (A)	Very high (A)	Very low (A)	Very high (A)	Low (A)	High (A)
Herpes simplex types 1 and 2	Very low (C)	Very low (C)	Low (A)	Medium (A)	Very low (B)	Medium (A)	Low (A)	High (A)
Hookworm	Very low (C)	Very low (C)	Very low (A)	Low (B)	Very low (B)	Very high (A)	Low (B)	Low (A)
HIV-1	Very low (A)	Very low (A)	Very high (A)	Very high (A)	High (A)	Very high (A)	Very high (A)	High (A)
Influenza	Low (A)	Very low (A)	Very low (A)	Low (A)	Very high (A)	Medium (B)	Medium (B)	High (A)
Intestinal pathogenic *E coli*	Very low (A)	Very low (A)	Very low (A)	Medium (A)	Medium (B)	Medium (B)	Very high (A)	Medium (A)
Klebsiella pneumoniae	High (A)	Medium (A)	Very low (A)	High (B)	Low (A)	Low (B)	Very high (A)	High (A)
Leishmania	Very low (A)	Very low (A)	Very low (A)	Very high (A)	Very low (B)	High (A)	Very low (A)	Medium (A)
Mycobacterium leprae (leprosy)	Very low (C)	Very low (C)	Very low (A)	Very high (A)	Very low (A)	Very high (A)	Very low (B)	High (A)
Mycobacterium tuberculosis	Low (B)	Very low (B)	Very high (A)	Very high (A)	High (A)	Very high (A)	Very high (A)	High (A)
Neisseria gonorrhoeae	Very low (A)	Very low (A)	Very low (A)	Medium (B)	Low (B)	High (A)	Very high (A)	Medium (A)
Non-typhoidal *Salmonella*	Very low (A)	Very low (A)	Very low (A)	Low (B)	Low (B)	High (B)	High (B)	Medium (A)
Norovirus	Very low (A)	Very low (A)	High (A)	Low (A)	High (A)	Medium (A)	Low (B)	High (A)
Plasmodium falciparum (malaria)	Very low (C)	Very low (C)	Very low (C)	Low (B)	Very low (A)	Very Low (B)	Very low (A)	Very low (B)
Respiratory syncytial virus	Very high (A)	Very low (A)	Very low (A)	Medium (A)	High (A)	Medium (B)	Medium (A)	Medium (A)
Salmonella Paratyphi	Very low (A)	Very low (A)	Very low (A)	Low (B)	Low (A)	High (B)	Low (B)	Low (B)
Schistosomes	Very low (A)	Very low (A)	Very low (A)	Very low (B)	Low (A)	Very low (B)	Very low (B)	Very low (A)
Shigella species	Very low (A)	Very low (A)	Low (A)	Medium (A)	Medium (A)	High (B)	High (B)	High (A)
Staphylococcus aureus	Very high (A)	Very high (A)	Very low (A)	High (A)	Low (A)	Medium (B)	Very high (A)	High (A)

sTable 8 Pathogen scores, South-East Asian Region

A, B, and C codes refer to the level of data available for scoring, as shown in sTable 3.

	1 Annual deaths in children under 5	2 Annual deaths in people 5 and older	3 Annual years lived with disability (all ages)	4 Social and economic burden per case	5 Disruption due to outbreaks	6 Contribution to inequity	7 Contribution to antimicrobial resistance	8 Unmet needs for prevention & treatment
Chikungunya virus	Very low (B)	Very low (B)	Very low (B)	Medium (A)	High (A)	Medium (A)	Very low (B)	Very high (A)
Chlamydia trachomatis	Very low (A)	Very low (A)	Very low (A)	Very high (A)	Very low (B)	High (A)	Low (A)	High (A)
Cytomegalovirus	Very low (C)	Low (C)	Medium (C)	High (B)	Very low (B)	Medium (A)	Very low (B)	Very high (B)
Dengue virus	Very low (A)	Very low (A)	Medium (A)	Medium (A)	Very high (A)	Medium (A)	Medium (A)	High (A)
Extra-intestinal pathogenic *E coli*	High (A)	Very high (A)	Very low (A)	Medium (B)	Low (B)	Medium (A)	Very high (A)	Medium (A)
Group A streptococcus	Very low (A)	Very high (A)	Very high (A)	High (A)	Very low (A)	High (B)	High (B)	High (A)
Group B streptococcus	High (A)	Low (A)	Very low (A)	High (B)	Low (B)	Medium (B)	Very low (A)	Very high (A)
Hepatitis C virus	Very low (A)	High (A)	Very low (A)	Very high (A)	Low (B)	Very high (A)	Low (A)	High (A)
Herpes simplex types 1 and 2	Very low (C)	Very low (C)	Very low (A)	High (A)	Very low (B)	High (A)	Low (A)	High (A)
Hookworm	Very low (C)	Very low (C)	Low (A)	Low (A)	Very low (B)	Very high (A)	Low (B)	Low (A)
HIV-1	Very low (A)	Low (A)	High (A)	Very high (A)	High (A)	Very high (A)	Very high (A)	High (A)
Influenza	Very low (A)	Low (A)	Very low (A)	Low (A)	Very high (A)	Medium (B)	High (B)	High (A)
Intestinal pathogenic *E coli*	Very low (A)	Very low (A)	Very low (A)	Medium (A)	Medium (B)	Medium (B)	Very high (A)	Medium (A)
Klebsiella pneumoniae	Very high (A)	Very high (A)	Very low (A)	High (A)	Low (A)	Low (B)	Very high (A)	High (A)
Leishmania	Very low (A)	Very low (A)	Very low (A)	Very high (A)	High (A)	Very high (A)	Medium (A)	Medium (A)
Mycobacterium leprae (leprosy)	Very low (C)	Very low (C)	Very low (A)	Very high (A)	Very low (A)	Very high (A)	Medium (A)	High (A)
Mycobacterium tuberculosis	Very high (B)	Very high (B)	Very high (A)	Very high (A)	Very high (A)	Very high (A)	Very high (A)	High (A)
Neisseria gonorrhoeae	Very low (A)	Very low (A)	Very low (A)	Medium (B)	Low (B)	High (A)	Very high (A)	Medium (A)
Non-typhoidal *Salmonella*	Very low (A)	Very low (A)	Very low (A)	Low (B)	Very low (A)	High (B)	High (B)	Medium (A)
Norovirus	Very low (A)	Low (A)	Very low (A)	Medium (A)	High (A)	Medium (A)	Low (B)	High (A)
Plasmodium falciparum (malaria)	Low (C)	Very low (C)	Low (C)	High (A)	Medium (A)	High (B)	High (A)	Medium (A)
Respiratory syncytial virus	High (A)	Low (A)	Very low (A)	Medium (A)	High (A)	Medium (B)	High (B)	Medium (A)
Salmonella Paratyphi	Very low (A)	Very low (A)	Very low (A)	Low (A)	Low (A)	High (A)	High (A)	Medium (A)
Schistosomes	Very low (A)	Very low (A)	Very low (A)	Low (B)	Low (B)	High (A)	Low (B)	Medium (A)
Shigella species	Very low (A)	Very low (A)	Low (A)	High (A)	Medium (A)	High (B)	Very high (A)	High (A)
Staphylococcus aureus	High (A)	Very high (A)	Very low (A)	High (B)	Very low (A)	Medium (B)	Very high (A)	High (A)

sTable 9 Pathogen scores, Western Pacific Region

A, B, and C codes refer to the level of data available for scoring, as shown in sTable 3.

	1 Annual deaths in children under 5	2 Annual deaths in people 5 and older	3 Annual years lived with disability (all ages)	4 Social and economic burden per case	5 Disruption due to outbreaks	6 Contribution to inequity	7 Contribution to antimicrobial resistance	8 Unmet needs for prevention & treatment
Chikungunya virus	Very low (B)	Very low (B)	Very low (B)	Medium (A)	High (A)	Medium (A)	Very low (B)	High (A)
Chlamydia trachomatis	Very low (A)	Very low (A)	Very low (A)	Medium (A)	Very low (B)	Medium (A)	Low (A)	High (A)
Cytomegalovirus	Medium (C)	Low (C)	Medium (C)	High (A)	Very low (B)	Medium (A)	Very low (B)	Very high (B)
Dengue virus	Very low (A)	Very low (A)	Low (A)	Medium (A)	Very high (A)	Medium (A)	Medium (B)	High (A)
Extra-intestinal pathogenic *E coli*	High (A)	Medium (A)	Very low (A)	Medium (B)	Low (A)	Medium (A)	Very high (A)	Medium (A)
Group A streptococcus	Low (A)	Medium (A)	Very high (A)	High (A)	Very low (A)	High (A)	High (B)	High (A)
Group B streptococcus	High (A)	Very low (A)	Very low (A)	High (B)	Low (B)	Medium (B)	Low (A)	High (A)
Hepatitis C virus	Very low (A)	Medium (A)	Very low (A)	Very high (A)	Low (A)	Very high (A)	Low (A)	High (A)
Herpes simplex types 1 and 2	Low (C)	Very low (C)	Very low (A)	High (A)	Very low (B)	High (A)	Low (A)	High (A)
Hookworm	Very low (C)	Very low (C)	Low (A)	Low (A)	Very low (B)	Very high (A)	Low (B)	Low (A)
HIV-1	Low (A)	Very low (A)	Medium (A)	Very high (A)	High (A)	Very high (A)	Very high (A)	High (A)
Influenza	Medium (A)	Low (A)	Very low (A)	Low (A)	Very high (A)	Medium (B)	High (B)	High (A)
Intestinal pathogenic *E coli*	Very low (A)	Very low (A)	Very low (A)	Medium (A)	Medium (B)	Medium (B)	Very high (A)	Medium (A)
Klebsiella pneumoniae	Very high (A)	Medium (A)	Very low (A)	High (A)	Low (A)	Low (B)	Very high (A)	High (A)
Leishmania	Very low (B)	Very low (B)	Very low (A)	Very high (A)	Very low (B)	High (A)	Very low (A)	Medium (A)
Mycobacterium leprae (leprosy)	Very low (C)	Very low (C)	Very low (A)	Very high (A)	Very low (A)	Very high (B)	Medium (A)	High (A)
Mycobacterium tuberculosis	Very high (B)	Medium (B)	Very high (A)	Very high (A)	High (A)	Very high (A)	Very high (A)	High (A)
Neisseria gonorrhoeae	Very low (A)	Very low (A)	Very low (A)	Medium (B)	Low (A)	High (A)	Very high (A)	Medium (A)
Non-typhoidal *Salmonella*	Very low (A)	Very low (A)	Very low (A)	Low (B)	Low (B)	High (B)	High (B)	Medium (A)
Norovirus	Very low (A)	Very low (A)	Low (A)	Medium (A)	High (A)	Medium (A)	Low (B)	High (A)
Plasmodium falciparum (malaria)	Very low (C)	Very low (C)	Very low (C)	High (A)	Low (A)	High (B)	Medium (A)	Medium (A)
Respiratory syncytial virus	Very high (A)	Very low (A)	Very low (A)	Medium (A)	High (A)	Medium (B)	High (A)	Medium (A)
Salmonella Paratyphi	Very low (A)	Very low (A)	Very low (A)	Low (B)	Low (A)	High (A)	Medium (A)	Low (B)
Schistosomes	Very low (A)	Very low (A)	Very low (A)	Low (B)	Low (B)	High (A)	Low (A)	Medium (A)
Shigella species	Very low (A)	Very low (A)	Very low (A)	High (A)	Medium (B)	High (B)	High (B)	High (A)
Staphylococcus aureus	Very high (A)	Very high (A)	Very low (A)	High (A)	Low (A)	High (A)	Very high (A)	High (A)

Survey dissemination

Dissemination channels

Surveys were disseminated as described in the Methods section. Dissemination channels are shown in sFigure 2.

sFigure 2 Survey dissemination

Abbreviations: ADVAC, Advanced Course of Vaccinology; AFRO, WHO African Regional Office; CDC, Center for Disease Control; CHAI, Clinton Health Access Initiative; CSO, Civil Society Organisation; DCVMN, Developing Country Vaccine Manufacturers Network; GVIRF, Global Vaccines and Immunology Reasearch Forum; IFPMA, International Federation of Pharmaceutical Manufacturer's Associations; MoH, Ministry of Health; NITAG, National Immunisation Technical Advisory Group; NRA, National regulatory authority; PAVM, Partnership for African Vaccine Manufacturing; PDVAC, WHO Product Development for Vaccines Advisory Group; RITAG, Regional Immunisation Technical Advisory Group; SP7 WG, Immunisation Agenda 2030 Strategic Priority 7 Working Group; WHO, World Health Organization; WHO-IVB, WHO Immunization, Vaccines and Biologicals department

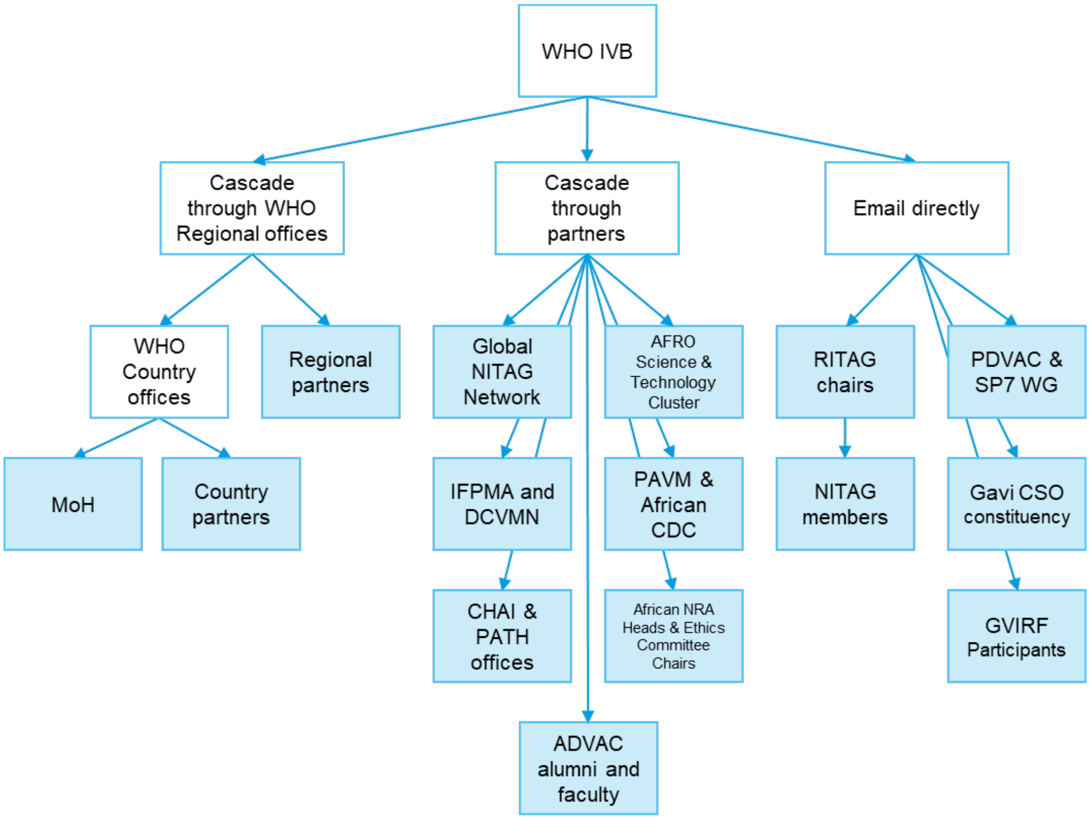

Survey Invitation

Emails inviting immunization stakeholders to participate in the survey and the attached invitation and survey links shown in sFigure 3 and sFigure 4.

sFigure 3 Survey invitation: Example email to immunization stakeholders

From: IA2030-SP7 <IA2030-SP7@who.int>
Sent: Friday, December 23, 2022 7:26 AM
Cc: SPARROW JONES, Erin Grace <sparrowe@who.int>; GIERSING, Birgitte <giersingb@who.int>; HASSO-AGOPSOWICZ, Mateusz <hassoagopsowiczm@who.int>; Angela Hwang <angela@ahwang.net>
Subject: Please complete and share this survey - Regional and Country Priorities for new vaccine research and development

Dear colleague,

The World Health Organization (WHO) is currently identifying **Regional and Country Priorities for new vaccine research and development**. Unlike global priority-setting projects, this project seeks to understand what is most important to people working at regional and country levels. It seeks to capture diverse perspectives, including from *Ministry of Health officials, policy makers, technical advisory groups, health care professionals, regulators, experts in public health and infectious diseases, pharmaceutical companies, and funders*.

The first step of this process is a “Preferences Survey”, which identifies which criteria for prioritization are most important to each person. **As key stakeholders in immunization, we are kindly requesting you to complete this survey.** The survey is available in versions for each WHO region and in a global version. Please decide whether you would like to respond as a regional stakeholder or as a global stakeholder and choose the appropriate survey from the table below. (If you need to find your WHO region, please see the attached memo.) **The survey will take 30 – 45 minutes to complete.**

In addition to completing the survey, **please share this invitation with other stakeholders** in public health and immunization. Results from this survey will be presented to WHO’s Strategic Advisory Group on Immunization (SAGE) in March 2023, so please complete and forward this survey **as soon as possible**.

WHO Region	Language	Link
African	English	https://bit.ly/AFRO_EN
	French	https://bit.ly/AFRO_FR
	Portuguese	https://bit.ly/AFRO_PT
Americas	English	https://bit.ly/AMRO_EN
	Portuguese	https://bit.ly/AMRO_PT
	Spanish	https://bit.ly/AMRO_ES
Eastern Mediterranean	Arabic	https://bit.ly/EMRO_AR
	English	https://bit.ly/EMRO_EN
	French	https://bit.ly/EMRO_FR
Europe	English	https://bit.ly/EURO_EN
	French	https://bit.ly/EURO_FR
	Portuguese	https://bit.ly/EURO_PT

WHO Region	Language	Link
	Spanish	https://bit.ly/EURO_ES
	Russian	https://bit.ly/EURO_RU
South-East Asian	English	https://bit.ly/SEARO_EN
	Portuguese	https://bit.ly/SEARO_PT
Western Pacific	Chinese	https://bit.ly/WPRO_ZH
	English	https://bit.ly/WPRO_EN
	French	https://bit.ly/WPRO_FR

The next step of the prioritization process will be regional consultations in 2023 to deliberate and align on the final priority list. Additional information and emerging results from this project are being posted to TechNet-21.

If you have any questions or suggestions about this project, please email IA2030-SP7@who.int and Angela Hwang (angela@ahwang.net).

Thank you and warm regards,

Gitte

Birgitte Giersing, PhD
Team Lead Vaccine Platforms & Prioritization
Vaccine Product and Delivery Research (PDR) Unit
Department of Immunization, Vaccines & Biologicals (IVB)
Universal Health Coverage/Lifecourse Division
World Health Organization, Geneva, Switzerland
Mobile: +41 79 764 1655

Websites: Immunization, Vaccines and Biologicals; World Health Organization
Immunization Agenda 2030; COVAX Country Readiness and Delivery

sFigure 4 **Survey invitation: Example email attachment**

World Health Organization

20, AVENUE APPIA – CH-1211 GENEVA 27 – SWITZERLAND – TEL CENTRAL +41 22 791 2111 – FAX CENTRAL +41 22 791 3111 – WWW.WHO.INT

Tel. direct:

Fax direct:

E-mail : IA2030-SP7@who.int

In reply please refer to:

Your reference:

22 November 2022

Regional priorities for vaccine development

Dear Colleagues,

This letter serves as a request for assistance in defining regional priorities for vaccine research and development. Under Immunization Agenda 2030, the World Health Organization (WHO) is responsible for assembling a "short list" of pathogens that should be prioritized for new vaccine development.

To aid in identifying these priorities, WHO is conducting a survey to understand what is most important from a regional and country perspective. The survey is meant to capture diverse perspectives and will take 30 - 45 minutes to complete.

As an expert in public health, your perspectives are important to this work. **Please see the table on the next page to access the survey for your region.** If you would like to complete the survey from a global perspective, please go to https://bit.ly/GLOBAL_EN instead.

Additional information about this project may be found at www.technet-21.org/en/topics/regional-and-country-priorities.

Should you have any questions or suggestions, please contact us by email at **IA2030-SP7@who.int.**

Yours sincerely,

Dr Kate O'Brien
Director, Immunization, Vaccines and Biologicals,
Universal Health Coverage and Life Course

منظمة الصحة العالمية • 世界卫生组织
Organisation mondiale de la Santé • Всемирная организация здравоохранения • Organización Mundial de la Salud

Africa<br><br>Afrique<br><br>África	English:<br>https://bit.ly/AFRO_EN<br><br>Français:<br>https://bit.ly/AFRO_FR<br><br>Português:<br>https://bit.ly/AFRO_PT	Algeria<br>Angola<br>Benin<br>Botswana<br>Burkina Faso<br>Burundi<br>Cabo Verde<br>Cameroon<br>Central African Republic<br>Chad<br>Comoros<br>Congo, Dem. Rep.<br>Congo, Rep.<br>Côte d'Ivoire<br>Equatorial Guinea	Eritrea<br>Eswatini<br>Ethiopia Gabon<br>Gambia, The<br>Ghana<br>Guinea<br>Guinea-Bissau<br>Kenya<br>Lesotho<br>Liberia<br>Madagascar<br>Malawi<br>Mali<br>Mauritania<br>Mauritius<br>Mozambique	Namibia<br>Niger<br>Nigeria<br>Rwanda<br>São Tomé and Principe<br>Senegal<br>Seychelles<br>Sierra Leone<br>South Africa<br>South Sudan<br>Tanzania<br>Togo<br>Uganda<br>Zambia<br>Zimbabwe
Americas<br><br>Américas<br><br>Américas	English:<br>https://bit.ly/AMRO_EN<br><br>Español:<br>https://bit.ly/AMRO_ES<br><br>Português:<br>https://bit.ly/AMRO_PT	Antigua and Barbuda<br>Argentina<br>Bahamas, The<br>Barbados<br>Belize<br>Bolivia<br>Brazil<br>Canada<br>Chile<br>Colombia<br>Costa Rica<br>Cuba	Dominica<br>Dominican Republic<br>Ecuador<br>El Salvador<br>Grenada<br>Guatemala<br>Guyana<br>Haiti<br>Honduras<br>Jamaica<br>Mexico<br>Nicaragua	Panama<br>Paraguay Peru<br>St. Kitts and Nevis<br>St. Lucia<br>St. Vincent and the Grenadines<br>Suriname<br>Trinidad and Tobago<br>United States<br>Uruguay<br>Venezuela
شرق المتوسط<br><br>Eastern Mediterranean<br><br>Méditerranée orientale	ا العربية<br>https://bit.ly/EMRO_AR<br><br>English:<br>https://bit.ly/EMRO_EN<br><br>Français:<br>https://bit.ly/EMRO_FR	Afghanistan<br>Bahrain<br>Djibouti<br>Egypt, Arab Rep.<br>Iran, Islamic Rep.<br>Iraq<br>Jordan	Kuwait<br>Lebanon<br>Libya<br>Morocco<br>Oman<br>Pakistan<br>Qatar<br>Saudi Arabia<br>Somalia	Sudan<br>Syrian Arab Republic<br>Tunisia<br>United Arab Emirates<br>Yemen, Rep.
Europe<br><br>L'Europe<br><br>Europa<br><br>Europa<br><br>Европа	English:<br>https://bit.ly/EURO_EN<br><br>Français:<br>https://bit.ly/EURO_FR<br><br>Español:<br>https://bit.ly/EURO_ES<br><br>Português:<br>https://bit.ly/EURO_PT<br><br>Русский:<br>https://bit.ly/EURO_RU	Albania<br>Andorra<br>Armenia<br>Austria<br>Azerbaijan<br>Belarus<br>Belgium<br>Bosnia and Herzegovina<br>Bulgaria<br>Croatia<br>Cyprus<br>Czech Republic<br>Denmark<br>Estonia<br>Finland<br>France<br>Georgia<br>Germany	Hungary<br>Iceland<br>Ireland<br>Greece<br>Israel<br>Italy<br>Kazakhstan<br>Kyrgyz Republic<br>Latvia<br>Lithuania<br>Luxembourg<br>Malta<br>Moldova<br>Monaco<br>Montenegro<br>Netherlands<br>North Macedonia<br>Norway	Poland<br>Portugal<br>Romania<br>Russian Federation<br>San Marino<br>Serbia<br>Slovak Republic<br>Slovenia<br>Spain<br>Sweden<br>Switzerland<br>Tajikistan<br>Turkey<br>Turkmenistan<br>Ukraine<br>United Kingdom<br>Uzbekistan

South-East Asia<br><br><br>Sudeste Asiático	English:<br>https://bit.ly/SEARO_EN<br><br>Português:<br>https://bit.ly/SEARO_PT	Bangladesh<br>Bhutan<br>India<br>Indonesia	Korea, Dem. People's Rep.<br>Maldives<br>Myanmar	Nepal<br>Sri Lanka<br>Thailand<br>Timor-Leste
Western Pacific<br><br><br>Pacifique occidental<br><br>西太平洋地区	English:<br>https://bit.ly/WPRO_EN<br><br>Français:<br>https://bit.ly/WPRO_FR<br><br>中文：<br>https://survey.1000minds.com/16166/WHO_VAC_WPRO_ZH	Australia<br>Brunei Darussalam<br>Cambodia<br>China<br>Cook Islands<br>Fiji<br>Japan<br>Kiribati<br>Korea, Rep.	Lao PDR<br>Malaysia<br>Marshall Islands<br>Micronesia, Fed. Sts.<br>Mongolia<br>Nauru<br>New Zealand<br>Niue<br>Palau	Papua New Guinea<br>Philippines<br>Samoa<br>Singapore<br>Solomon Islands<br>Tonga<br>Tuvalu<br>Vanuatu<br>Vietnam

Survey components

Survey introduction

Each survey started with the introductory text and biographical questions shown in sFigure 5.

sFigure 5 Survey introduction

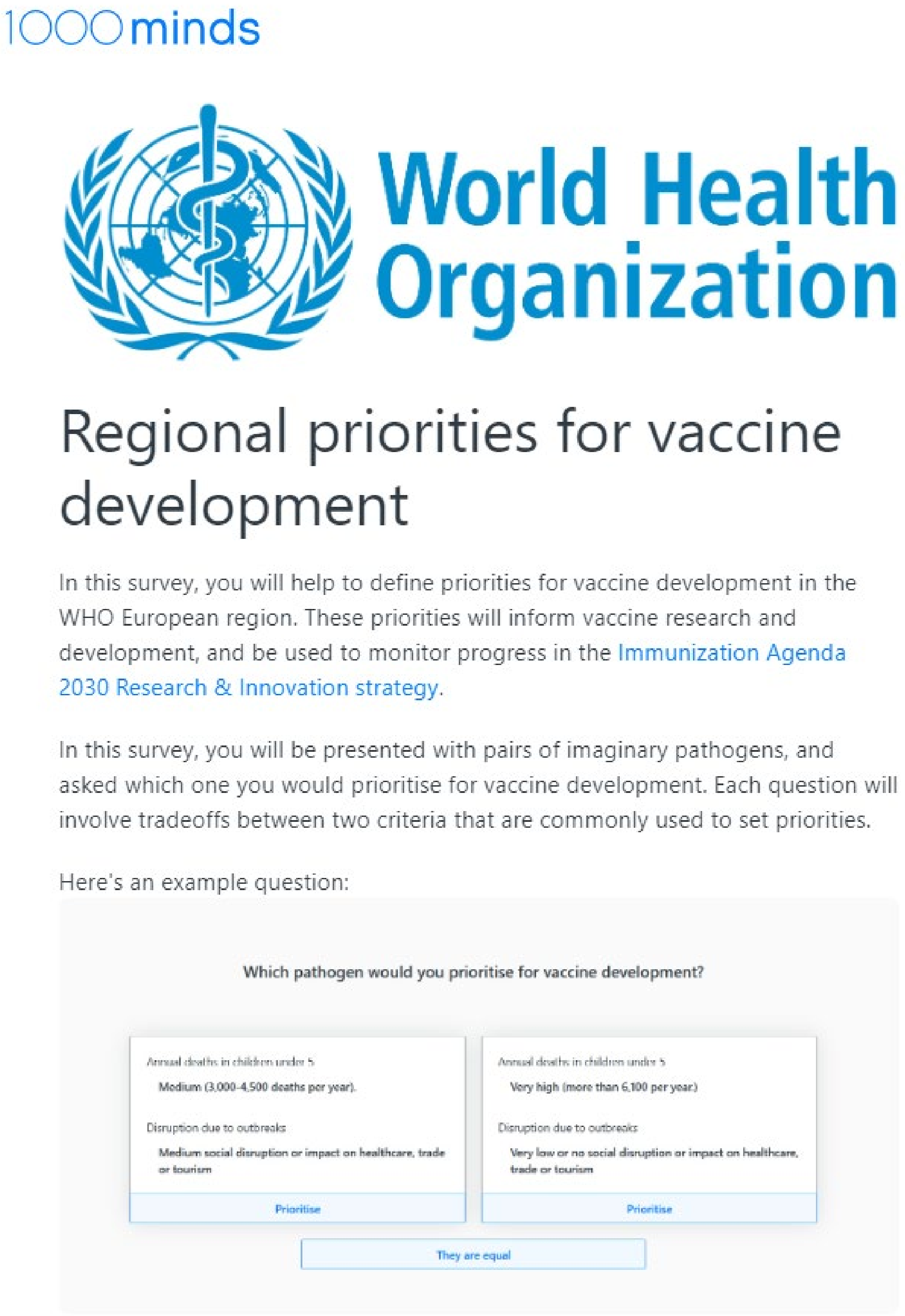

1000minds

When thinking about your answer, please consider just what is important for the European region. Assume that the pathogens are the same in every way except for the information given. If you need more information about the criteria, click the "Info" button at the bottom of the page.

Click on the pathogen you think is more important. If both are equally important, click, "They are equal". The survey should take around 30 minutes to complete, and your individual results will be shown after you are done.

If you have comments about the survey, enter them using the "Comment" button at the bottom of each page, or at the end of the survey. For more information on the survey, please see link.

Your participation is voluntary. By clicking on "start survey", you are showing you consent to taking part in the survey. Your responses will be taken as your personal views, and not reflecting the position of any organisations with which you are employed or affiliated. Reports of survey results will not include any personal identifiers such as names or email addresses.

Thank you very much.

Your name *

Your email address *

What country do you work in? *

What kind of organisation do you work for? (select all that apply) *

- ☐ Academic institution
- ☐ Funding agency
- ☐ Government
- ☐ Healthcare provider
- ☐ Non-governmental organisation
- ☐ Pharmaceutical industry
- ☐ Regulatory agency
- ☐ United Nations agency
- ☐ Other

What are your main areas of expertise? (select all that apply) *

- ☐ Disease epidemiology
- ☐ Economics and health financing
- ☐ Healthcare
- ☐ Health policy
- ☐ Regulatory affairs
- ☐ Vaccine research and development
- ☐ Other

How long have you been working in a health-related area? *

- ○ Up to 10 years
- ○ 11 - 20 years
- ○ 21 - 30 years
- ○ More than 30 years

Next

PAPRIKA survey questions

In the PAPRIKA method, the survey participant choses between hypothetical alternatives described in terms of two criteria at a time. (sFigure 6) Additional questions are posed until the participants preferences are fully captured. PAPRIKA minimises the number of questions using adaptive choice-based conjoint analysis, so each participant responds to a personalized sequence of questions.

sFigure 6 Example Survey Question

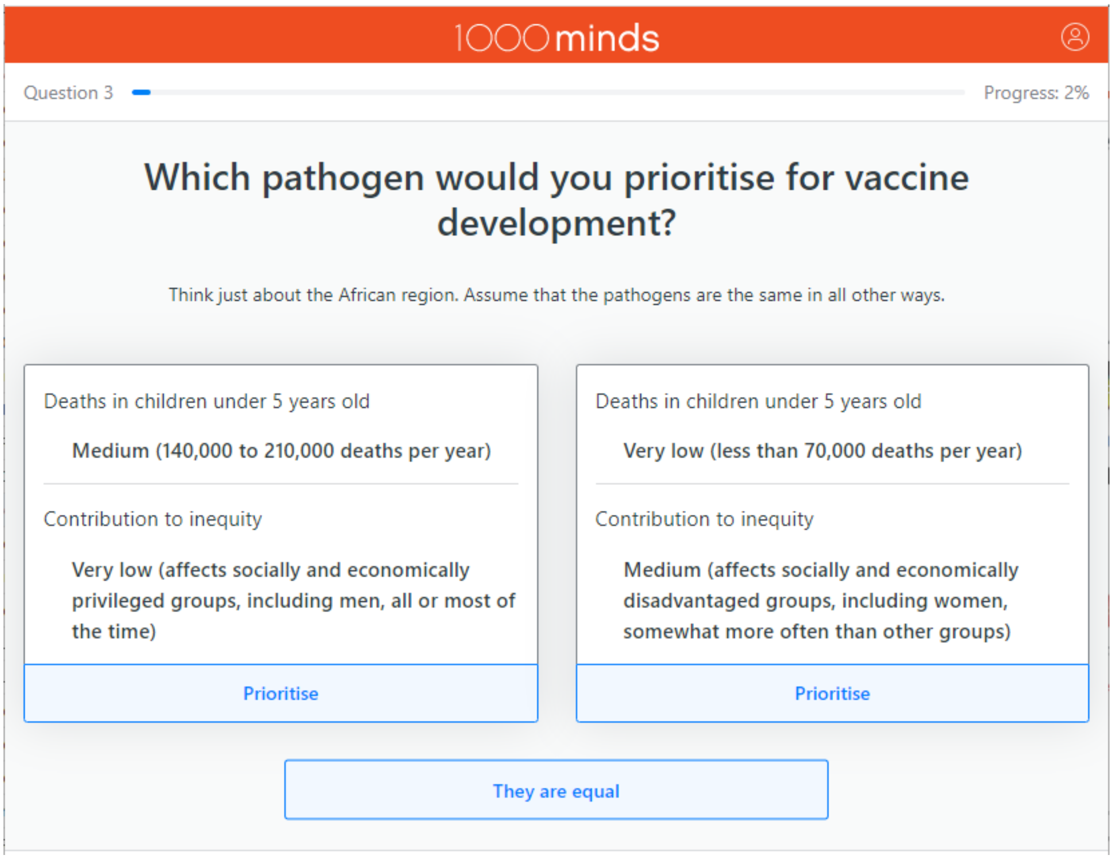

The software then applies mathematical techniques based on linear programming to the participant's choices to calculate the relative importance, or weight, of each level of each criterion. In reporting weights, the total weight of Very high scores is set to 100%, and the weight of all Very low scores is set to 0%. To streamline surveys, weights for Low and High scores are interpolated from weights for Very high, Medium, and Very low scores.

sFigure 7 illustrates how total weight is calculated for each pathogen in each region. A participant's total weight for a pathogen is calculated by summing the weights that correspond to the pathogen's scores in their region. Within each region, individual total weights for each pathogen are averaged to give a regional total weight for the pathogen. This regional total weight is then used to rank the pathogens within the region.

sFigure 7 Calculating total weight for a pathogen

Step 1. Score pathogens

Pathogens	1 Annual deaths in children under 5	2 Annual deaths in people 5 and older	3 Annual years lived with disability (all ages)	4 Social and economic burden per case	5 Disruption due to outbreaks	6 Contribution to inequity	7 Contribution to antimicrobial resistance	8 Unmet needs for prevention & treatment
	Criteria							
Mycobacterium tuberculosis (TB)*	Low	Very high	Very high	Very high	High	Very high	Very high	High
Staphylococcus aureus	High	High	Very low	High	Low	Medium	Very high	High
Klebsiella pneumoniae	Very high	Medium	Very low	High	Low	Low	Very high	High
Human immunodeficiency virus 1 (HIV-1)	Very low	Low	Very low	Very high	High	Very high	Very high	High

Step 2. Surveys give weight of each criteria/score combination

Score	1 Annual deaths in children under 5	2 Annual deaths in people 5 and older	3 Annual years lived with disability (all ages)	4 Social and economic burden per case	5 Disruption due to outbreaks	6 Contribution to inequity	7 Contribution to antimicrobial resistance	8 Unmet needs for prevention & treatment
	Criteria							
Very high	0.150	0.119	0.128	0.106	0.132	0.117	0.144	0.105
High	0.113	0.092	0.098	0.077	0.094	0.089	0.103	0.078
Medium	0.076	0.064	0.069	0.051	0.060	0.061	0.065	0.052
Low	0.039	0.034	0.037	0.026	0.029	0.031	0.032	0.027
Very low	0.000	0.000	0.000	0.000	0.000	0.000	0.000	0.000

Step 3. Sum weights corresponding to pathogen scores

0.039 | 0.119 | 0.128 | 0.106 | 0.094 | 0.117 | 0.144 | 0.078

Mycobacterium tuberculosis (TB) **0.824**

Post-survey information

Each survey concluded by presenting the participant's results and asking face validity questions shown in sFigure 8.

sFigure 8 Post-survey information (demo survey)

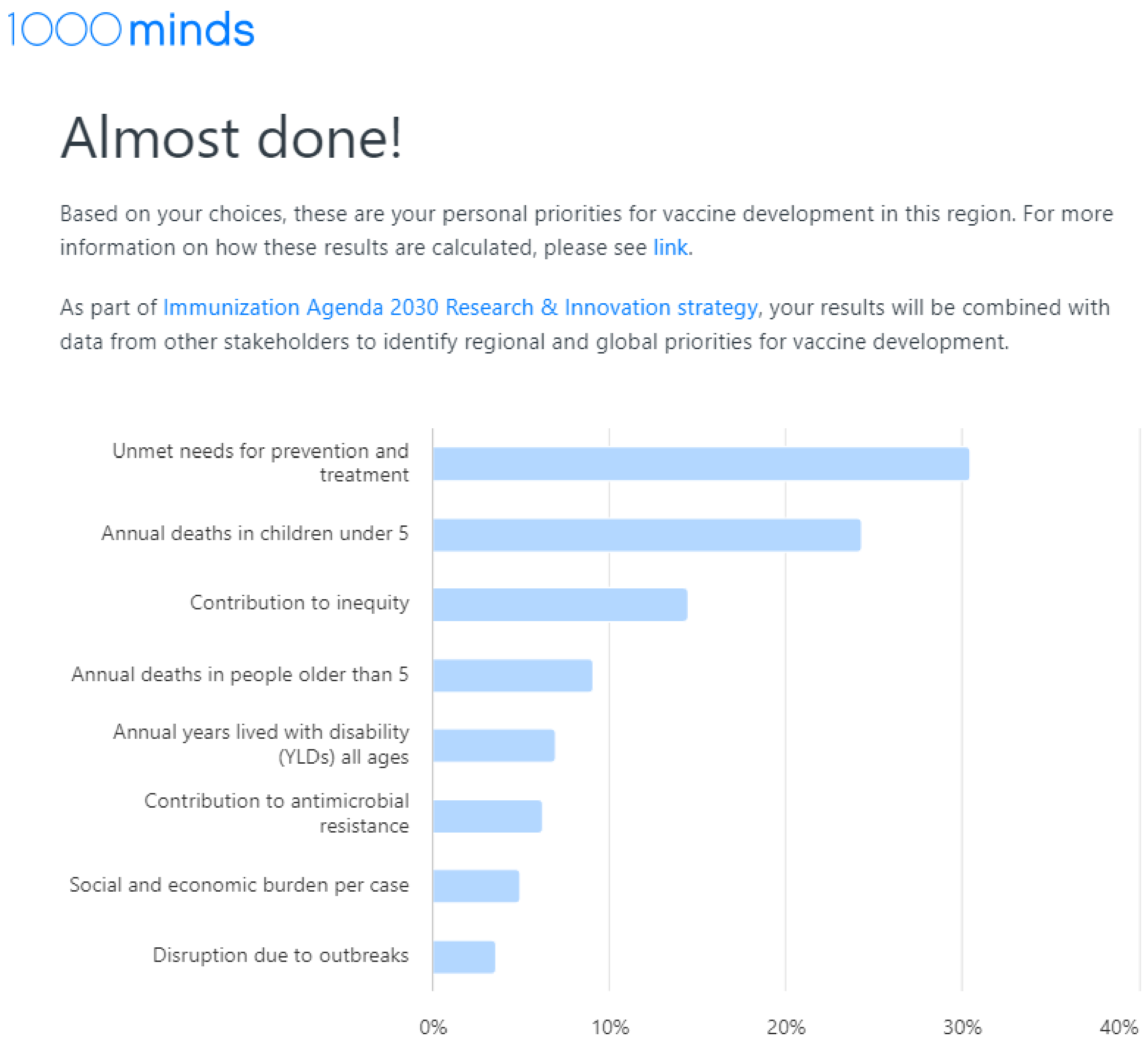

1. Mycobacterium tuberculosis (TB)
2. Klebsiella pneumoniae
3. Staphylococcus aureus
4. Human immunodeficiency virus 1 (HIV-1)
5. Extra-intestinal pathogenic E. coli (ExPEC)
6. Respiratory syncytial virus
7. Group A streptococcus (Streptococcus py[illegible]
8. Group B streptococcus (Streptococcus aga[illegible]
9. Pseudomonas aeruginosa
10. Mycobacterium leprae (leprosy)
11. Shigella
12. Leishmania
13. Plasmodium falciparum (malaria)
14. Influenza
15. Cytomegalovirus
16. Norovirus
17. Neisseria gonorrhoeae
18. Herpes simplex types 1 and 2
19. Chikungunya virus
20. Intestinal pathogenic E. coli (InPEC)
21. Salmonella Paratyphi
22. Non-typhoidal Salmonella
23. Schistosomes

Does the order of criteria in the bar chart seem correct to you? *

- ○ Yes
- ○ No
- ○ Not sure

Does the order of pathogens listed seem reasonable to you? *

- ○ Yes
- ○ No
- ○ Not sure

In your results, what was surprising? What was as expected?

Was the survey easy or difficult to understand? *

- ○ Very difficult
- ○ Difficult
- ○ Neutral
- ○ Easy
- ○ Very easy

Do you have any comments you would like to share?

Thank you for taking part in our survey. To learn more about this project, go to link.

Auto-fill Continue

Survey participants

Participants could start the survey multiple times. In total 577 responses were received from 533 survey participants.

sFigure 9 Survey responses

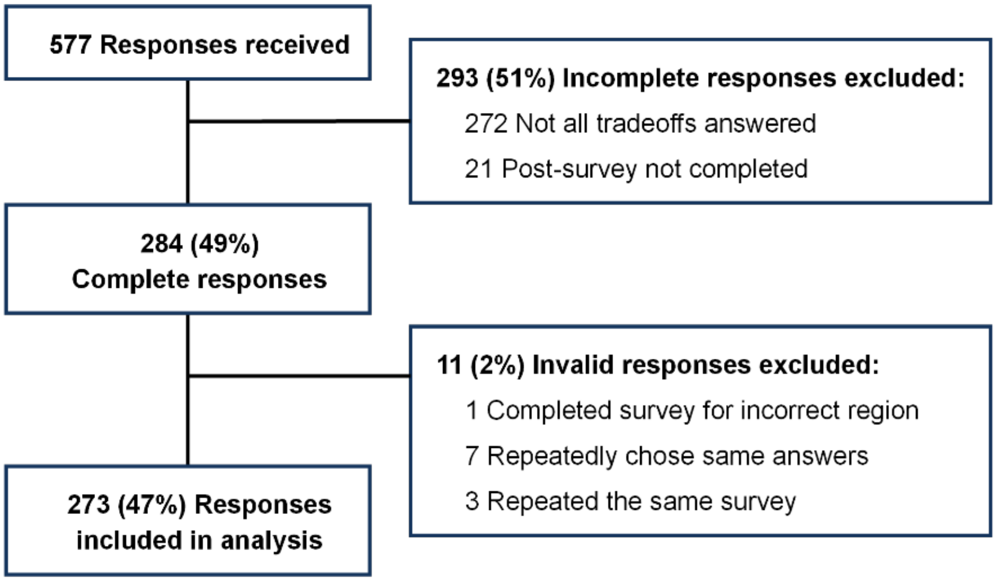

sTable 10 Regional and country representation in included survey responses

WHO Region	Responses included	Countries represented / Countries in region	% of countries	Countries with responses
African	55	27 / 47	57%	Benin, Burkina Faso, Burundi, Cameroon, Chad, Comoros, Congo, Democratic Republic, Congo, Rep., Eritrea, Eswatini, Ethiopia, Gabon, Ghana, Kenya, Madagascar, Malawi, Mali, Nigeria, Rwanda, Senegal, Seychelles, Sierra Leone, South Africa, Tanzania, Uganda, Zambia, Zimbabwe
Americas	45	18 / 35	51%	Argentina, Bahamas, Barbados, Bolivia, Brazil, Canada, Chile, Colombia, Cuba, Ecuador, El Salvador, Guatemala, Guyana, Jamaica, Mexico, Peru, Suriname, United States
E. Med.	38	15 / 21	71%	Afghanistan, Bahrain, Djibouti, Egypt, Arab Rep., Iran, Islamic Rep., Jordan, Kuwait, Lebanon, Oman, Pakistan, Saudi Arabia, Sudan, Syrian Arab Republic, Tunisia, United Arab Emirates
European	26	10 / 53	19%	France, Germany, Israel, Netherlands, Norway, Russian Federation, Sweden, Switzerland, Tajikistan, United Kingdom
SE Asian	44	9 / 11	82%	Bangladesh, Bhutan, India, Indonesia, Maldives, Myanmar, Nepal, Thailand, Timor-Leste
W. Pacific	65	10 / 27	37%	Australia, Cambodia, China, Fiji, Korea, Rep., Lao PDR, New Zealand, Papua New Guinea, Philippines, Vietnam
Total	*273*	*89 / 194*	*46%*	

sTable 11 Characteristics of survey participants

P-values were obtained using Chi-square or Fisher's Exact Test (when cell counts were less than 5) when assessing differences in proportions between "Africa" compared to other WHO regions.

Abbreviations: R&D, Research & Development

		Africa (n=55)	Americas (n=45)		Eastern Mediterranean (n=38)		Europe (n=26)		Southeast Asia (n=44)		Western Pacific (n=65)		Total (n=273)
**Characteristic**	**Option**	**n (%)**	**n (%)**	**P-value**	**n (%)**	**P-value**	**n (%)**	**P-value**	**n (%)**	**P-value**	**n (%)**	**P-value**	**n (%)**
**Years of Experience**													
	Up to 10 years	8 (15)	5 (11)	0.03	3 (8)	0.31	3 (12)	0.04	6 (14)	0.20	7 (11)	0.08	32(12)
	11-20 years	27 (49)	11 (24)		14 (37)		5 (19)		13 (30)		19 (29)		89 (33)
	21-30 years	10 (18)	11 (24)		12 (32)		9 (35)		12 (27)		19 (29)		73 (27)
	More than 30 years	10 (18)	18 (40)		9 (24)		9 (35)		13 (30)		20 (31)		79 (29)
**Expertise**													
Disease Epidemiology	No	24 (44)	18 (40)	0.84	15 (40)	0.83	14 (54)	0.48	23 (52)	0.42	36 (55)	0.27	130 (48)
	Yes	31(56)	27 (60)		23 (60)		12 (46)		21(48)		29 (45)		143 (52)
Economics & Health Financing	No	51 (93)	42 (93)	1.00	34 (90)	0.71	25 (96)	1.00	39 (89)	0.50	63 (97)	0.41	254 (93)
	Yes	4 (7)	3 (7)		4 (10)		1 (4)		5 (11)		2 (3)		19 (7)
Healthcare	No	35 (64)	17 (38)	0.02	18 (47)	0.14	17 (65)	1.00	26 (59)	0.68	50 (77)	0.16	163 (60)
	Yes	20 (36)	28 (62)		20 (53)		9 (35)		18 (41)		15 (23)		110 (40)
Health policy	No	38 (69)	34 (76)	0.51	24 (63)	0.66	15 (58)	0.33	30 (68)	1.00	52 (80)	0.21	193 (71)
	Yes	17 (31)	11 (24)		14 (37)		11 (42)		14 (32)		13 (20)		80 (29)
Regulatory affairs	No	50 (91)	44 (98)	0.22	36 (95)	0.70	25 (96)	0.66	39 (89)	0.75	59 (91)	1.00	253 (93)
	Yes	5 (9)	1 (2)		2 (5)		1 (4)		5 (11)		6 (9)		20 (7)
Vaccine R&D	No	34 (62)	26 (58)	0.22	27 (71)	0.38	7 (27)	0.004	27 (61)	1.00	29 (45)	0.07	150 (55)
	Yes	21 (38)	19 (42)		11 (29)		19 (73)		17 (39)		36 (55)		123 (45)
Other Expertise	No	45 (82)	42 (93)	0.14	34 (90)	0.38	22 (85)	1.00	41 (93)	0.14	58 (89)	0.30	242 (89)
	Yes	10 (18)	3 (7)		4 (10)		4 (15)		3 (7)		7 (11)		31 (11)

Characteristic	Option	Africa (n=55) n (%)	Americas (n=45) n (%)	Americas P-value	Eastern Mediterranean (n=38) n (%)	Eastern Mediterranean P-value	Europe (n=26) n (%)	Europe P-value	Southeast Asia (n=44) n (%)	Southeast Asia P-value	Western Pacific (n=65) n (%)	Western Pacific P-value	Total (n=273) n (%)
**Organisation Type**													
Academic Institution	No	38 (69)	18 (40)	0.01	29 (76)	0.49	16 (62)	0.62	29 (66)	0.83	36 (55)	0.14	166 (61)
	Yes	17 (31)	27 (60)		9 (24)		10 (38)		15 (34)		29 (45)		107 (39)
Funding Agency	No	54 (98)	45 (100)	1.00	37 (97)	1.00	24 (92)	0.24	44 (100)	1.00	60 (92)	0.22	264 (97)
	Yes	1 (2)	0 (0)		1 (3)		2 (8)		0 (0)		5 (8)		9 (3)
Government	No	36 (66)	34 (76)	0.38	24 (63)	0.83	15 (58)	0.62	21 (48)	0.10	53 (82)	0.06	183 (67)
	Yes	19 (34)	11 (24)		14 (37)		11 (42)		23 (52)		12 (18)		90 (33)
Healthcare Provider	No	48 (87)	26 (58)	0.001	31 (82)	0.56	22 (85)	0.74	41 (93)	0.51	55 (85)	0.80	223 (82)
	Yes	7 (13)	19 (42)		7 (18)		4 (15)		3 (7)		10 (15)		50 (18)
Non-governmental organisation	No	49 (89)	44 (98)	0.12	31 (82)	0.37	25 (96)	0.42	41 (93)	0.73	58 (89)	1.00	248 (91)
	Yes	6 (11)	1 (2)		7 (18)		1 (4)		3 (7)		7 (11)		25 (9)
Pharmaceutical Industry	No	55 (100)	41 (91)	0.04	36 (95)	0.16	23 (88)	0.03	44 (100)	1.00	52 (80)	0.0002	251 (92)
	Yes	0 (0)	4 (9)		2 (5)		3 (12)		0 (0)		13 (20)		22 (8)
Regulatory Agency	No	50 (91)	44 (98)	0.22	36 (95)	0.22	26 (100)	0.17	42 (96)	0.46	63 (97)	0.24	261 (96)
	Yes	5 (9)	1 (2)		2 (5)		0 (0)		2 (4)		2 (3)		12 (4)
UN agency	No	41 (75)	44 (98)	0.001	31 (82)	0.003	23 (88)	0.24	39 (89)	0.12	61 (94)	0.004	239 (88)
	Yes	14 (25)	1 (2)		7 (18)		3 (12)		5 (11)		4 (6)		34 (12)
Other	No	51 (93)	41 (91)	0.81	37 (97)	0.71	25 (96)	1.00	39 (89)	0.50	62 (95)	0.70	255 (93)
	Yes	4 (7)	4 (9)		1 (3)		1 (4)		5 (11)		3 (5)		18 (7)

Survey results

Criteria weights

sTable 12 Raw mean criteria weights

P-values were obtained using t-tests to assess differences in means between "Africa" compared to other WHO regions.

Abbreviation: SD, Standard Deviation

Criteria	African (n=55)	Americas (n=45)		Eastern Mediterranean (n=38)		European(n=26)		Southeast Asian (n=44)		Western Pacific (n=65)	
	**Mean % (SD)**	**Mean % (SD)**	**P**	**Mean % (SD)**	**P**	**Mean % (SD)**	**P**	**Mean % (SD)**	**P**	**Mean % (SD)**	**P**
Annual deaths in children under 5 years	14.2 (7.9)	16.4 (7.8)	0.18	15.0 (11)	0.73	**18.6 (7.8)**	**0.02**	14.0 (8.2)	0.88	14.3 (8.0)	0.96
Annual deaths in people 5 years & older	11.5 (6.2)	13.4 (6.0)	0.14	11.9 (6.2)	0.79	14.5 (7.3)	0.08	13.3 (5.2)	0.14	**14.9 (8.4)**	**0.01**
Years lived with disability	11.6 (5.3)	13.0 (6.5)	0.26	12.8 (5.2)	0.29	13.9 (6.8)	0.13	11.0 (5.1)	0.56	12.7 (5.7)	0.29
Social and economic burden per case	11.6 (5.9)	**9.0 (4.9)**	**0.02**	10.6 (5.6)	0.40	9.6 (5.1)	0.12	11.9 (4.8)	0.79	9.9 (5.5)	0.10
Disruption due to outbreaks	13.3 (6.5)	11.8 (5.9)	0.19	13.2 (6.8)	0.88	**7.4 (4.6)**	**0.0001**	12.6 (6.2)	0.56	12.4 (7.3)	0.44
Contribution to inequity	11.5 (6.4)	10.7 (6.0)	0.48	11.7 (7.3)	0.90	8.4 (8.2)	0.09	12.0 (7.5)	0.12	11.7 (7.2)	0.90
Contribution to antimicrobial resistance	13.8 (6.6)	14.9 (7.1)	0.44	14.4 (8.2)	0.74	15.5 (7.8)	0.36	13.6 (6.5)	0.85	11.8 (6.7)	0.10
Unmet needs for prevention & treatment	12.3 (7.3)	10.9 (5.8)	0.31	10.5 (6.9)	0.25	12.2 (7.8)	0.96	11.6 (5.3)	0.62	12.4 (6.7)	0.92

Pathogen ranks by region

These lists are survey results intended to inform deliberations on regional and global priorities and should not be read as regional priorities in themselves.

sTable 13 Pathogen total weights and ranks by region

Rank	African		Americas		E. Med.	
	**Pathogen**	**Total weight**	**Pathogen**	**Total weight**	**Pathogen**	**Total weight**
**1**	*Mycobacterium tuberculosis* (TB)	86.1%	HIV-1	71.8%	TB	82.4%
**2**	*Plasmodium falciparum* (malaria)	86.0%	*S aureus*	67.8%	*S aureus*	59.4%
**3**	Human immuno-deficiency virus 1 (HIV-1)	82.7%	*K pneumoniae*	58.2%	*K pneumoniae*	57.4%
**4**	*Klebsiella pneumoniae*	63.4%	ExPEC	55.6%	HIV-1	57.2%
**5**	*Staphylococcus aureus*	62.9%	TB	54.7%	*Leishmania*	56.2%
**6**	*Shigella* species	59.6%	Group A streptococcus	46.7%	ExPEC	54.2%
**7**	Non-typhoidal *Salmonella*	59.2%	*Shigella* species	42.5%	*Shigella* species	48.8%
**8**	Respiratory syncytial virus	51.1%	Respiratory syncytial virus	42.4%	Hepatitis C virus	48.2%
**9**	Extra-intestinal pathogenic *E coli* (ExPEC)	50.7%	Influenza	42.0%	Group A streptococcus	45.9%
**10**	Group B streptococcus	47.3%	Hepatitis C virus	39.8%	Norovirus	39.1%
**11**	Group A streptococcus	45.1%	Cytomegalovirus	38.5%	InPEC	36.7%
**12**	*Leishmania*	44.9%	*P falciparum*	38.4%	*N gonorrhoeae*	36.5%
**13**	Hepatitis C virus	44.2%	*Leishmania*	36.6%	Influenza	36.2%
**14**	Schistosomes	44.0%	Dengue virus	36.3%	Group B streptococcus	35.9%
**15**	Norovirus	40.6%	InPEC	35.9%	*P falciparum*	35.0%
**16**	Influenza	40.5%	*N gonorrhoeae*	35.6%	Cytomegalovirus	34.6%
**17**	Intestinal pathogenic *E coli* (InPEC)	37.8%	Group B streptococcus	35.1%	*M leprae*	33.3%
**18**	*Neisseria gonorrhoeae*	37.2%	*M leprae*	33.9%	Respiratory syncytial virus	32.9%
**19**	Cytomegalovirus	36.1%	Chikungunya virus	33.6%	Dengue virus	31.5%
**20**	Herpes simplex types 1 and 2 and *Mycobacterium leprae* (leprosy)	34.9% (tied)	Norovirus	33.5%	*C trachomatis*	30.5%
**21**			Herpes simplex types 1 and 2	30.6%	Non-typhoidal *Salmonella* and *Salmonella* Paratyphi	29.9% (tied)
**22**	Dengue virus	33.5%	Non-typhoidal *Salmonella*	28.8%		
**23**	*Chlamydia trachomatis*	32.2%	*C trachomatis*	28.1%	Schistosomes	26.5%
**24**	Chikungunya virus	30.8%	Schistosomes	25.2%	Chikungunya virus	24.6%
**25**	Hookworm	26.5%	*Salmonella* Paratyphi	18.9%	Hookworm	23.8%
**26**	*Salmonella* Paratyphi	23.8%	Hookworm	18.6%	Herpes simplex types 1 and 2	22.2%

Rank	European		SE Asian		W. Pacific	
	Pathogen	Total weight	Pathogen	Total weight	Pathogen	Total weight
**1**	*S aureus*	69.0%	TB	96.6%	TB	85.5%
**2**	TB	65.2%	HIV-1	67.7%	*S aureus*	69.0%
**3**	HIV-1	60.6%	*K pneumoniae*	64.9%	HIV-1	61.9%
**4**	ExPEC	58.3%	*S aureus*	61.8%	Group A streptococcus	56.5%
**5**	*K pneumoniae*	56.5%	Group A streptococcus	60.8%	*K pneumoniae*	55.5%
**6**	Group A streptococcus	47.4%	ExPEC	59.2%	ExPEC	49.0%
**7**	Cytomegalovirus	44.9%	Respiratory syncytial virus	52.5%	Influenza	48.5%
**8**	Respiratory syncytial virus	43.4%	*Shigella* species	49.5%	Respiratory syncytial virus	48.3%
**9**	Hepatitis C virus	38.2%	Hepatitis C virus	49.2%	Hepatitis C virus	43.3%
**10**	*Shigella* species	36.3%	Dengue virus	45.9%	Cytomegalovirus	43.1%
**11**	Norovirus	33.6%	Group B streptococcus	45.7%	Dengue virus	41.1%
**12**	Influenza	33.1%	*Leishmania* and Influenza	44.9% (tied)	*Shigella* species	39.3%
**13**	*N gonorrhoeae*	32.4%			Group B streptococcus	38.3%
**14**	InPEC	31.7%	*P falciparum*	39.8%	*M leprae*	35.8%
**15**	Group B streptococcus	28.2%	*M leprae*	38.5%	Norovirus	35.2%
**16**	*C trachomatis*	27.2%	Norovirus and InPEC	38.1% (tied)	InPEC	34.4%
**17**	*M leprae*	26.4%			*N gonorrhoeae*	34.2%
**18**	Non-typhoidal *Salmonella*	25.2%	*N gonorrhoeae*	37.7%	Herpes simplex types 1 and 2	31.0%
**19**	Herpes simplex types 1 and 2	24.1%	Cytomegalovirus	37.4%	*P falciparum*	30.2%
**20**	Chikungunya virus	23.2%	Chikungunya virus	33.9%	Chikungunya virus	28.8%
**21**	*Leishmania*	20.9%	*C trachomatis*	32.9%	Non-typhoidal *Salmonella*	28.4%
**22**	Hookworm	16.2%	*Salmonella* Paratyphi	30.9%	*Leishmania*	24.5%
**23**	*Salmonella* Paratyphi	15.1%	Herpes simplex types 1 and 2	30.1%	Hookworm	23.4%
**24**	Dengue virus	10.4%	Non-typhoidal *Salmonella*	27.7%	Schistosomes	22.8%
**25**	*P falciparum*	2.2%	Schistosomes	24.4%	*Salmonella* Paratyphi	22.6%
**26**	Schistosomes	1.1%	Hookworm	24.1%	*C trachomatis*	22.3%

sTable 14 Robustness testing results

See sTable 1 for abbreviations. ✓: retained in Global list, ×: drops from Global List

Global list	Testing conditions									
	**Cluster 1 responses only**	**Cluster 2 responses only**	**Omit *Annual deaths in children under 5***	**Omit *Annual deaths in people 5 and older***	**Omit *Annual years lived with disability (all ages)***	**Omit *Social and economic burden per case***	**Omit *Disruption due to outbreaks***	**Omit *Contribution to inequity***	**Omit *Contribution to antimicrobial resistance***	**Omit *Unmet needs for prevention & treatment***
Cytomegalovirus	✓	✓	✓	✓	✓	✓	✓	✓	✓	✓
Dengue virus	✓	×	✓	×	✓	✓	×	✓	×	✓
ExPEC	✓	✓	✓	✓	✓	✓	✓	✓	✓	✓
GAS	✓	✓	✓	✓	✓	✓	✓	✓	✓	✓
GBS	×	✓	×	✓	✓	✓	✓	✓	✓	×
Hepatitis C virus	✓	✓	✓	✓	✓	✓	✓	✓	✓	✓
HIV-1	✓	✓	✓	✓	✓	✓	✓	✓	✓	✓
Influenza	✓	✓	✓	✓	✓	✓	✓	✓	✓	✓
K pneumoniae	✓	✓	✓	✓	✓	✓	✓	✓	✓	✓
Leishmania	✓	✓	✓	✓	✓	✓	✓	✓	✓	✓
M tuberculosis	✓	✓	✓	✓	✓	✓	✓	✓	✓	✓
NTS	✓	✓	✓	✓	✓	✓	✓	✓	✓	✓
Norovirus	✓	×	✓	×	✓	✓	×	✓	✓	✓
P falciparum	✓	✓	✓	✓	✓	✓	✓	✓	✓	✓
RSV	✓	✓	×	✓	✓	✓	✓	✓	✓	✓
Shigella	✓	✓	✓	✓	✓	✓	✓	✓	✓	✓
S aureus	✓	✓	✓	✓	✓	✓	✓	✓	✓	✓
Added pathogens	none	none	InPEC	InPEC	InPEC<br>*N gonorrhoeae*	none	*M leprae*	none	none	InPEC

Use Cases and Action Categories

Use cases and category assignments were reviewed and endorsed by PDVAC in December 2023. For full supporting data, see the report to PDVAC at www.technet-21.org/en/resources/report/vaccine-r-d-priorities-vaccine-use-cases-and-action-categories-for-pdvac-input-december-2024. Any references to commercial products or trademarks are for tracking purposes only and not intended to reflect WHO product recommendations.

sTable 15 Use cases and Action Categories for pathogens on the Global list

Pathogen	Use Case	Action Category (as of December 2023)		
		**Research**	**Advance Product Development**	**Prepare to Implement**
Totals		*10 use cases*<br>*6 pathogens*	*17 use cases*<br>*10 pathogens*	*7 use cases*<br>*5 pathogens*
Cytomegalovirus	Prevention and/or modification of sequelae associated with congenital CMV, by vaccinating women and girls prior to pregnancy[1]		X	
Dengue virus	Vaccine for dengue naïve and seropositive individuals, to prevent dengue febrile illness induced by any dengue serotype[2]			X
Extra-intestinal pathogenic *E coli* (ExPEC)	Prevention of invasive *E coli* disease, including urinary tract infections or bacteraemia, in high-risk populations[3]			X
	Maternal immunisation during pregnancy to prevent invasive *E coli* disease, such as neonatal sepsis and meningitis, in neonates and young infants (*informed by PDVAC deliberations*)	X		
Group A strep (GAS)	Prevention of GAS disease: pharyngitis, impetigo and invasive disease in young children. Potential for prevention of GAS immune-mediated sequelae: acute rheumatic fever and rheumatic heart disease[4]	X		
Group B strep (GBS)	Maternal immunisation during pregnancy to prevent GBS-related stillbirth and invasive GBS disease in neonates and young infants[5]			X
	Prevention of Group B streptococcal infections in older adults[6]	X		
Hepatitis C virus	Prevention of chronic hepatitis C infection for persons at risk[7]	X		
	Therapeutic vaccines to improve treatment outcomes for chronic HCV infections[8]	X		
HIV-1	Prevention of HIV in high-risk populations (*informed by clinical trials*)	X		
	Treatment and/or cure of HIV infection in HIV-1 positive individuals (includes vaccines, mAbs, and combined approaches) (*informed by clinical trials*)	X		

Pathogen	Use Case	Action Category (as of December 2023)		
		Research	Advance Product Development	Prepare to Implement
	Preventive mAbs for HIV-1 infection in confirmed HIV-negative individuals at substantial risk of HIV infection and their sexual partners and/or prevention of HIV-1 infection in neonates and infants with HIV exposure[9]	X		
Influenza<br>Note: Preferred product characteristics for influenza vaccines are currently being revised. Once they are available these use cases will be updated.	Universal-type ("broadly protective") influenza A vaccines for prevention of severe influenza illness caused by human influenza A virus infection in persons aged 6 weeks and older belonging to a group at high risk for severe influenza illness (children aged 6 weeks through 59 months, elderly adults, persons with chronic medical conditions, and pregnant women). Duration of efficacy should be a minimum of 5 years[10]		X	
	Improved seasonal influenza vaccines, with a duration of protection of at least one year[10]		X	
K pneumoniae	Vaccine administered during pregnancy to prevent neonatal sepsis caused by the major disease-causing serotypes of *K pneumoniae*[11,12]	X		
	Preventing *K pneumoniae*-attributable disease, including pneumonia, bloodstream infections, and/or urinary tract infections in high-risk populations such as older adults, the immunocompromised, and those with anticipated prolonged hospital stay or planned surgeries[12,13]	X		
Leishmania species	Prevention of visceral leishmaniasis and/or cutaneous leishmaniasis in all age groups in endemic regions starting from 6 months of age, and/or prevention or treatment of post-kala azar dermal leishmaniasis[14]		X	
M tuberculosis	Prevention of active pulmonary TB disease (with or without evidence of latent infection), including in those with HIV infection[15]			X
	Prevention of TB disease in infants and young children, including in infants with HIV infection[15]			X
	Adjunctive treatment of TB, or to prevent relapse following cure in patients being treated for active TB, both drug sensitive and drug resistant strains[16]		X	
Non-typhoidal *Salmonella*	Paediatric vaccines for prevention of invasive disease caused by non-typhoidal *Salmonella* in children aged 6 – 36 months[17]		X	
	Prevention of invasive disease caused by non-typhoidal *Salmonella* in other individuals at high risk, including immunocompromised individuals, children over 36 months, the elderly, immunocompromised individuals, and persons living or traveling in settings with poor sanitation and hygiene[17]		X	
Norovirus	Prevention of norovirus acute gastroenteritis for children in all countries from 6 weeks of age[18]		X	

Pathogen	Use Case	Action Category (as of December 2023)		
		**Research**	**Advance Product Development**	**Prepare to Implement**
	Prevention of norovirus acute gastroenteritis for adolescents, adults, and/or older persons in all countries (including travellers) [18]		X	
P falciparum	Prevention of blood-stage infection due to *P falciparum* malaria at the individual level, for populations or age groups who experience high incidence of infection[19]		X	
	Prevention of malaria transmission at the community level for children and adults, including women of childbearing age, who represent the infectious reservoir and will need to be targeted to maximize the vaccine's impact on transmission[19]		X	
	mAbs for prevention of blood-stage infection due to *P falciparum* at the individual level, and/or reduction of clinical malaria, including severe malaria and death due to *P falciparum*[19]		X	
RSV	Active immunisation of women during pregnancy, for prevention of severe RSV disease in offspring during the neonatal period and early infancy[20]			X
	Active immunisation of infants, for prevention of RSV disease in infants and young children[20]		X	
	mAbs for prevention of severe RSV disease for all infants in the first 6 months of life and for high-risk young children entering their second RSV season (e.g with chronic heart or chronic lung disease) [21]			X
Shigella species	Prevention of moderate to severe diarrhoea due to *Shigella* in infants from 6 months and children up to 36 months of age[22,23]		X	
	Prevention of *Shigella*-attributable dysentery and diarrhoea for high-risk populations such as travellers and the military, communities with high incidence, elderly and institutionalized individuals, and/or pregnant women[22,23]		X	
S aureus	Prevention of severe infection in populations at risk, such as children, those over 60 years of age, and/or those in all age groups who are immunocompromised, experiencing recurrent skin and soft tissue infections, suffering from relevant comorbidities, exposed to epidemic strains, diabetics, or undergoing elective surgery or other invasive procedures with high risk of *S aureus* infection[24]		X	
	mAbs for prevention or treatment of disease caused by *S aureus*, such as severe pneumonia and/or superinfection in conjunction with viral pneumonia[25,26]		X	

Detailed methods for assessing predictors and cluster patterns in MCDA-generated criteria weights

Overview

The primary goals of this portion of the study analysis included determining: (1) whether participant characteristics were associated with their reported weights for each of the eight areas of prioritization, (2) if groups of respondents shared similar overall patterns in criteria weights, and (3) if groups of respondents sharing similar patterns in criteria weights also shared similar biographical backgrounds or impressions of the survey.

Data from 577 survey responses submitted from November 15, 2022 to May 1, 2023 were extracted from the 1000Minds website (https://www.1000minds.com, Last Accessed: August 29, 2023). Data was anonymized prior to use. Analysis was limited to the first regional survey submission by a participant in which all trade-off questions and the post-survey had been completed. Biographical information was self-reported by participants and was not verified for this analysis.

Outcomes

The first set of outcomes (used to identify participant characteristics associated with MCDA-generated weights) included **criteria weights calculated by the 1000minds tool across the "Very high" level for the eight criteria for prioritization**, namely *Annual deaths in children under five years*, *Annual deaths in people five years and older*, *Years lived with disability for all ages*, *Social and economic burden per case*, *Disruption due to outbreaks*, *Contribution to inequity*, *Contribution to antimicrobial resistance*, and *Unmet needs for prevention and treatment*.

The second set of outcomes (used to identify cluster patterns in criteria weights) was a **composite indicator of Medium and Very high levels for each of the eight criteria**. We generated the indicator using Principal Components Analysis (PCA) on weights generated by MCDA to allow both levels of criteria weights to be integrated into the same cluster model while avoiding issues of collinearity between measured scores. The composite indicator of MCDA criteria weights included the first set of eigenvectors accounting for at least 80 percent of the explained variance in the PCA model. Construction of the composite indicator was performed using the R packages 'stats' (version 4.3.1) and 'factoextra' (version 1.0.7), and 'FactoMineR' (version 2.11).[27,28]

The third set of outcomes (used to characterize potential predictors of cluster patterns) was a **participant's membership in a specific cluster of survey respondents**, determined based on groups identified through k-means clustering (goal 2) of the composite indicator of criteria weights.

Potential predictors of criteria weights and their cluster patterns

Participant's Background: Participant characteristics considered in the analysis included: language of survey (English, Arabic, Chinese, French, Portuguese, Russian, Spanish), WHO region of survey (Africa, Americas, Eastern Mediterranean, Europe, South-East Asia, Western Pacific), a participant's years of experience (up to 10 years, 11 to 20 years, 21 to 30 years, more than 30 years), their expertise (or not) in the fields of disease epidemiology, economics and health financing, health care, health policy, regulatory affairs, vaccine research and development, or another field; type of organisations that a participant works for (or not) including an academic institution, funding agency, government, health care provider, non-governmental organisation, pharmaceutical industry, regulatory agency, United Nations agency, or other entity; as well as eligibility status for Gavi support in 2023 (initial self-financing, preparatory transition, accelerated transition, not eligible) and World Bank group classification by income level in 2023 (low income, lower middle income, upper middle income, high income) for the participant's country of work. Participants who selected "Other" for expertise or type of organisation were provided the opportunity to give additional information in a comment field. When descriptions in these fields corresponded to existing categories for expertise or type of organisation, these responses were reassigned to those categories. Gavi eligibility and World Bank income group classifications were inferred based on the participant's self-reported country of work.

Face Validity of Survey: Factors linked to a participant's face-validity assessment of the MCDA survey were also assessed based on responses to the following questions in the post-survey: "Was the survey easy or difficult to understand?" (very difficult, difficult, neutral, easy, very easy); "Does the order of criteria in the bar chart seem correct to you?" (no, not sure, yes); and "Does the order of pathogens listed seem reasonable to you?" (no, not sure, yes).

Statistical analysis

Categorical data were presented as numbers and percentages. Continuous data were summarized as means with standard deviation. Differences in proportions and means were assessed using Chi-square/Fisher's Exact Test and t-test, respectively. To assess for potential selection bias due to incomplete surveys, a generalized estimating equation (GEE) with a binomial distribution and logit function was used to assess associations between participant characteristics and completion of trade-off questions after accounting for multiple responses per person. GEE was performed using the R package 'gee' (version 4.13-25).[29]

To **determine if participant characteristics were associated with their reported weights** for each of the eight criteria, multivariate linear regression was used to model mean differences in weights by a participant's years of experience (reference: up to 10 years); expertise in each of six areas (reference: not in the listed area); employment in each of nine types of organisations (reference: does not work for specified type of organisation); 2023 eligibility for Gavi support (reference: not eligible) and World Bank group classification (reference: high income) for the participant's country of work; language of survey (reference: English); WHO region of survey (reference: Africa); whether the survey was easy or difficult to understand (reference: neutral); if the order of criteria weights were correct (reference: yes); and, if pathogen rankings were reasonable (reference: yes). Multivariate linear regression was performed using the R package 'stats' (version 4.3.1).[27]

To **characterize group patterns in criteria weights,** K-means algorithm (an unsupervised machine learning method) was used to identify groups of survey respondents sharing similar sets of criteria weights. Optimal number of clusters was determined based on consensus of cut-off values using the elbow method, silhouette coefficient, Gap statistic and NbClust with centroid values ranging from 0 to 25.[30-33] Cluster analysis was performed using the R packages 'stats' (version 4.3.1), 'factoextra' (version 1.0.7), and "NbClust" (version 3.0.1).[27,28,33]

To **assess whether groups of respondents sharing similar patterns in criteria weights also shared similar biographical backgrounds or impressions of the survey,** a generalized linear model (GLM) with a binomial distribution and logit link function (based on the two-cluster model) was used to compare odds of membership in a specific cluster by years of experience, language and WHO region of survey, field of expertise, eligibility for Gavi support and World Bank income group classifications for country of work, type of work organisation, and face-validity assessment. GLM was performed using the R package 'stats' (version 4.3.1).[27]

Statistical power calculations for this analysis suggest that the minimum detectable odds ratio for membership in a specific cluster (based on two-cluster model) for the GLM analysis is between 0.213 and 2.475. The estimate is based on a sample size of 95 subjects in one cluster, 178 subjects in a reference cluster, a 15% likelihood of presenting a specific background characteristic, 80% statistical power and a two-sided significance level of 0.05.

Analysis was completed using R version 4.2.2 (R Foundation for Statistical Computing, Vienna, Austria).

Detailed results from assessing predictors and cluster patterns in MCDA-generated criteria weights

Determining if participant characteristics were associated with their reported weights

sFigure 10 Adjusted mean difference in criteria weights by region, Criteria 1-4 (n=273)

Multivariate linear regression was used to compare mean differences in criteria weights by participant characteristics. Analysis was based on first survey submission per survey type by a participant in which all trade-off questions and post-survey were complete. Models were adjusted for years of experience, language and WHO region of survey, field of expertise, eligibility for Gavi support and World Bank group classifications for country of work, type of work organisation, and participant's assessment regarding ease or difficulty to understand survey. Criteria weights are expressed as a percentage. Bars on plot indicate 95% confidence interval.

Abbreviations: MD, Mean Difference; CI, Confidence Interval; SD, Standard Deviation; No., Number of Participants; E. Med., Eastern Mediterranean; SE Asian, South East Asian; W. Pacific, Western Pacific

Characteristic	No. (%)	Mean (SD)	Adjusted MD (95% CI)
Annual deaths in children under 5 years			
African	55 (20)	14.3 (7.9)	Reference
Americas	45 (16)	16.4 (7.8)	-0.67 (-7.25 to 5.91)
E. Med.	38 (14)	15.0 (10.6)	1.00 (-3.95 to 5.96)
European	26 (10)	18.6 (7.8)	3.37 (-3.40 to 10.15)
SE Asian	44 (16)	14.0 (8.2)	0.12 (-4.99 to 5.22)
W. Pacific	65 (24)	14.3 (8.0)	0.06 (-5.64 to 5.76)
Annual deaths in people 5 years & older			
African	55 (20)	11.5 (6.2)	Reference
Americas	45 (16)	13.4 (6.0)	0.96 (-4.24 to 6.16)
E. Med.	38 (14)	11.9 (6.2)	0.68 (-3.23 to 4.60)
European	26 (10)	14.5 (7.3)	1.51 (-3.85 to 6.86)
SE Asian	44 (16)	13.3 (5.2)	1.96 (-2.07 to 6.00)
W. Pacific	65 (24)	14.9 (8.4)	2.52 (-1.98 to 7.03)
Years lived with disability (YLD)			
African	55 (20)	11.6 (5.3)	Reference
Americas	45 (16)	13.0 (6.5)	1.59 (-2.77 to 5.94)
E. Med.	38 (14)	12.8 (5.2)	2.07 (-1.21 to 5.35)
European	26 (10)	13.9 (6.8)	3.29 (-1.19 to 7.78)
SE Asian	44 (16)	11.0 (5.1)	0.19 (-3.19 to 3.57)
W. Pacific	65 (24)	12.7 (5.7)	1.70 (-2.07 to 5.47)
Social and economic burden per case			
African	55 (20)	11.6 (5.9)	Reference
Americas	45 (16)	9.0 (4.9)	-2.71 (-6.91 to 1.49)
E. Med.	38 (14)	10.6 (5.6)	-1.38 (-4.55 to 1.78)
European	26 (10)	9.6 (5.1)	-2.87 (-7.20 to 1.46)
SE Asian	44 (16)	11.9 (4.8)	-0.19 (-3.45 to 3.07)
W. Pacific	65 (24)	9.9 (5.5)	-1.67 (-5.31 to 1.97)

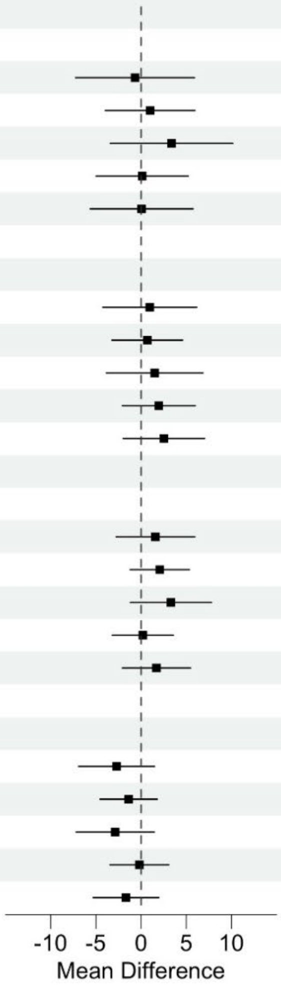

sFigure 11 Adjusted mean difference in criteria weights by region, Criteria 5-8 (n=273)

Multivariate linear regression was used to compare mean differences in criteria weights by participant characteristics. Analysis was based on first survey submission per survey type by a participant in which all trade-off questions and post-survey were complete. Models were adjusted for years of experience, language and WHO region of survey, field of expertise, eligibility for Gavi support and World Bank group classifications for country of work, type of work organisation, and participant's assessment regarding ease or difficulty to understand survey. Criteria weights are expressed as a percentage. Bars on plot indicate 95% confidence interval.

Abbreviations: MD, Mean Difference; CI, Confidence Interval; SD, Standard Deviation; No., Number of Participants; E. Med., Eastern Mediterranean; SE Asian, South East Asian; W. Pacific, Western Pacific

Characteristic	No. (%)	Mean (SD)	Adjusted MD (95% CI)
Disruption due to outbreaks			
African	55 (20)	13.4 (6.5)	Reference
Americas	45 (16)	11.7 (5.9)	-2.17 (-7.10 to 2.76)
E. Med.	38 (14)	13.2 (6.8)	-1.71 (-5.43 to 2.00)
European	26 (10)	7.4 (4.6)	-6.95 (-12.02 to -1.87)
SE Asian	44 (16)	12.6 (6.2)	-2.08 (-5.90 to 1.75)
W. Pacific	65 (24)	12.4 (7.3)	-4.58 (-8.85 to -0.32)
Contribution to inequity			
African	55 (20)	11.5 (6.4)	Reference
Americas	45 (16)	10.7 (6.0)	-0.53 (-5.81 to 4.75)
E. Med.	38 (14)	11.7 (7.3)	1.59 (-2.39 to 5.56)
European	26 (10)	8.4 (8.2)	-2.05 (-7.49 to 3.38)
SE Asian	44 (16)	12.0 (7.5)	2.43 (-1.67 to 6.53)
W. Pacific	65 (24)	11.7 (7.2)	2.52 (-2.05 to 7.09)
Contribution to antimicrobial resistance			
African	55 (20)	13.8 (6.6)	Reference
Americas	45 (16)	14.9 (7.1)	2.68 (-2.70 to 8.06)
E. Med.	38 (14)	14.4 (8.2)	-0.91 (-4.96 to 3.15)
European	26 (10)	15.5 (7.8)	0.69 (-4.85 to 6.23)
SE Asian	44 (16)	13.6 (6.5)	-1.52 (-5.70 to 2.66)
W. Pacific	65 (24)	11.8 (6.7)	-2.55 (-7.22 to 2.11)
Unmet needs for prevention & treatment			
African	55 (20)	12.3 (7.3)	Reference
Americas	45 (16)	10.9 (5.8)	0.85 (-4.13 to 5.83)
E. Med.	38 (14)	10.5 (6.9)	-1.34 (-5.09 to 2.41)
European	26 (10)	12.2 (7.7)	3.00 (-2.13 to 8.13)
SE Asian	44 (16)	11.6 (5.3)	-0.91 (-4.78 to 2.95)
W. Pacific	65 (24)	12.4 (6.7)	2.01 (-2.31 to 6.32)

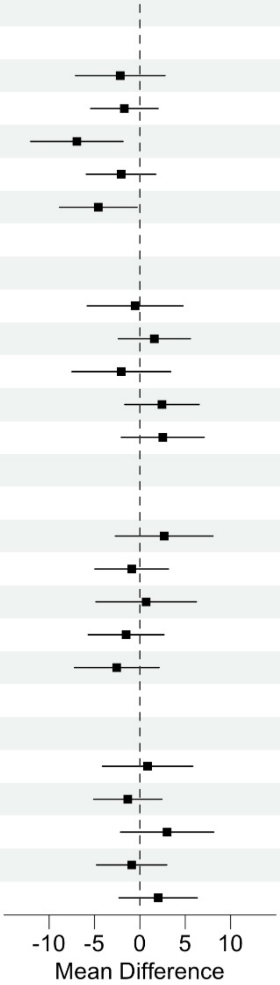

sFigure 12 Adjusted mean difference in criteria weights by language of survey, Criteria 1-4 (n=273)

Multivariate linear regression was used to compare mean differences in criteria weights by participant characteristics. Analysis was based on first survey submission per survey type by a participant in which all trade-off questions and post-survey were complete. Models were adjusted for years of experience, language and WHO region of survey, field of expertise, eligibility for Gavi support and World Bank group classifications for country of work, type of work organisation, and participant's assessment regarding ease or difficulty to understand survey. Criteria weights are expressed as a percentage. Bars on plot indicate 95% confidence interval.

Abbreviations: MD, Mean Difference; CI, Confidence Interval; SD, Standard Deviation; No., Number of Participants

Characteristic	No. (%)	Mean (SD)	Adjusted MD (95% CI)
Annual deaths in children under 5 years			
English	189 (69)	14.8 (8.5)	Reference
Arabic	2 (1)	8.6 (4.1)	-6.62 (-19.69 to 6.45)
Chinese	38 (14)	14.1 (8.6)	-0.04 (-6.08 to 6.00)
French	19 (7)	15.4 (7.5)	0.96 (-3.85 to 5.78)
Portuguese	1 (0)	16.4 (0.0)	4.95 (-13.24 to 23.13)
Russian	3 (1)	19.4 (9.6)	0.54 (-11.13 to 12.21)
Spanish	21 (8)	19.2 (8.2)	4.79 (-1.74 to 11.32)
Annual deaths in people 5 years & older			
English	189 (69)	12.9 (6.3)	Reference
Arabic	2 (1)	11.9 (7.5)	2.19 (-8.14 to 12.51)
Chinese	38 (14)	14.9 (9.9)	1.59 (-3.18 to 6.36)
French	19 (7)	13.4 (5.6)	2.45 (-1.35 to 6.25)
Portuguese	1 (0)	10.9 (0.0)	-2.20 (-16.56 to 12.17)
Russian	3 (1)	13.7 (9.9)	-1.44 (-10.66 to 7.78)
Spanish	21 (8)	13.5 (4.8)	0.92 (-4.24 to 6.08)
Years lived with disability (YLD)			
English	189 (69)	12.5 (5.7)	Reference
Arabic	2 (1)	4.6 (0.1)	-8.06 (-16.71 to 0.58)
Chinese	38 (14)	12.3 (6.1)	-1.66 (-5.66 to 2.33)
French	19 (7)	11.0 (4.6)	-0.74 (-3.93 to 2.44)
Portuguese	1 (0)	18.2 (0.0)	0.72 (-11.31 to 12.75)
Russian	3 (1)	9.7 (4.4)	-6.21 (-13.93 to 1.51)
Spanish	21 (8)	12.9 (6.3)	-1.09 (-5.41 to 3.22)
Social and economic burden per case			
English	189 (69)	10.6 (5.2)	Reference
Arabic	2 (1)	11.5 (1.1)	1.29 (-7.06 to 9.63)
Chinese	38 (14)	9.9 (5.8)	-0.55 (-4.41 to 3.31)
French	19 (7)	11.2 (6.2)	0.15 (-2.93 to 3.22)
Portuguese	1 (0)	3.6 (0.0)	-7.14 (-18.75 to 4.47)
Russian	3 (1)	14.9 (0.1)	6.18 (-1.27 to 13.63)
Spanish	21 (8)	9.1 (5.7)	-0.53 (-4.70 to 3.64)

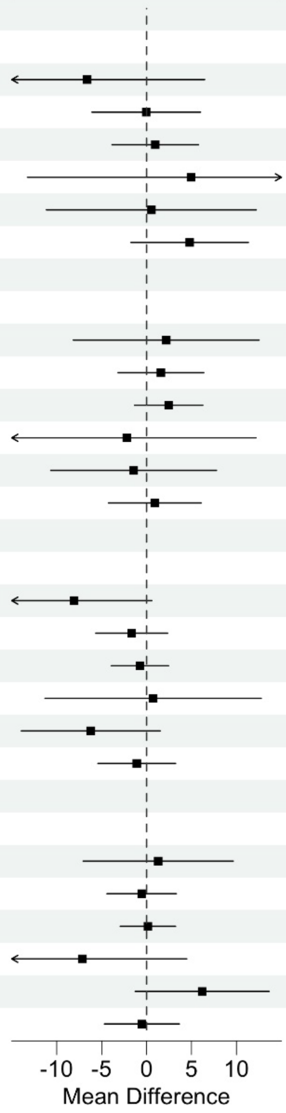

sFigure 13 Adjusted mean difference in criteria weights by language of survey, Criteria 5-8 (n=273)

Multivariate linear regression was used to compare mean differences in criteria weights by participant characteristics. Analysis was based on first survey submission per survey type by a participant in which all trade-off questions and post-survey were complete. Models were adjusted for years of experience, language and WHO region of survey, field of expertise, eligibility for Gavi support and World Bank group classifications for country of work, type of work organisation, and participant's assessment regarding ease or difficulty to understand survey. Criteria weights are expressed as a percentage. Bars on plot indicate 95% confidence interval.

Abbreviations: MD, Mean Difference; CI, Confidence Interval; SD, Standard Deviation; No., Number of Participants

Characteristic	No. (%)	Mean (SD)	Adjusted MD (95% CI)
Disruption due to outbreaks			
English	*189 (69)*	*11.9 (6.3)*	*Reference*
Arabic	2 (1)	16.6 (0.8)	4.70 (-5.09 to 14.49)
Chinese	38 (14)	13.8 (8.5)	3.56 (-0.97 to 8.08)
French	*19 (7)*	*12.0 (6.0)*	*0.24 (-3.36 to 3.85)*
Portuguese	1 (0)	3.6 (0.0)	-10.76 (-24.38 to 2.86)
Russian	3 (1)	6.9 (3.0)	-2.58 (-11.32 to 6.16)
Spanish	*21 (8)*	*11.9 (6.0)*	*-0.54 (-5.43 to 4.35)*
Contribution to inequity			
English	189 (69)	11.2 (7.3)	Reference
Arabic	*2 (1)*	*10.7 (5.8)*	*-3.68 (-14.16 to 6.81)*
Chinese	38 (14)	11.1 (7.2)	-0.15 (-5.00 to 4.69)
French	19 (7)	11.9 (6.8)	-0.71 (-4.57 to 3.15)
Portuguese	*1 (0)*	*9.1 (-)*	*3.52 (-11.07 to 18.10)*
Russian	3 (1)	10.4 (3.2)	5.67 (-3.69 to 15.03)
Spanish	21 (8)	11.2 (6.2)	2.48 (-2.76 to 7.72)
Contribution to antimicrobial resistance			
English	189 (69)	13.8 (7.0)	Reference
Arabic	2 (1)	23.2 (12.5)	10.23 (-0.46 to 20.92)
Chinese	*38 (14)*	*11.8 (7.0)*	*1.76 (-3.18 to 6.70)*
French	19 (7)	15.8 (6.9)	0.96 (-2.98 to 4.89)
Portuguese	1 (0)	16.4 (0.0)	1.27 (-13.60 to 16.14)
Russian	*3 (1)*	*16.0 (12.2)*	*2.92 (-6.63 to 12.47)*
Spanish	21 (8)	12.8 (6.3)	1.44 (-3.90 to 6.78)
Unmet needs for prevention & treatment			
English	*189 (69)*	*12.2 (6.5)*	*Reference*
Arabic	2 (1)	12.9 (4.4)	-0.05 (-9.95 to 9.84)
Chinese	38 (14)	11.9 (7.5)	-4.50 (-9.08 to 0.07)
French	19 (7)	9.4 (6.5)	-3.31 (-6.96 to 0.34)
Portuguese	1 (0)	21.8 (-)	9.64 (-4.12 to 23.41)
Russian	3 (1)	8.9 (7.0)	-5.08 (-13.92 to 3.76)
Spanish	21 (8)	9.4 (5.2)	-4.58 (-9.53 to 0.36)

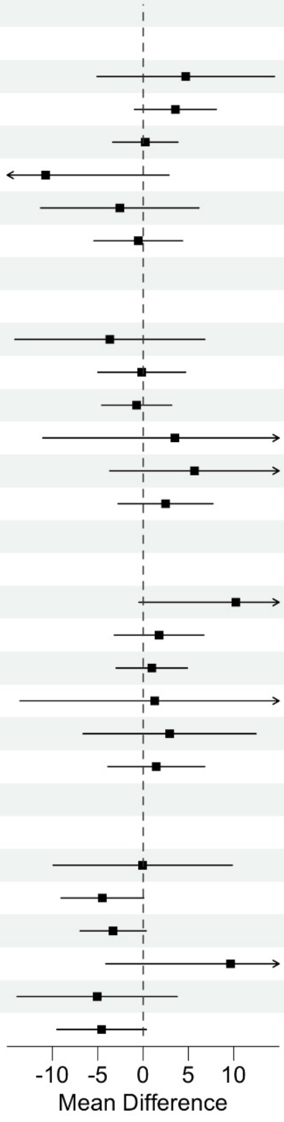

sFigure 14 Adjusted mean difference in criteria weights by participant characteristics: Years of experience (n=273)

Multivariate linear regression was used to compare mean differences in criteria weights by participant characteristics. Analysis was based on first survey submission per survey type by a participant in which all trade-off questions and post-survey were complete. Models were adjusted for years of experience, language and WHO region of survey, field of expertise, eligibility for Gavi support and World Bank group classifications for country of work, type of work organisation, and participant's assessment regarding ease or difficulty to understand survey. Criteria weights are expressed as a percentage. Bars on plot indicate 95% confidence interval.

Abbreviations: MD, Mean Difference; CI, Confidence Interval; SD, Standard Deviation; No., Number of Participants

Characteristic	No. (%)	Mean (SD)	Adjusted MD (95% CI)
Annual deaths in children under 5 years			
Up to 10 years	32 (12)	15.3 (9.4)	Reference
11-20 years	89 (33)	15.2 (8.9)	0.32 (-3.42 to 4.05)
21-30 years	73 (27)	15.8 (7.8)	0.73 (-3.08 to 4.54)
More than 30 years	79 (29)	14.3 (8.1)	-1.10 (-4.91 to 2.71)
Annual deaths in people 5 years & older			
Up to 10 years	32 (12)	13.7 (9.2)	Reference
11-20 years	89 (33)	13.1 (6.6)	-0.21 (-3.16 to 2.74)
21-30 years	73 (27)	12.7 (6.0)	-0.77 (-3.78 to 2.24)
More than 30 years	79 (29)	13.7 (6.6)	0.08 (-2.93 to 3.09)
Years lived with disability (YLD)			
Up to 10 years	32 (12)	12.2 (5.7)	Reference
11-20 years	89 (33)	12.9 (6.0)	0.68 (-1.79 to 3.14)
21-30 years	73 (27)	11.6 (5.3)	-0.66 (-3.18 to 1.86)
More than 30 years	79 (29)	12.5 (5.8)	0.33 (-2.19 to 2.85)
Social and economic burden per case			
Up to 10 years	32 (12)	11.4 (4.8)	Reference
11-20 years	89 (33)	10.5 (6.0)	-0.30 (-2.68 to 2.08)
21-30 years	73 (27)	9.4 (5.1)	-1.09 (-3.53 to 1.34)
More than 30 years	79 (29)	11.1 (5.2)	0.45 (-1.99 to 2.88)
Disruption due to outbreaks			
Up to 10 years	32 (12)	10.1 (6.0)	Reference
11-20 years	89 (33)	11.9 (6.0)	1.18 (-1.62 to 3.97)
21-30 years	73 (27)	12.8 (7.8)	2.33 (-0.52 to 5.18)
More than 30 years	79 (29)	12.7 (6.2)	2.41 (-0.44 to 5.26)
Contribution to inequity			
Up to 10 years	32 (12)	10.1 (5.9)	Reference
11-20 years	89 (33)	10.7 (5.9)	-0.22 (-3.22 to 2.77)
21-30 years	73 (27)	12.3 (8.3)	2.06 (-0.99 to 5.12)
More than 30 years	79 (29)	11.3 (7.4)	1.05 (-2.01 to 4.10)
Contribution to antimicrobial resistance			
Up to 10 years	32 (12)	14.9 (7.9)	Reference
11-20 years	89 (33)	13.3 (7.0)	-1.90 (-4.96 to 1.15)
21-30 years	73 (27)	13.8 (6.6)	-1.99 (-5.11 to 1.13)
More than 30 years	79 (29)	13.6 (7.3)	-2.08 (-5.20 to 1.03)
Unmet needs for prevention & treatment			
Up to 10 years	32 (12)	12.3 (7.9)	Reference
11-20 years	89 (33)	12.4 (6.8)	0.47 (-2.36 to 3.29)
21-30 years	73 (27)	11.6 (6.2)	-0.60 (-3.49 to 2.28)
More than 30 years	79 (29)	10.9 (6.1)	-1.13 (-4.02 to 1.75)

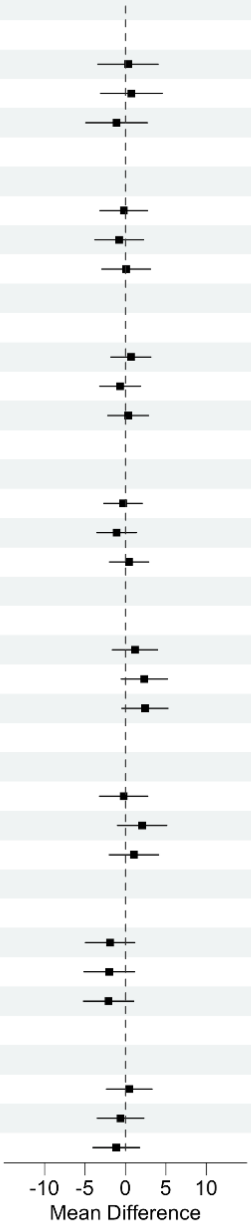

sFigure 15 Adjusted mean difference in criteria weights by participant characteristics: Areas of expertise and Annual deaths in children under 5 years (n=273)

Multivariate linear regression was used to compare mean differences in criteria weights by participant characteristics. Analysis was based on first survey submission per survey type by a participant in which all trade-off questions and post-survey were complete. Models were adjusted for years of experience, language and WHO region of survey, field of expertise, eligibility for Gavi support and World Bank group classifications for country of work, type of work organisation, and participant's assessment regarding ease or difficulty to understand survey. Criteria weights are expressed as a percentage. Bars on plot indicate 95% confidence interval.

Abbreviations: MD, Mean Difference; CI, Confidence Interval; SD, Standard Deviation; No., Number of Participants; Econ, Economics; R&D, Research and Development

Characteristic	No. (%)	Mean (SD)	Adjusted MD (95% CI)
Annual deaths in children under 5 years			
Disease epidemiology			
No	130 (48)	14.5 (8.0)	Reference
Yes	143 (52)	15.7 (8.8)	0.31 (-2.06 to 2.68)
Econ & health financing			
No	254 (93)	15.0 (8.3)	Reference
Yes	19 (7)	15.9 (10.1)	1.32 (-3.33 to 5.96)
Health care			
No	163 (60)	15.0 (8.8)	Reference
Yes	110 (40)	15.2 (7.9)	-0.01 (-2.57 to 2.55)
Health policy			
No	193 (71)	15.2 (8.5)	Reference
Yes	80 (29)	15.0 (8.3)	-0.71 (-3.39 to 1.97)
Regulatory affairs			
No	253 (93)	15.3 (8.6)	Reference
Yes	20 (7)	12.4 (5.6)	-4.17 (-9.66 to 1.31)
Vaccine R&D			
No	150 (55)	14.6 (7.8)	Reference
Yes	123 (45)	15.7 (9.1)	0.41 (-2.22 to 3.04)
Other			
No	242 (89)	15.5 (8.6)	Reference
Yes	31 (11)	12.1 (5.9)	-3.17 (-6.70 to 0.36)

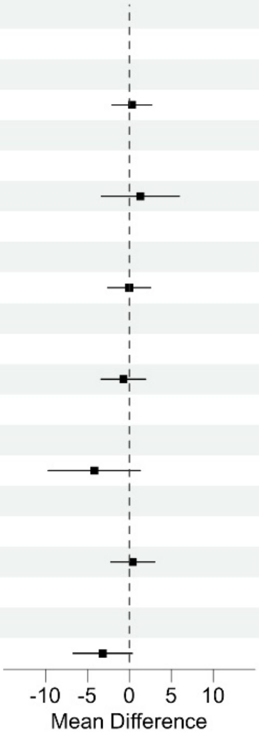

sFigure 16 Adjusted mean difference in criteria weights by participant characteristics: Areas of expertise and Annual deaths in people 5 years and older (n=273)

Multivariate linear regression was used to compare mean differences in criteria weights by participant characteristics. Analysis was based on first survey submission per survey type by a participant in which all trade-off questions and post-survey were complete. Models were adjusted for years of experience, language and WHO region of survey, field of expertise, eligibility for Gavi support and World Bank group classifications for country of work, type of work organisation, and participant's assessment regarding ease or difficulty to understand survey. Criteria weights are expressed as a percentage. Bars on plot indicate 95% confidence interval.

Abbreviations: MD, Mean Difference; CI, Confidence Interval; SD, Standard Deviation; No., Number of Participants; Econ, Economics; R&D, Research and Development

Characteristic	No. (%)	Mean (SD)	Adjusted MD (95% CI)
Annual deaths in people 5 years & older			
Disease epidemiology			
No	130 (48)	13.4 (6.6)	Reference
Yes	143 (52)	13.1 (6.9)	-1.10 (-2.97 to 0.77)
Econ & health financing			
No	254 (93)	13.1 (6.4)	Reference
Yes	19 (7)	15.4 (10.7)	3.02 (-0.65 to 6.70)
Health care			
No	163 (60)	13.6 (7.4)	Reference
Yes	110 (40)	12.8 (5.7)	-0.13 (-2.15 to 1.90)
Health policy			
No	193 (71)	13.0 (6.4)	Reference
Yes	80 (29)	13.7 (7.7)	1.09 (-1.02 to 3.21)
Regulatory affairs			
No	253 (93)	13.4 (6.9)	Reference
Yes	20 (7)	11.2 (5.4)	-3.56 (-7.90 to 0.77)
Vaccine R&D			
No	150 (55)	12.8 (7.0)	Reference
Yes	123 (45)	13.7 (6.5)	0.71 (-1.37 to 2.78)
Other			
No	242 (89)	13.3 (6.8)	Reference
Yes	31 (11)	12.7 (6.5)	-0.10 (-2.89 to 2.69)

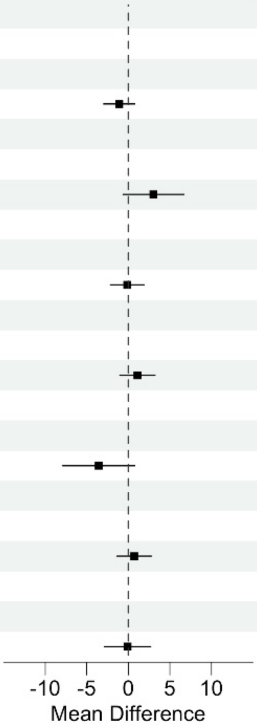

sFigure 17 Adjusted mean difference in criteria weights by participant characteristics: Areas of expertise and Years lived with disability (n=273)

Multivariate linear regression was used to compare mean differences in criteria weights by participant characteristics. Analysis was based on first survey submission per survey type by a participant in which all trade-off questions and post-survey were complete. Models were adjusted for years of experience, language and WHO region of survey, field of expertise, eligibility for Gavi support and World Bank group classifications for country of work, type of work organisation, and participant's assessment regarding ease or difficulty to understand survey. Criteria weights are expressed as a percentage. Bars on plot indicate 95% confidence interval.

Abbreviations: MD, Mean Difference; CI, Confidence Interval; SD, Standard Deviation; No., Number of Participants; Econ, Economics; R&D, Research and Development

Characteristic	No. (%)	Mean (SD)	Adjusted MD (95% CI)
Years lived with disability (YLD)			
Disease epidemiology			
No	130 (48)	12.0 (5.5)	Reference
Yes	143 (52)	12.0 (5.5)	-1.09 (-2.65 to 0.48)
Econ & health financing			
No	254 (93)	12.4 (5.8)	Reference
Yes	19 (7)	11.1 (4.4)	-0.57 (-3.65 to 2.50)
Health care			
No	163 (60)	12.7 (5.7)	Reference
Yes	110 (40)	11.9 (5.8)	-1.50 (-3.19 to 0.19)
Health policy			
No	193 (71)	12.3 (5.7)	Reference
Yes	80 (29)	12.4 (5.8)	0.75 (-1.02 to 2.53)
Regulatory affairs			
No	253 (93)	12.4 (5.7)	Reference
Yes	20 (7)	12.3 (6.2)	-0.60 (-4.22 to 3.03)
Vaccine R&D			
No	150 (55)	12.3 (5.9)	Reference
Yes	123 (45)	12.4 (5.9)	0.07 (-1.67 to 1.81)
Other			
No	242 (89)	12.4 (6.4)	Reference
Yes	31 (11)	12.4 (6.4)	-0.01 (-2.35 to 2.32)

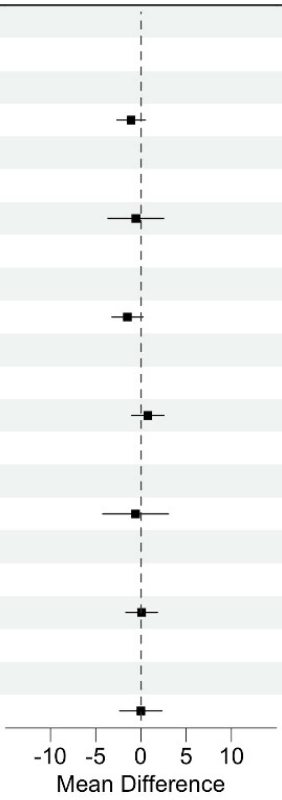

sFigure 18 Adjusted mean difference in criteria weights by participant characteristics: Areas of expertise and Social and economic burden per case (n=273)

Multivariate linear regression was used to compare mean differences in criteria weights by participant characteristics. Analysis was based on first survey submission per survey type by a participant in which all trade-off questions and post-survey were complete. Models were adjusted for years of experience, language and WHO region of survey, field of expertise, eligibility for Gavi support and World Bank group classifications for country of work, type of work organisation, and participant's assessment regarding ease or difficulty to understand survey. Criteria weights are expressed as a percentage. Bars on plot indicate 95% confidence interval.

Abbreviations: MD, Mean Difference; CI, Confidence Interval; SD, Standard Deviation; No., Number of Participants; Econ, Economics; R&D, Research and Development

Characteristic	No. (%)	Mean (SD)	Adjusted MD (95% CI)
Social and economic burden per case			
Disease epidemiology			
No	130 (48)	10.4 (5.3)	Reference
Yes	143 (52)	10.6 (5.5)	-0.13 (-1.64 to 1.38)
Econ & health financing			
No	254 (93)	10.3 (5.2)	Reference
Yes	19 (7)	12.5 (7.5)	0.51 (-2.46 to 3.48)
Health care			
No	163 (60)	10.7(5.5)	Reference
Yes	110 (40)	10.2 (5.3)	-0.42 (-2.05 to 1.22)
Health policy			
No	193 (71)	10.2 (5.2)	Reference
Yes	80 (29)	11.3 (5.9)	1.23 (-0.48 to 2.94)
Regulatory affairs			
No	253 (93)	10.5(5.4)	Reference
Yes	20 (7)	10.7 (6.2)	-0.46 (-3.97 to 3.04)
Vaccine R&D			
No	150 (55)	10.6 (5.6)	Reference
Yes	123 (45)	10.4 (5.2)	-0.04 (-1.71 to 1.64)
Other			
No	242 (89)	10.6 (5.0)	Reference
Yes	31 (11)	9.4 (5.0)	-1.02 (-3.28 to 1.23)

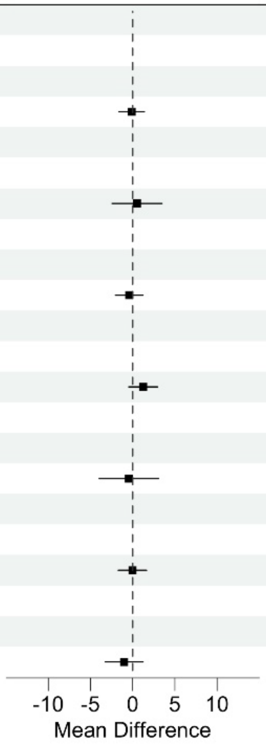

sFigure 19 Adjusted mean difference in criteria weights by participant characteristics: Areas of expertise and Disruption due to outbreaks (n=273)

Multivariate linear regression was used to compare mean differences in criteria weights by participant characteristics. Analysis was based on first survey submission per survey type by a participant in which all trade-off questions and post-survey were complete. Models were adjusted for years of experience, language and WHO region of survey, field of expertise, eligibility for Gavi support and World Bank group classifications for country of work, type of work organisation, and participant's assessment regarding ease or difficulty to understand survey. Criteria weights are expressed as a percentage. Bars on plot indicate 95% confidence interval.

Abbreviations: MD, Mean Difference; CI, Confidence Interval; SD, Standard Deviation; No., Number of Participants; Econ, Economics; R&D, Research and Development

Characteristic	No. (%)	Mean (SD)	Adjusted MD (95% CI)
Disruption due to outbreaks			
Disease epidemiology			
No	130 (48)	12.2 (6.5)	Reference
Yes	143 (52)	12.1 (6.7)	-0.70 (-2.47 to 1.07)
Econ & health financing			
No	254 (93)	12.2 (6.6)	Reference
Yes	19 (7)	11.9 (6.9)	-0.74 (-4.23 to 2.74)
Health care			
No	163 (60)	12.0 (6.8)	Reference
Yes	110 (40)	12.4 (6.4)	-0.38 (-2.30 to 1.53)
Health policy			
No	193 (71)	12.2 (6.4)	Reference
Yes	80 (29)	12.0 (7.1)	-0.17 (-2.18 to 1.83)
Regulatory affairs			
No	253 (93)	11.9 (6.4)	Reference
Yes	20 (7)	15.2 (8.7)	5.53 (1.42 to 9.64)
Vaccine R&D			
No	150 (55)	12.8 (6.4)	Reference
Yes	123 (45)	11.3 (6.9)	-0.65 (-2.62 to 1.32)
Other			
No	242 (89)	12.0 (6.6)	Reference
Yes	31 (11)	13.2 (6.8)	0.89 (-1.75 to 3.54)

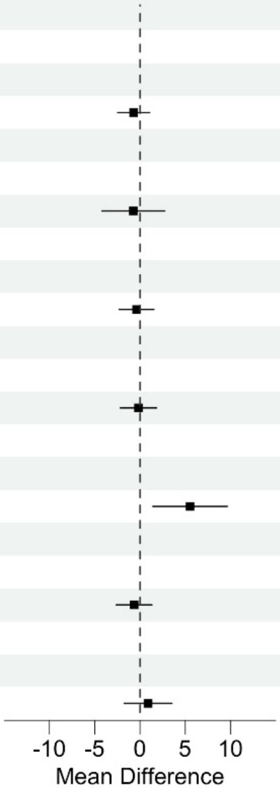

sFigure 20 Adjusted mean difference in criteria weights by participant characteristics: Areas of expertise and Contribution to inequity (n=273)

Multivariate linear regression was used to compare mean differences in criteria weights by participant characteristics. Analysis was based on first survey submission per survey type by a participant in which all trade-off questions and post-survey were complete. Models were adjusted for years of experience, language and WHO region of survey, field of expertise, eligibility for Gavi support and World Bank group classifications for country of work, type of work organisation, and participant's assessment regarding ease or difficulty to understand survey. Criteria weights are expressed as a percentage. Bars on plot indicate 95% confidence interval.

Abbreviations: MD, Mean Difference; CI, Confidence Interval; SD, Standard Deviation; No., Number of Participants; Econ, Economics; R&D, Research and Development

Characteristic	No. (%)	Mean (SD)	Adjusted MD (95% CI)
Contribution to inequity			
Disease epidemiology			
No	130 (48)	10.4 (7.5)	Reference
Yes	143 (52)	12.0 (7.5)	2.73 (0.83 to 4.63)
Econ & health financing			
No	254 (93)	11.3 (7.1)	Reference
Yes	19 (7)	9.8 (6.8)	-1.46 (-5.19 to 2.26)
Health care			
No	163 (60)	10.4 (6.7)	Reference
Yes	110 (40)	12.4 (7.4)	2.68 (0.62 to 4.73)
Health policy			
No	193 (71)	11.4 (6.9)	Reference
Yes	80 (29)	10.8 (6.9)	-1.37 (-3.51 to 0.78)
Regulatory affairs			
No	253 (93)	11.4 (7.1)	Reference
Yes	20 (7)	9.6 (6.4)	-1.68 (-6.08 to 2.72)
Vaccine R&D			
No	150 (55)	11.7 (7.2)	Reference
Yes	123 (45)	10.6 (6.8)	-1.29 (-3.40 to 0.82)
Other			
No	242 (89)	11.2 (7.2)	Reference
Yes	31 (11)	11.2 (6.2)	0.13 (-2.70 to 2.96)

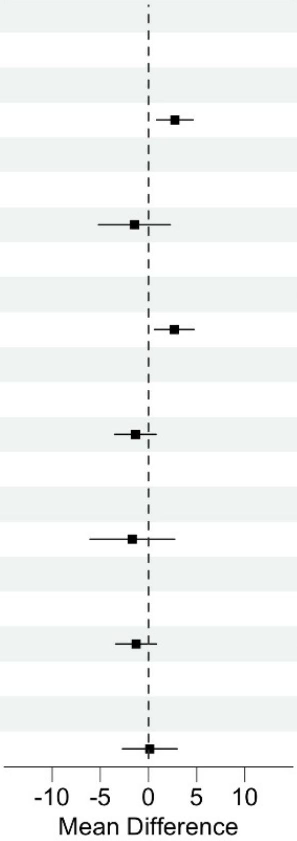

sFigure 21 Adjusted mean difference in criteria weights by participant characteristics: Areas of expertise and Contribution to antimicrobial resistance (n=273)

Multivariate linear regression was used to compare mean differences in criteria weights by participant characteristics. Analysis was based on first survey submission per survey type by a participant in which all trade-off questions and post-survey were complete. Models were adjusted for years of experience, language and WHO region of survey, field of expertise, eligibility for Gavi support and World Bank group classifications for country of work, type of work organisation, and participant's assessment regarding ease or difficulty to understand survey. Criteria weights are expressed as a percentage. Bars on plot indicate 95% confidence interval.

Abbreviations: MD, Mean Difference; CI, Confidence Interval; SD, Standard Deviation; No., Number of Participants; Econ, Economics; R&D, Research and Development

Characteristic	No. (%)	Mean (SD)	Adjusted MD (95% CI)
Contribution to antimicrobial resistance			
Disease epidemiology			
No	130 (48)	13.4 (7.4)	Reference
Yes	143 (52)	13.5 (6.8)	-0.13 (-2.07 to 1.80)
Econ & health financing			
No	254 (93)	13.7 (7.2)	Reference
Yes	19 (7)	13.7 (6.2)	-0.30 (-4.10 to 3.50)
Health care			
No	163 (60)	13.6 (7.0)	Reference
Yes	110 (40)	13.8 (7.2)	-0.31 (-2.40 to 1.79)
Health policy			
No	193 (71)	13.5 (7.0)	Reference
Yes	80 (29)	14.2 (7.4)	0.69 (-1.50 to 2.88)
Regulatory affairs			
No	253 (93)	13.7 (7.1)	Reference
Yes	20 (7)	13.7 (7.3)	1.55 (-2.94 to 6.04)
Vaccine R&D			
No	150 (55)	14.0 (6.8)	Reference
Yes	123 (45)	13.3 (7.4)	-0.30 (-2.45 to 1.85)
Other			
No	242 (89)	13.4 (7.0)	Reference
Yes	31 (11)	16.4 (7.2)	2.30 (-0.59 to 5.19)

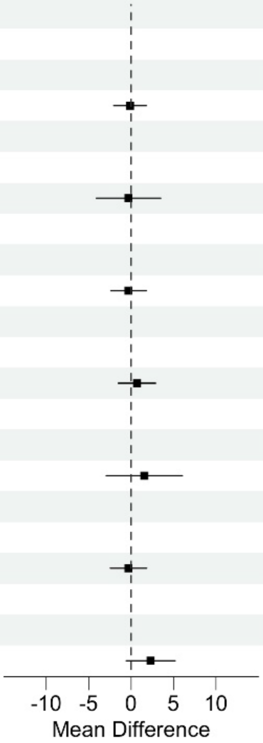

sFigure 22 Adjusted mean difference in criteria weights by participant characteristics: Areas of expertise and Unmet needs for prevention and treatment (n=273)

Multivariate linear regression was used to compare mean differences in criteria weights by participant characteristics. Analysis was based on first survey submission per survey type by a participant in which all trade-off questions and post-survey were complete. Models were adjusted for years of experience, language and WHO region of survey, field of expertise, eligibility for Gavi support and World Bank group classifications for country of work, type of work organisation, and participant's assessment regarding ease or difficulty to understand survey. Criteria weights are expressed as a percentage. Bars on plot indicate 95% confidence interval.

Abbreviations: MD, Mean Difference; CI, Confidence Interval; SD, Standard Deviation; No., Number of Participants; Econ, Economics; R&D, Research and Development

Characteristic	No. (%)	Mean (SD)	Adjusted MD (95% CI)
Unmet needs for prevention & treatment			
Disease epidemiology			
No	130 (48)	12.4 (6.8)	Reference
Yes	143 (52)	11.2 (6.4)	0.11 (-1.68 to 1.90)
Econ & health financing			
No	254 (93)	11.9 (6.6)	Reference
Yes	19 (7)	9.7 (6.0)	-1.76 (-5.28 to 1.75)
Health care			
No	163 (60)	12.0 (7.0)	Reference
Yes	110 (40)	11.3 (6.0)	0.07 (-1.87 to 2.01)
Health policy			
No	193 (71)	12.2 (6.6)	Reference
Yes	80 (29)	10.6 (6.3)	-1.52 (-3.55 to 0.50)
Regulatory affairs			
No	253 (93)	11.5 (6.4)	Reference
Yes	20 (7)	15.0 (7.7)	3.40 (-0.76 to 7.55)
Vaccine R&D			
No	150 (55)	11.1 (6.4)	Reference
Yes	123 (45)	12.5 (6.7)	1.10 (-0.89 to 3.09)
Other			
No	242 (89)	11.6 (6.5)	Reference
Yes	31 (11)	12.7 (7.3)	0.99 (-1.69 to 3.66)

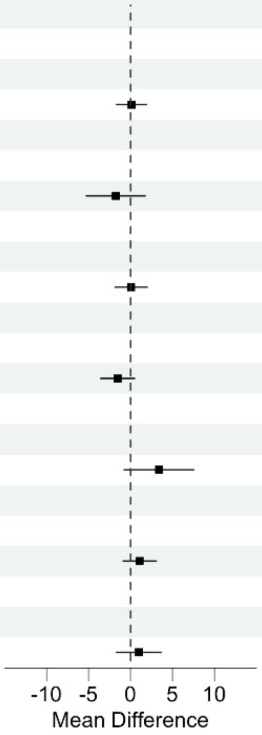

sFigure 23 Adjusted mean difference in criteria weights by participant characteristics: Type of work organisation and Annual deaths in children under 5 years (n=273)

Multivariate linear regression was used to compare mean differences in criteria weights by participant characteristics. Analysis was based on first survey submission per survey type by a participant in which all trade-off questions and post-survey were complete. Models were adjusted for years of experience, language and WHO region of survey, field of expertise, eligibility for Gavi support and World Bank group classifications for country of work, type of work organisation, and participant's assessment regarding ease or difficulty to understand survey. Criteria weights are expressed as a percentage. Bars on plot indicate 95% confidence interval.

Abbreviations: MD, Mean Difference; CI, Confidence Interval; SD, Standard Deviation; No., Number of Participants; NGO, Non-Governmental Organization

Characteristic	No. (%)	Mean (SD)	Adjusted MD (95% CI)
Annual deaths in children under 5 years			
Academic institution			
No	166 (61)	16.0 (7.8)	Reference
Yes	107 (39)	16.0 (9.2)	1.20 (-1.61 to 4.01)
Funding agency			
No	264 (97)	15.1 (8.4)	Reference
Yes	9 (3)	15.6 (8.6)	1.24 (-5.31 to 7.78)
Government			
No	183 (67)	15.0 (8.4)	Reference
Yes	90 (33)	15.4 (8.5)	0.49 (-2.31 to 3.28)
Health care provider			
No	223 (82)	16.0 (8.6)	Reference
Yes	50 (18)	16.0 (7.7)	0.06 (-3.21 to 3.33)
NGO			
No	248 (91)	15.2 (8.5)	Reference
Yes	25 (9)	13.6 (7.8)	-1.16 (-5.17 to 2.84)
Pharmaceutical industry			
No	251 (92)	13.4 (8.4)	Reference
Yes	22 (8)	13.4 (8.5)	-1.05 (-5.87 to 3.78)
Regulatory agency			
No	261 (96)	14.4 (8.4)	Reference
Yes	12 (4)	14.4 (8.5)	3.08 (-3.92 to 10.08)
United Nations agency			
No	239 (88)	15.0 (8.3)	Reference
Yes	34 (12)	15.5 (9.0)	0.59 (-3.56 to 4.74)
Other			
No	255 (93)	15.2 (8.6)	Reference
Yes	18 (7)	13.1 (5.3)	-0.93 (-5.55 to 3.70)

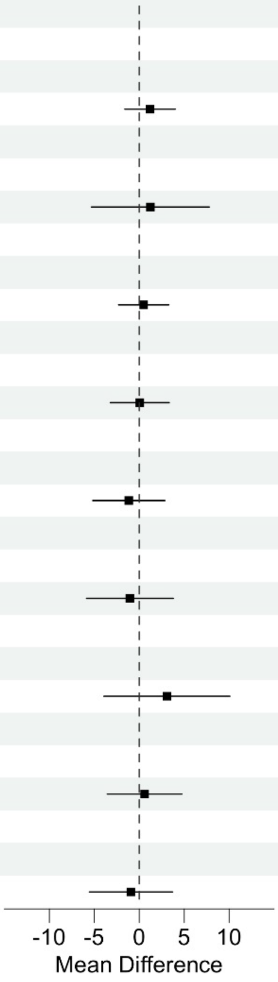

sFigure 24 Adjusted mean difference in criteria weights by participant characteristics: Type of work organisation and Annual deaths in people 5 years and older (n=273)

Multivariate linear regression was used to compare mean differences in criteria weights by participant characteristics. Analysis was based on first survey submission per survey type by a participant in which all trade-off questions and post-survey were complete. Models were adjusted for years of experience, language and WHO region of survey, field of expertise, eligibility for Gavi support and World Bank group classifications for country of work, type of work organisation, and participant's assessment regarding ease or difficulty to understand survey. Criteria weights are expressed as a percentage. Bars on plot indicate 95% confidence interval.

Abbreviations: MD, Mean Difference; CI, Confidence Interval; SD, Standard Deviation; No., Number of Participants; NGO, Non-Governmental Organization

Characteristic	No. (%)	Mean (SD)	Adjusted MD (95% CI)
Annual deaths in people 5 years & older			
Academic institution			
No	166 (61)	12.7 (6.4)	Reference
Yes	107 (39)	14.1 (7.3)	0.95 (-1.27 to 3.16)
Funding agency			
No	264 (97)	13.2 (6.8)	Reference
Yes	9 (3)	14.1 (7.2)	0.11 (-5.06 to 5.28)
Government			
No	183 (67)	13.7 (7.3)	Reference
Yes	90 (33)	12.3 (5.5)	-1.39 (-3.60 to 0.82)
Health care provider			
No	223 (82)	13.4 (7.0)	Reference
Yes	50 (18)	12.5 (5.9)	-0.50 (-3.09 to 2.08)
NGO			
No	248 (91)	13.6 (6.9)	Reference
Yes	25 (9)	9.8 (4.6)	-3.98 (-7.15 to -0.82)
Pharmaceutical industry			
No	251 (92)	13.2 (6.6)	Reference
Yes	22 (8)	13.8 (8.5)	-1.29 (-5.10 to 2.52)
Regulatory agency			
No	261 (96)	13.4 (6.8)	Reference
Yes	12 (4)	10.8 (5.6)	1.37 (-4.16 to 6.90)
United Nations agency			
No	239 (88)	14.7 (6.8)	Reference
Yes	34 (12)	14.7 (6.8)	1.69 (-1.59 to 4.96)
Other			
No	255 (93)	13.3 (6.8)	Reference
Yes	18 (7)	12.5 (6.3)	-0.58 (-4.23 to 3.08)

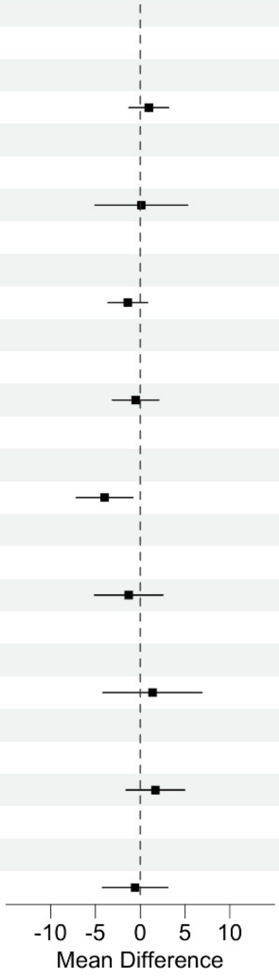

sFigure 25 Adjusted mean difference in criteria weights by participant characteristics: Type of work organisation and Years lived with disability (n=273)

Multivariate linear regression was used to compare mean differences in criteria weights by participant characteristics. Analysis was based on first survey submission per survey type by a participant in which all trade-off questions and post-survey were complete. Models were adjusted for years of experience, language and WHO region of survey, field of expertise, eligibility for Gavi support and World Bank group classifications for country of work, type of work organisation, and participant's assessment regarding ease or difficulty to understand survey. Criteria weights are expressed as a percentage. Bars on plot indicate 95% confidence interval.

Abbreviations: MD, Mean Difference; CI, Confidence Interval; SD, Standard Deviation; No., Number of Participants; NGO, Non-Governmental Organization

Characteristic	No. (%)	Mean (SD)	Adjusted MD (95% CI)
Years lived with disability (YLD)			
Academic institution			
No	166 (61)	12.4 (5.6)	Reference
Yes	107 (39)	12.3 (5.9)	-1.30 (-3.16 to 0.55)
Funding agency			
No	264 (97)	12.4 (5.8)	Reference
Yes	9 (3)	11.2 (4.3)	-2.75 (-7.08 to 1.58)
Government			
No	183 (67)	12.9 (5.8)	Reference
Yes	90 (33)	11.3 (5.4)	-2.41 (-4.26 to -0.56)
Health care provider			
No	223 (82)	12.0 (5.4)	Reference
Yes	50 (18)	13.9 (6.7)	2.21 (0.04 to 4.37)
NGO			
No	248 (91)	12.4 (5.8)	Reference
Yes	25 (9)	12.2 (4.9)	-0.99 (-3.64 to 1.67)
Pharmaceutical industry			
No	251 (92)	12.4 (6.8)	Reference
Yes	22 (8)	12.1 (6.8)	-2.23 (-5.42 to 0.96)
Regulatory agency			
No	261 (96)	12.3 (5.8)	Reference
Yes	12 (4)	13.2 (4.8)	0.85 (-3.78 to 5.48)
United Nations agency			
No	239 (88)	12.4 (5.8)	Reference
Yes	34 (12)	11.9 (5.5)	-2.13 (-4.88 to 0.61)
Other			
No	255 (93)	12.4 (5.8)	Reference
Yes	18 (7)	11.2 (4.4)	-1.77 (-4.83 to 1.29)

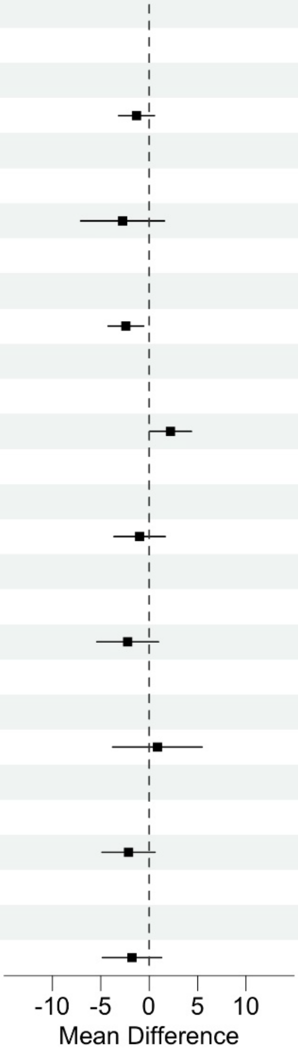

sFigure 26 Adjusted mean difference in criteria weights by participant characteristics: Type of work organisation and Social and economic burden per case (n=273)

Multivariate linear regression was used to compare mean differences in criteria weights by participant characteristics. Analysis was based on first survey submission per survey type by a participant in which all trade-off questions and post-survey were complete. Models were adjusted for years of experience, language and WHO region of survey, field of expertise, eligibility for Gavi support and World Bank group classifications for country of work, type of work organisation, and participant's assessment regarding ease or difficulty to understand survey. Criteria weights are expressed as a percentage. Bars on plot indicate 95% confidence interval.

Abbreviations: MD, Mean Difference; CI, Confidence Interval; SD, Standard Deviation; No., Number of Participants; NGO, Non-Governmental Organization

Characteristic	No. (%)	Mean (SD)	Adjusted MD (95% CI)
Social and economic burden per case			
Academic institution			
No	166 (61)	10.5 (5.5)	Reference
Yes	107 (39)	10.4 (5.3)	0.54 (-1.25 to 2.33)
Funding agency			
No	264 (97)	10.5 (5.4)	Reference
Yes	9 (3)	8.7 (5.8)	-2.19 (-6.37 to 1.99)
Government			
No	183 (67)	10.4 (5.5)	Reference
Yes	90 (33)	10.6 (5.2)	-0.04 (-1.82 to 1.75)
Health care provider			
No	223 (82)	10.7 (5.3)	Reference
Yes	50 (18)	9.7 (5.7)	-0.23 (-2.32 to 1.86)
NGO			
No	248 (91)	10.4 (5.3)	Reference
Yes	25 (9)	11.0 (6.4)	0.32 (-2.24 to 2.88)
Pharmaceutical industry			
No	251 (92)	10.6 (5.5)	Reference
Yes	22 (8)	9.4 (4.6)	-0.08 (-3.16 to 3.00)
Regulatory agency			
No	261 (96)	10.4 (5.4)	Reference
Yes	12 (4)	11.0 (5.8)	0.72 (-3.75 to 5.19)
United Nations agency			
No	239 (88)	10.3 (5.3)	Reference
Yes	34 (12)	11.5 (5.9)	0.80 (-1.85 to 3.45)
Other			
No	255 (93)	10.4 (5.4)	Reference
Yes	18 (7)	10.8 (5.3)	0.71 (-2.24 to 3.67)

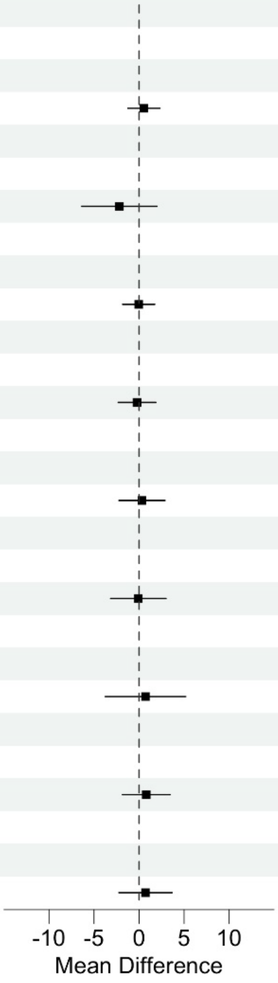

sFigure 27 Adjusted mean difference in criteria weights by participant characteristics: Type of work organisation and Disruption due to outbreaks (n=273)

Multivariate linear regression was used to compare mean differences in criteria weights by participant characteristics. Analysis was based on first survey submission per survey type by a participant in which all trade-off questions and post-survey were complete. Models were adjusted for years of experience, language and WHO region of survey, field of expertise, eligibility for Gavi support and World Bank group classifications for country of work, type of work organisation, and participant's assessment regarding ease or difficulty to understand survey. Criteria weights are expressed as a percentage. Bars on plot indicate 95% confidence interval.

Abbreviations: MD, Mean Difference; CI, Confidence Interval; SD, Standard Deviation; No., Number of Participants; NGO, Non-Governmental Organization

Characteristic	No. (%)	Mean (SD)	Adjusted MD (95% CI)
Disruption due to outbreaks			
Academic institution			
No	166 (61)	12.3 (6.5)	Reference
Yes	107 (39)	11.9 (6.8)	0.34 (-1.76 to 2.45)
Funding agency			
No	264 (97)	12.0 (6.6)	Reference
Yes	9 (3)	15.6 (6.7)	5.49 (0.59 to 10.39)
Government			
No	183 (67)	12.2 (6.7)	Reference
Yes	90 (33)	12.0 (6.5)	0.58 (-1.51 to 2.68)
Health care provider			
No	223 (82)	12.0 (6.4)	Reference
Yes	50 (18)	12.9 (7.6)	1.86 (-0.59 to 4.31)
NGO			
No	248 (91)	12.0 (6.6)	Reference
Yes	25 (9)	13.1 (6.4)	1.37 (-1.63 to 4.37)
Pharmaceutical industry			
No	251 (92)	12.2 (6.4)	Reference
Yes	22 (8)	11.2 (8.6)	-0.80 (-4.41 to 2.82)
Regulatory agency			
No	261 (96)	12.1 (6.7)	Reference
Yes	12 (4)	12.6 (5.7)	-4.65 (-9.89 to 0.60)
United Nations agency			
No	239 (88)	12.1 (6.5)	Reference
Yes	34 (12)	12.7 (7.6)	1.86 (-1.25 to 4.97)
Other			
No	255 (93)	12.1 (6.6)	Reference
Yes	18 (7)	13.3 (6.3)	1.01 (-2.45 to 4.48)

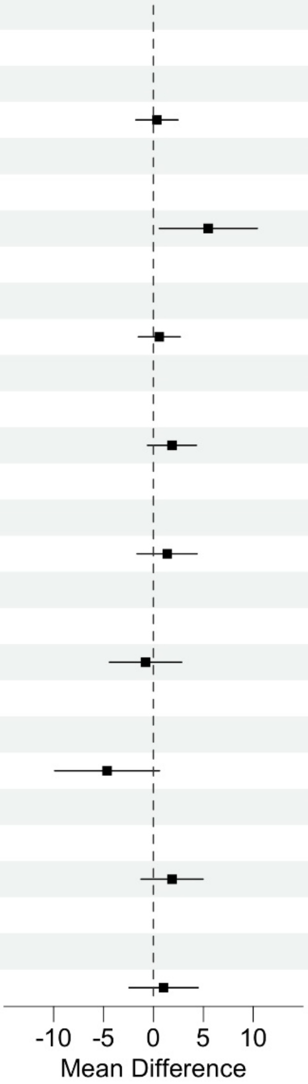

sFigure 28 Adjusted mean difference in criteria weights by participant characteristics: Type of work organisation and Contribution to inequity (n=273)

Multivariate linear regression was used to compare mean differences in criteria weights by participant characteristics. Analysis was based on first survey submission per survey type by a participant in which all trade-off questions and post-survey were complete. Models were adjusted for years of experience, language and WHO region of survey, field of expertise, eligibility for Gavi support and World Bank group classifications for country of work, type of work organisation, and participant's assessment regarding ease or difficulty to understand survey. Criteria weights are expressed as a percentage. Bars on plot indicate 95% confidence interval.

Abbreviations: MD, Mean Difference; CI, Confidence Interval; SD, Standard Deviation; No., Number of Participants; NGO, Non-Governmental Organization

Characteristic	No. (%)	Mean (SD)	Adjusted MD (95% CI)
Contribution to inequity			
Academic institution			
No	166 (61)	11.4 (7.2)	Reference
Yes	107 (39)	10.9 (6.8)	0.16 (-2.09 to 2.41)
Funding agency			
No	264 (97)	11.2 (7.1)	Reference
Yes	9 (3)	12.1 (4.7)	1.14 (-4.11 to 6.39)
Government			
No	183 (67)	10.6 (6.4)	Reference
Yes	90 (33)	12.5 (8.0)	2.15 (-0.10 to 4.39)
Health care provider			
No	223 (82)	11.4 (7.1)	Reference
Yes	50 (18)	10.4 (6.7)	-2.65 (-5.27 to -0.02)
NGO			
No	248 (91)	11.3 (7.0)	Reference
Yes	25 (9)	10.4 (7.7)	-1.13 (-4.35 to 2.08)
Pharmaceutical industry			
No	251 (92)	11.1 (7.0)	Reference
Yes	22 (8)	13.1 (6.9)	5.06 (1.19 to 8.93)
Regulatory agency			
No	261 (96)	11.3 (7.0)	Reference
Yes	12 (4)	9.7 (7.1)	0.07 (-5.55 to 5.68)
United Nations agency			
No	239 (88)	11.2 (7.0)	Reference
Yes	34 (12)	11.1 (7.7)	-0.23 (-3.56 to 3.10)
Other			
No	255 (93)	11.3 (7.2)	Reference
Yes	18 (7)	10.2 (5.0)	-0.87 (-4.58 to 2.84)

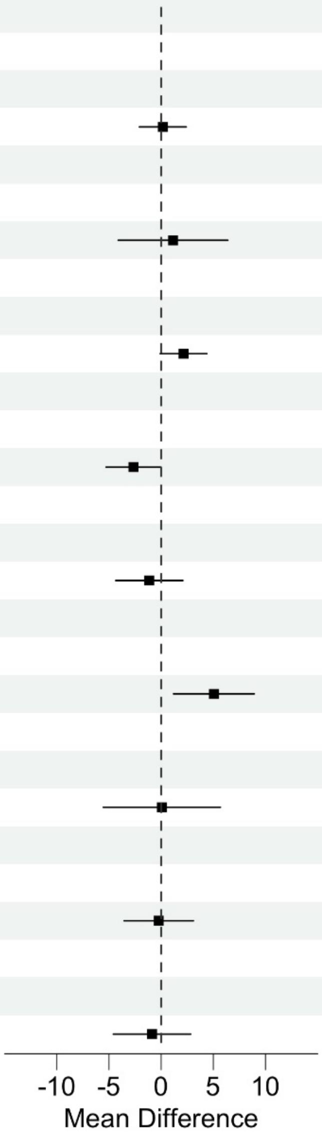

sFigure 29 Adjusted mean difference in criteria weights by participant characteristics: Type of work organisation and Contribution to antimicrobial resistance (n=273)

Multivariate linear regression was used to compare mean differences in criteria weights by participant characteristics. Analysis was based on first survey submission per survey type by a participant in which all trade-off questions and post-survey were complete. Models were adjusted for years of experience, language and WHO region of survey, field of expertise, eligibility for Gavi support and World Bank group classifications for country of work, type of work organisation, and participant's assessment regarding ease or difficulty to understand survey. Criteria weights are expressed as a percentage. Bars on plot indicate 95% confidence interval.

Abbreviations: MD, Mean Difference; CI, Confidence Interval; SD, Standard Deviation; No., Number of Participants; NGO, Non-Governmental Organization

Characteristic	No. (%)	Mean (SD)	Adjusted MD (95% CI)
Contribution to antimicrobial resistance			
Academic institution			
No	166 (61)	14.3 (7.0)	Reference
Yes	107 (39)	12.8 (7.1)	-1.54 (-3.83 to 0.76)
Funding agency			
No	264 (97)	13.7 (7.0)	Reference
Yes	9 (3)	12.9 (8.6)	-0.33 (-5.68 to 5.02)
Government			
No	183 (67)	13.2 (7.3)	Reference
Yes	90 (33)	14.7 (6.5)	1.01 (-1.27 to 3.30)
Health care provider			
No	223 (82)	13.8 (7.1)	Reference
Yes	50 (18)	13.5 (7.0)	-0.14 (-2.82 to 2.53)
NGO			
No	248 (91)	13.5 (7.0)	Reference
Yes	25 (9)	16.0 (7.6)	2.86 (-0.42 to 6.14)
Pharmaceutical industry			
No	251 (92)	13.6 (6.9)	Reference
Yes	22 (8)	14.9 (8.6)	1.31 (-2.63 to 5.26)
Regulatory agency			
No	261 (96)	13.7 (7.1)	Reference
Yes	12 (4)	13.6 (7.7)	-2.12 (-7.84 to 3.61)
United Nations agency			
No	239 (88)	14.0 (7.1)	Reference
Yes	34 (12)	11.9 (6.8)	-1.11 (-4.51 to 2.28)
Other			
No	255 (93)	13.6 (7.1)	Reference
Yes	18 (7)	14.5 (6.3)	0.35 (-3.43 to 4.13)

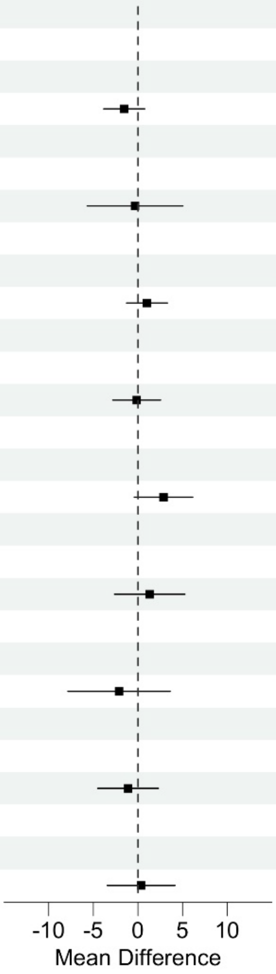

sFigure 30 Adjusted mean difference in criteria weights by participant characteristics: Type of work organisation and Unmet needs for prevention and treatment (n=273)

Multivariate linear regression was used to compare mean differences in criteria weights by participant characteristics. Analysis was based on first survey submission per survey type by a participant in which all trade-off questions and post-survey were complete. Models were adjusted for years of experience, language and WHO region of survey, field of expertise, eligibility for Gavi support and World Bank group classifications for country of work, type of work organisation, and participant's assessment regarding ease or difficulty to understand survey. Criteria weights are expressed as a percentage. Bars on plot indicate 95% confidence interval.

Abbreviations: MD, Mean Difference; CI, Confidence Interval; SD, Standard Deviation; No., Number of Participants; NGO, Non-Governmental Organization

Characteristic	No. (%)	Mean (SD)	Adjusted MD (95% CI)
Unmet needs for prevention & treatment			
Academic institution			
No	166 (61)	11.9 (6.7)	Reference
Yes	107 (39)	11.5 (6.4)	-0.35 (-2.48 to 1.77)
Funding agency			
No	264 (97)	11.8 (6.6)	Reference
Yes	9 (3)	9.8 (4.7)	-2.72 (-7.67 to 2.24)
Government			
No	183 (67)	12.0 (6.8)	Reference
Yes	90 (33)	11.2 (6.2)	-0.39 (-2.51 to 1.72)
Health care provider			
No	223 (82)	11.9 (6.8)	Reference
Yes	50 (18)	11.0 (5.5)	-0.60 (-3.08 to 1.88)
NGO			
No	248 (91)	11.5 (6.4)	Reference
Yes	25 (9)	13.8 (7.7)	2.72 (-0.32 to 5.75)
Pharmaceutical industry			
No	251 (92)	11.7 (6.6)	Reference
Yes	22 (8)	12.2 (6.5)	-0.94 (-4.59 to 2.72)
Regulatory agency			
No	261 (96)	11.6 (6.5)	Reference
Yes	12 (4)	14.7 (8.3)	0.68 (-4.62 to 5.99)
United Nations agency			
No	239 (88)	11.9 (6.5)	Reference
Yes	34 (12)	10.7 (7.2)	-1.46 (-4.60 to 1.69)
Other			
No	255 (93)	11.5 (6.5)	Reference
Yes	18 (7)	14.4 (7.0)	2.07 (-1.43 to 5.58)

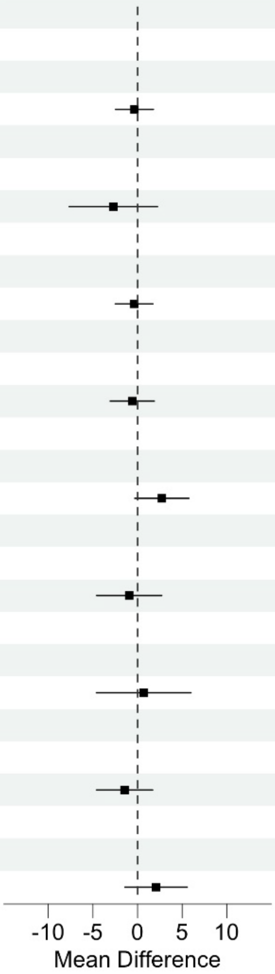

sFigure 31 Adjusted mean difference in criteria weights by participant characteristics: Gavi eligibility of participants' country of work (n=273)

Multivariate linear regression was used to compare mean differences in criteria weights by participant characteristics. Analysis was based on first survey submission per survey type by a participant in which all trade-off questions and post-survey were complete. Models were adjusted for years of experience, language and WHO region of survey, field of expertise, eligibility for Gavi support and World Bank group classifications for country of work, type of work organisation, and participant's assessment regarding ease or difficulty to understand survey. Criteria weights are expressed as a percentage. Bars on plot indicate 95% confidence interval.

Abbreviations: MD, Mean Difference; CI, Confidence Interval; SD, Standard Deviation; No., Number of Participants

Characteristic	No. (%)	Mean (SD)	Adjusted MD (95% CI)
Annual deaths in children under 5 years			
Not eligible	*205 (75)*	*15.4 (8.5)*	*Reference*
Eligible	68 (25)	14.2 (8.0)	1.02 (-3.78 to 5.83)
Annual deaths in people 5 years & older			
Not eligible	*205 (75)*	*13.7 (6.8)*	*Reference*
Eligible	68 (25)	11.8 (6.7)	0.35 (-3.45 to 4.15)
Years lived with disability (YLD)			
Not eligible	*205 (75)*	*12.6 (5.9)*	*Reference*
Eligible	68 (25)	11.6 (5.0)	1.46 (-1.72 to 4.64)
Social and economic burden per case			
Not eligible	*205 (75)*	*10.2 (5.4)*	*Reference*
Eligible	68 (25)	11.2 (5.4)	-1.97 (-5.04 to 1.10)
Disruption due to outbreaks			
Not eligible	*205 (75)*	*12.0 (6.8)*	*Reference*
Eligible	68 (25)	12.6 (6.1)	-2.03 (-5.63 to 1.57)
Contribution to inequity			
Not eligible	*205 (75)*	*10.8 (7.0)*	*Reference*
Eligible	68 (25)	12.4 (7.0)	3.60 (-0.26 to 7.45)
Contribution to antimicrobial resistance			
Not eligible	*205 (75)*	*13.7 (7.3)*	*Reference*
Eligible	68 (25)	13.8 (6.6)	-3.04 (-6.97 to 0.89)
Unmet needs for prevention & treatment			
Not eligible	*205 (75)*	*11.6 (6.4)*	*Reference*
Eligible	68 (25)	12.3 (7.2)	0.61 (-3.03 to 4.25)

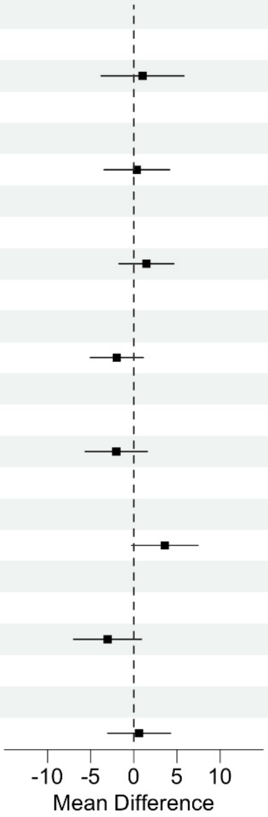

sFigure 32 Adjusted mean difference in criteria weights by participant characteristics: World Bank classification of participants' country of work (n=273)

Multivariate linear regression was used to compare mean differences in criteria weights by participant characteristics. Analysis was based on first survey submission per survey type by a participant in which all trade-off questions and post-survey were complete. Models were adjusted for years of experience, language and WHO region of survey, field of expertise, eligibility for Gavi support and World Bank group classifications for country of work, type of work organisation, and participant's assessment regarding ease or difficulty to understand survey. Criteria weights are expressed as a percentage. Bars on plot indicate 95% confidence interval.

Abbreviations: MD, Mean Difference; CI, Confidence Interval; SD, Standard Deviation; No., Number of Participants

Characteristic	No. (%)	Mean (SD)	Adjusted MD (95% CI)
Annual deaths in children under 5 years			
High income	68 (25)	16.3 (9.0)	Reference
Upper middle income	85 (31)	15.5 (8.4)	-0.82 (-5.75 to 4.11)
Lower middle income	90 (33)	14.3 (8.1)	-1.35 (-6.29 to 3.60)
Low income	30 (11)	13.6 (8.0)	-2.74 (-9.39 to 3.90)
Annual deaths in people 5 years & older			
High income	68 (25)	14.2 (6.8)	Reference
Upper middle income	85 (31)	13.9 (7.5)	-1.40 (-5.30 to 2.49)
Lower middle income	90 (33)	12.7 (6.1)	-1.55 (-5.46 to 2.36)
Low income	30 (11)	10.9 (5.9)	-3.42 (-8.67 to 1.83)
Years lived with disability (YLD)			
High income	68 (25)	13.7 (6.5)	Reference
Upper middle income	85 (31)	12.6 (5.8)	0.14 (-3.13 to 3.40)
Lower middle income	90 (33)	11.6 (5.1)	-0.90 (-4.17 to 2.37)
Low income	30 (11)	11.0 (5.2)	-1.82 (-6.22 to 2.57)
Social and economic burden per case			
High income	68 (25)	9.4 (4.9)	Reference
Upper middle income	85 (31)	10.2 (5.9)	0.66 (-2.49 to 3.81)
Lower middle income	90 (33)	10.9 (4.8)	0.36 (-2.80 to 3.52)
Low income	30 (11)	12.5 (6.0)	2.34 (-1.90 to 6.59)

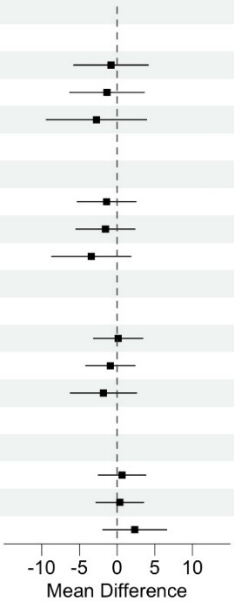

Characteristic	No. (%)	Mean (SD)	Adjusted MD (95% CI)
Disruption due to outbreaks			
High income	68 (25)	9.8 (5.6)	Reference
Upper middle income	85 (31)	12.9 (7.0)	0.49 (-3.20 to 4.18)
Lower middle income	90 (33)	13.0 (7.0)	1.09 (-2.62 to 4.79)
Low income	30 (11)	13.0 (5.2)	1.33 (-3.65 to 6.31)
Contribution to inequity			
High income	68 (25)	10.6 (7.9)	Reference
Upper middle income	85 (31)	10.7 (6.5)	-0.54 (-4.50 to 3.41)
Lower middle income	90 (33)	12.0 (6.7)	-1.21 (-5.18 to 2.75)
Low income	30 (11)	12.1 (7.3)	-0.58 (-5.91 to 4.75)
Contribution to antimicrobial resistance			
High income	68 (25)	14.5 (8.1)	Reference
Upper middle income	85 (31)	12.3 (6.3)	-2.08 (-6.11 to 1.95)
Lower middle income	90 (33)	14.0 (6.7)	1.75 (-2.30 to 5.79)
Low income	30 (11)	14.9 (7.4)	2.61 (-2.83 to 8.04)
Unmet needs for prevention & treatment			
High income	68 (25)	11.5 (6.2)	Reference
Upper middle income	85 (31)	11.9 (6.9)	3.55 (-0.18 to 7.28)
Lower middle income	90 (33)	11.6 (6.6)	1.82 (-1.92 to 5.56)
Low income	30 (11)	12.0 (6.7)	2.28 (-2.75 to 7.32)

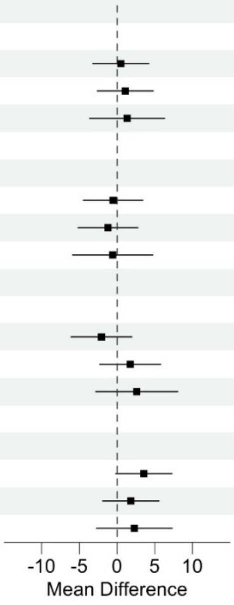

Characterising group patterns in criteria weights

sFigure 33 Criteria weights by cluster (2-cluster model) (n=273)

Error bars show 95% confidence interval.

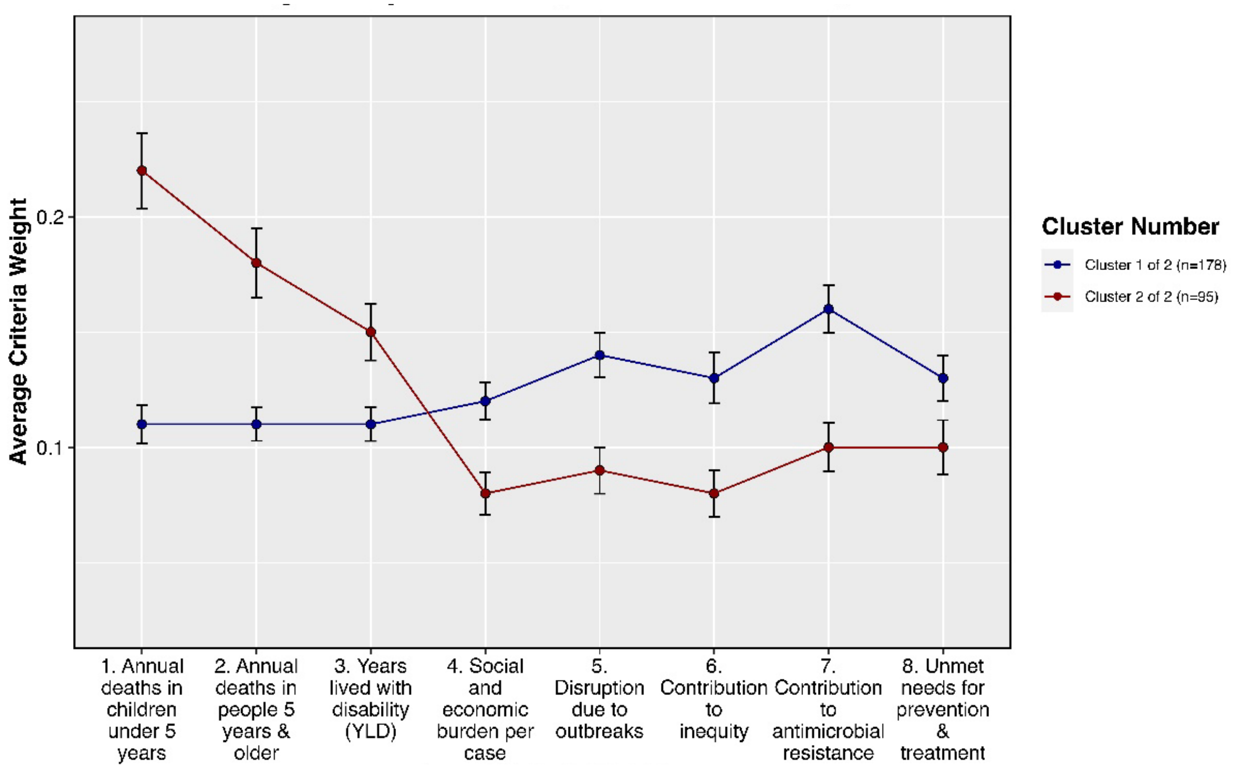

Assessing whether groups of respondents sharing similar patterns in criteria weights also shared similar biographical backgrounds or impressions of the survey

sFigure 34 Participant Factors associated with cluster membership, Years of experience and Areas of expertise

A generalized linear model was used to compare odds of membership in a specific cluster by participant characteristics. Cluster #1 and Cluster #2 columns represent number of subjects (%). Models were adjusted for years of experience, language and WHO region of survey, field of expertise, eligibility for Gavi support and World Bank group classifications for country of work, type of work organisation, and participant's assessment regarding ease or difficulty to understand survey. Bars on plot indicate 95% confidence interval.

Abbreviations: OR, Odds Ratio; CI, Confidence Interval

Characteristic	Cluster #1 (n=178)	Cluster #2 (n=95)	Adjusted OR (95% CI)
Years of Experience			
Up to 10 years	24 (14)	8 (8)	Reference
11-20 years	51 (29)	38 (40)	2.52 (0.93 to 7.36)
21-30 years	46 (26)	27 (28)	2.04 (0.73 to 6.06)
More than 30 years	57 (32)	22 (23)	0.99 (0.35 to 2.96)
Area of Expertise			
Disease epidemiology			
No	88 (49)	42 (44)	Reference
Yes	90 (51)	53 (56)	1.15 (0.61 to 2.15)
Econ & health financing			
No	165 (93)	89 (94)	Reference
Yes	13 (7)	6 (6)	0.90 (0.25 to 3.03)
Health care			
No	103 (58)	60 (63)	Reference
Yes	75 (42)	35 (37)	0.76 (0.38 to 1.49)
Health policy			
No	127 (71)	66 (70)	Reference
Yes	51 (29)	29 (30)	1.03 (0.50 to 2.09)
Regulatory affairs			
No	163 (92)	90 (95)	Reference
Yes	15 (8)	5 (5)	0.43 (0.08 to 1.86)
Vaccine R&D			
No	103 (58)	47 (50)	Reference
Yes	75 (42)	48 (50)	1.11 (0.56 to 2.21)
Other			
No	154 (86)	88 (93)	Reference
Yes	24 (14)	7 (7)	0.47 (0.16 to 1.25)

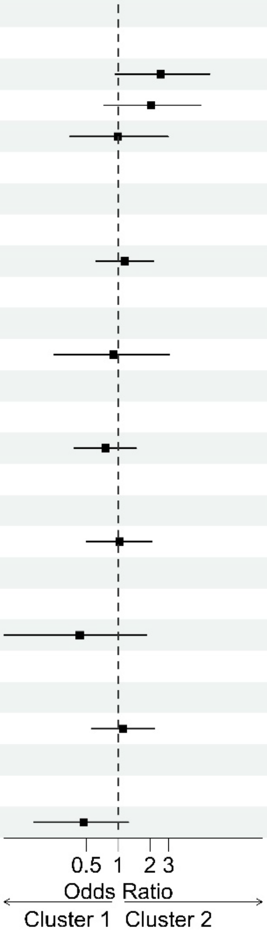

sFigure 35 Participant Factors associated with cluster membership, Type of work organization and Country of work

A generalized linear model was used to compare odds of membership in a specific cluster by participant characteristics. Cluster #1 and Cluster #2 columns represent number of subjects (%). Models were adjusted for years of experience, language and WHO region of survey, field of expertise, eligibility for Gavi support and World Bank group classifications for country of work, type of work organisation, and participant's assessment regarding ease or difficulty to understand survey. Bars on plot indicate 95% confidence interval.

Abbreviations: OR, Odds Ratio; CI, Confidence Interval; NGO, Non-Governmental Organization

Characteristic	Cluster #1 (n=178)	Cluster #2 (n=95)	Adjusted OR (95% CI)
Type of work organization			
Academic institution			
No	111 (62)	55 (58)	Reference
Yes	67 (38)	40 (42)	1.11 (0.51 to 2.39)
Funding agency			
No	173 (97)	91 (96)	Reference
Yes	5 (3)	4 (4)	1.64 (0.23 to 10.07)
Government			
No	113 (64)	70 (74)	Reference
Yes	65 (36)	25 (26)	0.53 (0.23 to 1.15)
Health care provider			
No	145 (82)	78 (82)	Reference
Yes	33 (18)	17 (18)	0.97 (0.39 to 2.36)
NGO			
No	157 (88)	91 (96)	Reference
Yes	21 (12)	4 (4)	0.27 (0.07 to 0.89)
Pharmaceutical industry			
No	165 (93)	86 (90)	Reference
Yes	13 (7)	9 (10)	0.92 (0.26 to 3.16)
Regulatory agency			
No	169 (95)	92 (97)	Reference
Yes	9 (5)	3 (3)	1.55 (0.20 to 10.62)
United Nations agency			
No	159 (89)	80 (84)	Reference
Yes	19 (11)	15 (16)	1.16 (0.39 to 3.43)
Other			
No	165 (93)	90 (95)	Reference
Yes	13 (7)	5 (5)	1.03 (0.28 to 3.45)
Participant's Country of Work			
Country's eligibility for Gavi Funding (2023)			
Not eligible	128 (72)	77 (81)	Reference
Eligible	50 (28)	18 (19)	1.39 (0.35 to 5.21)
Country's World Bank Classification (2023)			
High income	36 (20)	32 (34)	Reference
Upper middle income	54 (30)	31 (33)	0.68 (0.17 to 2.52)
Lower middle income	65 (36)	25 (26)	0.61 (0.15 to 2.34)
Low income	23 (13)	7 (7)	0.62 (0.10 to 3.87)

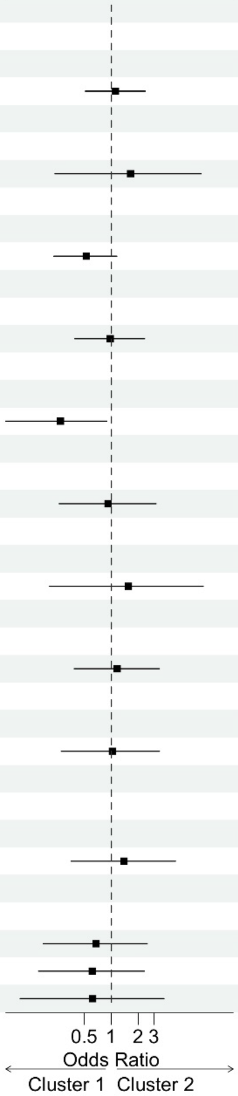

References

1. Boppana SB, van Boven M, Britt WJ, Gantt S, Griffiths PD, Grosse SD, et al. Vaccine value profile for cytomegalovirus. Vaccine. 2023 Nov;41 Suppl 2:S53–75.
2. World Health Organization. Dengue vaccines: WHO position paper – September 2018. Wkly Epidemiol Rec [Internet]. 2018;36(93):457–76. Available from: https://www.who.int/publications/i/item/who-wer9335-457-476
3. Wellcome Trust. Vaccines to tackle drug resistant infections An evaluation of R&D opportunities [Internet]. 2018 [cited 2024 June 20]. Available from: https://vaccinesforamr.org/wp-content/uploads/2018/09/Vaccines_for_AMR.pdf
4. World Health Organization. WHO Preferred Product Characteristics for Group A Streptococcus Vaccines [Internet]. 2018 [cited 2024 June 20]. Available from: https://www.who.int/publications-detail-redirect/who-preferred-product-characteristics-for-group-a-streptococcus-vaccines
5. World Health Organization. WHO Preferred Product Characteristics for Group B Streptococcus Vaccines [Internet]. 2017 [cited 2024 June 20]. Available from: https://apps.who.int/iris/rest/bitstreams/1087677/retrieve
6. Minervax ApS. Study to Evaluate the Safety, Tolerability and Immunogenicity of Three Doses of GBS Vaccine in Elderly Participants: NCT05782179 [Internet]. 2023 [cited 2024 June 20]. Available from: https://classic.clinicaltrials.gov/ct2/show/NCT05782179
7. Phelps CC, Walker CM, Honegger JR. Where to Next? Research Directions after the First Hepatitis C Vaccine Efficacy Trial. Viruses. 2021 Jul;13(7):1351.
8. Pevion Biotech Ltd. GeneCure Biotechnologies. A Phase I Trial of an Immunotherapy (HCVaxTM) in Chronic Hepatitis C Infected Patients: NCT00445419 [Internet]. 2007 [cited 2024 June 20]. Available from: https://classic.clinicaltrials.gov/ct2/show/NCT00445419
9. World Health Organization. WHO preferred product characteristics for monoclonal antibodies for HIV prevention [Internet]. 2022 [cited 2024 June 20]. Available from: https://www.who.int/publications/i/item/9789240045729
10. World Health Organization. WHO Preferred Product Characteristics for Next-Generation Influenza Vaccines [Internet]. 2017 [cited 2024 June 20]. Available from: https://www.who.int/publications/i/item/9789241512466
11. Kumar CK, Sands K, Walsh TR, O'Brien S, Sharland M, Lewnard JA, et al. Global, regional, and national estimates of the impact of a maternal *Klebsiella pneumoniae* vaccine: A Bayesian modeling analysis. PLoS Med. 2023 May;20(5):e1004239.
12. Dangor Z, Benson N, Berkley JA, Bielicki J, Bijsma MW, Broad J, et al. Vaccine value profile for *Klebsiella pneumoniae*. Vaccine. 2024 Jul;42(19S1):S125-S141.
13. Wantuch PL, Rosen DA. *Klebsiella pneumoniae*: adaptive immune landscapes and vaccine horizons. Trends Immunol. 2023 Oct;44(10):826–44.
14. Kaye PM, Matlashewski G, Mohan S, Le Rutte E, Mondal D, Khamesipour A, et al.

Vaccine value profile for leishmaniasis. Vaccine. 2023 Nov;41:S153–75.

15. Schrager LK, Chandrasekaran P, Fritzell BH, Hatherill M, Lambert PH, McShane H, et al. WHO preferred product characteristics for new vaccines against tuberculosis. The Lancet Infectious Diseases. 2018 Aug;18(8):828–9.

16. World Health Organization. Preferred Product Characteristics for Therapeutic Vaccines to Improve Tuberculosis Treatment Outcomes [Internet]. 2019 [cited 2024 June 20]. Available from: https://www.who.int/publications/i/item/WHO-IVB-19-05

17. World Health Organization. PDVAC (virtual) meeting on invasive non-typhoidal *Salmonella* (iNTS) vaccines: Summary and outcomes from PDVAC closed discussion [Internet]. 2022 [cited 2024 June 20]. Available from: https://cdn.who.int/media/docs/default-source/immunization/pdvac/pdvac-2022/final-summary-outcomes-pdvac-closed-discussion-feb-2022.pdf?sfvrsn=f62d9b5d_5

18. Armah G, Lopman BA, Vinjé J, O'Ryan M, Lanata CF, Groome M, et al. Vaccine value profile for norovirus. Vaccine. 2023 Nov;41:S134–52.

19. World Health Organization. Malaria vaccines: preferred product characteristics and clinical development considerations. [Internet]. 2022 [cited 2024 June 20]. Available from: https://www.who.int/publications/i/item/9789240057463

20. World Health Organization. WHO Preferred Product Characteristics for Respiratory Syncytial Virus (RSV) Vaccines [Internet]. 2017 [cited 2024 June 20]. Available from: https://apps.who.int/iris/bitstream/handle/10665/258705/WHO-IVB-17.11-eng.pdf?sequence=1

21. World Health Organization. WHO preferred product characteristics of monoclonal antibodies for passive immunization against respiratory syncytial virus (RSV) disease [Internet]. 2021 [cited 2024 June 20]. Available from: https://www.who.int/publications/i/item/9789240021853

22. World Health Organization. WHO Preferred Product Characteristics for Vaccines against *Shigella* [Internet]. 2020 [cited 2024 June 20]. Available from: https://www.who.int/publications/i/item/9789240036741

23. Hausdorff WP, Anderson JD 4th, Bagamian KH, Bourgeois AL, Mills M, Sawe F, et al. Vaccine value profile for *Shigella*. Vaccine. 2023 Nov;41 Suppl 2:S76–94.

24. World Health Organization. 2021 Bacterial vaccines in clinical and preclinical development: an overview and analysis. [Internet]. 2022 [cited 2024 June 20]. Available from: https://www.who.int/publications/i/item/9789240052451

25. AridisPharma. Aridis Pharmaceuticals Announces Third Quarter 2023 Financial Results and Business Update [Internet]. 2023 [cited 2024 Apr 22]. Available from: https://www.biospace.com/article/releases/aridis-pharmaceuticals-announces-third-quarter-2023-financial-results-and-business-update/

26. BioSpace [Internet]. 2024 [cited 2024 Oct 2]. Aridis Provides Corporate Update - June 24, 2024. Available from: https://www.biospace.com/aridis-provides-corporate-update-june-24-2024

27. R Core Team (2023). R: A Language and Environment for Statistical Computing. R

Foundation for Statistical Computing. Vienna, Austria.

28. Kassambara A, Mundt F. factoextra: Extract and Visualize the Results of Multivariate Data Analyses. 2020 [cited 2024 June 20]. Available from: https://CRAN.R-project.org/package=factoextra. R package version 1.0.7.

29. Carey VJ. gee: Generalized Estimation Equation Solver, 2022 [cited 2024 June 20]. Available from: https://CRAN.R-project.org/package=gee. R package version 4.13-25.

30. Thorndike RL. Who belongs in the family? Psychometrika. 1953;18(4):267–76.

31. Rousseeuw P. Silhouettes: A Graphical Aid to the Interpretation and Validation of Cluster Analysis. J Comput Appl Math. 1987 Nov 1;20:53–65.

32. Tibshirani R, Walther G, Hastie T. Estimating the Number of Clusters in a Data Set Via the Gap Statistic. Journal of the Royal Statistical Society Series B: Statistical Methodology. 2001 Jul 1;63(2):411–23.

33. Charrad M, Ghazzali N, Boiteau V, Niknafs A. NbClust: An R Package for Determining the Relevant Number of Clusters in a Data Set. Journal of Statistical Software. 2014 Nov 3;61:1–36.
